# Synthesis of the Tetracyclic Core of the Daphlongeranines

**DOI:** 10.1021/acs.orglett.5c03362

**Published:** 2025-10-07

**Authors:** Benjamin D. A. Shennan, Peter W. Smith, Yusuke Ogura, Eddy A. Källström, Moses Moustakim, Tudor Balan, Darren J. Dixon

**Affiliations:** Department of Chemistry, 6396 University of Oxford, Chemistry Research Laboratory, 12 Mansfield Road, Oxford OX1 3TA, U.K.

## Abstract

The first synthesis of the tetracyclic core of the daphlongeranine natural product family is reported. Containing a tricyclic core unique to this subfamily of the *Daphniphyllum* alkaloids and featuring two quaternary carbons, this 11-step synthetic route featured the application of a three-step spirocyclization strategy and development of a new intramolecular Pd-catalyzed cyclization reaction. Additionally, the route included the application of an XAT-initiated Giese addition and demonstration of an enantioselective synthesis of the bicyclic core.

## Graphical abstract

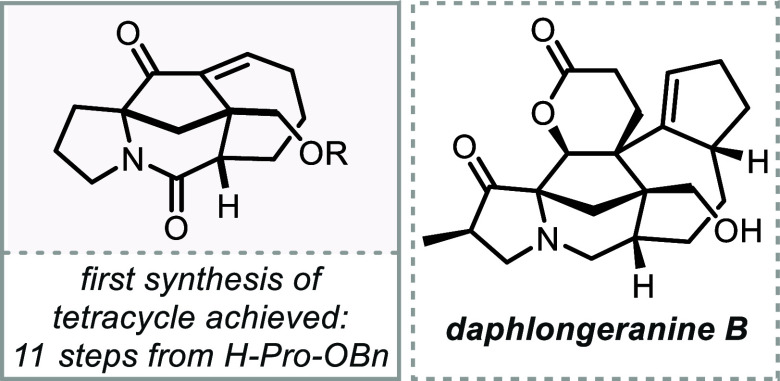

The *Daphniphyllum* alkaloids have enchanted the synthetic community since the earliest structural assignment from Hirata in 1966.^1^ From these first steps, the field has driven a swathe of synthetic discovery and served as a lodestar for not only practitioners targeting complex cage-like alkaloids but, indeed, the whole synthetic discipline. This large and inspiring body of work has been reviewed by Heathcock, Kobayashi and, subsequently, Hanessian and others.^2–7^

The synthesis of *Daphniphyllum* alkaloids quickly rose to prominence on the back of Heathcock’s concerted program targeting both biomimetic and traditional total synthetic approaches, achieving the synthesis of six members of the family.^8–13^ Following this work, despite a few reported approaches to core motifs,^3^ it was not until 16 years later, in 2011, that Carreira achieved the next total synthesis with the completed synthesis of daphmanidin E.^14^ Since this work, notable syntheses have been achieved by the groups of A. Li,^15–19^ Smith,^20^ Hanessian,^21^ Dixon,^22,23^ Xu,^6,24–26^ Sarpong,^27–29^ among others.^30–37^ These leaps forward span multiple subfamilies and include impressive rearrangements between different skeletal subfamilies.

However, one notable omission from this body of work is any reported progress toward the daphlongeranine subfamily,^38^ containing two members, daphlongeranine A and B (Scheme 1A). Isolated by Hao from the fruits of *D. longeracemosum*, this unique structural outlier is characterized by an embedded 1-azaspiro[4.4]­nonane bicyclic motif, which is proposed to arise biosynthetically from a 1,2-N atom shift from a fused (6,5) motif. Further structural features include three quaternary centers, and a “northern” lactone motif decorating the central congested cyclopentane moiety, adorned with substitution at seven out of ten possible sites.

**Scheme 1:**
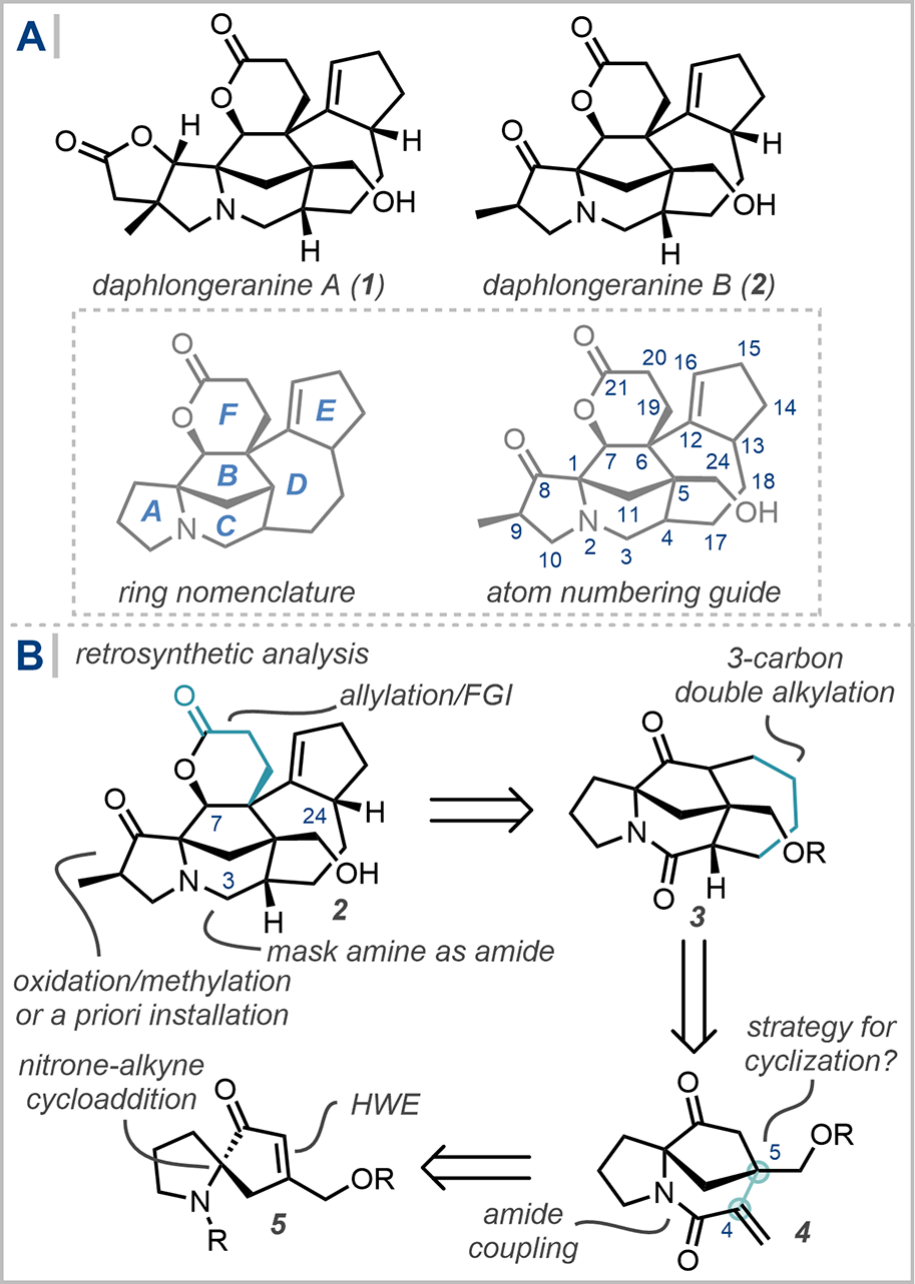
(A) The daphlongeranines; (B) Retrosynthetic Analysis of Daphlongeranine B

Given this distinctive structure and the total absence of literature reports toward their synthesis,^39^ we considered that an approach to the daphlongeranine core would be a worthwhile objective for advancing the field of *Daphniphyllum* alkaloid total synthesis while also more widely impacting synthetic approaches toward other congested sp^3^-rich architectures.

Initial retrosynthetic simplifications included masking the tertiary amine as a C_3_ amide, the chemoselective reduction of which has been previously deployed in *Daphniphyllum* total synthesis,^40^ and protection of the C_24_ alcohol (Scheme 1B). At the outset of this study, we recognized that the lactone could be retraced to the C_7_ ketone, with the three carbons coming from allylation or Michael addition of the ketone. Installation of the cyclopentene could arise from β/γ oxidation of the ketone and subsequent alkylation/cyclization and appropriate functional group interconversions, reminiscent of our cyclopentenone strategy in the total synthesis of himalensine A.^22^ At this stage, the pyrrolidinone A ring was simplified to a pyrrolidine, believing that the requisite functionality could be installed either via the application of the now-significant arsenal of C–H oxidation strategies or via early incorporation of the necessary functionality.

This led to complex tetracycle **3** as a key strategic target. Retrosynthetic excision of a three-carbon unit from the seven-membered D ring returned a simplified tricyclic core. As discussed in this report and determined after significant experimentation, a 1,1-disubstituted alkene motif at C_4_ was most amenable to C–C bond formation from the corresponding functionalized 1-azaspiro[4.4]­nonenone bicyclic core. This simplified bicycle was proposed to arise from a three-step sequence reported by our group, featuring a key reductive spirocyclization cascade, and retracing the synthesis back to a proline-derived starting material.^41^

For the forward synthesis, l-proline benzyl ester hydrochloride was free-based and oxidized to the corresponding 1,2-nitrone (Scheme 2). In our initial report detailing the synthesis of spirocyclic pyrrolidines, Na_2_WO_4_- or MeReO_3_-catalyzed oxidations were employed;^42,43^ however, these were substituted for a metal-free Oxone-mediated oxidation which afforded a similar yield but with greater reproducibility.^44^ Subsequent thermal 1,3-dipolar cycloaddition with alkyne **8** occurred in >80% yield. Exchange of the benzyl ester for the desired ketophosphonate moiety, by addition of the corresponding Li-phosphonate, was very efficient, setting the stage for the key spirocyclization reaction. Following treatment with *n-*BuLi to protect the phosphonate moiety from reduction, then sodium naphthalenide (NaNap) to effect N–O reductive cleavage, the desired spirocyclic *N*-Boc-amine **11** could be afforded in good yield, after basic aqueous work up and *in situ* Boc-protection.^41^

**Scheme 2:**
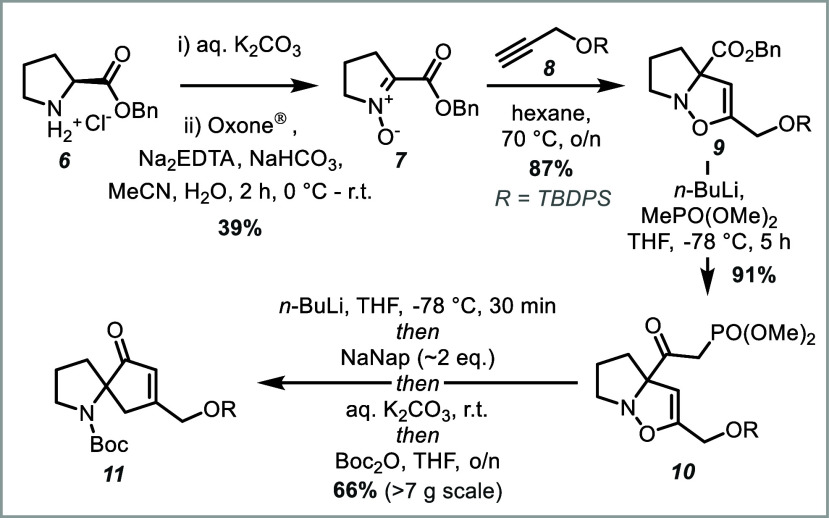
Synthesis of Spirocyclic Amine Core **11**

At this point, attention turned to the installation of a functional handle amenable for the C_4_–C_5_ cyclization (Scheme 3). Amide substrates could be generated trivially via Boc-deprotection and amide coupling. Hence, a broad survey of potential cyclization modes was conducted, including but not limited to Michael reactivity, radical addition,^45^ MHAT chemistry, and cyclopropanation via diazo precursors.^33,46–48^

**Scheme 3:**
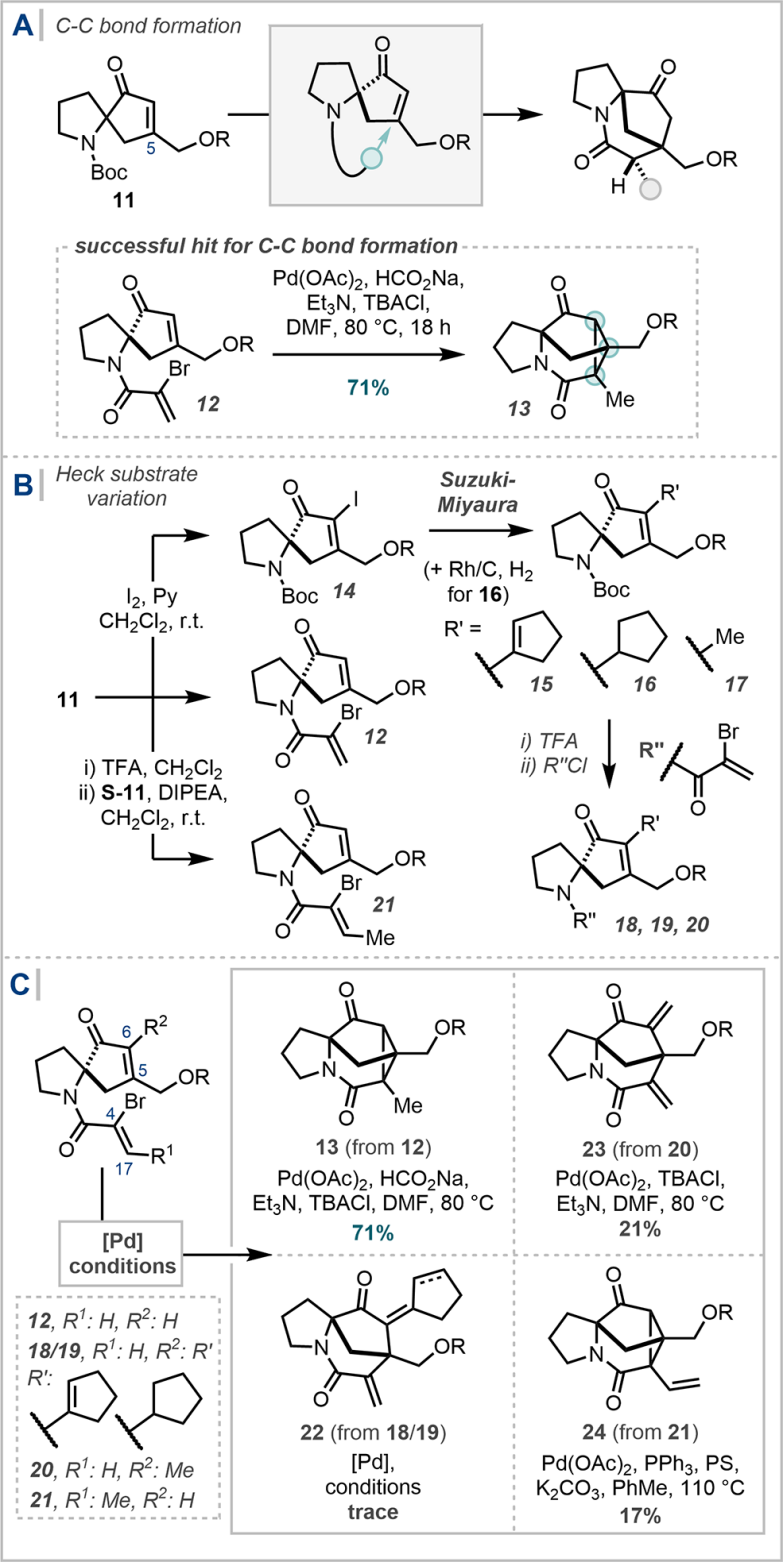
(A) Successful C–C Bond Formation by Reductive Heck Reaction; (B) Synthesis of Heck Cyclization Precursors; (C) Exploration of Heck-Type Reactivity for Cyclization^a^

a PS, Proton-Sponge; R = TBDPS.

After extensive investigation, with generally little to no observation of desired reactivity, successful C–C bond formation was observed with the treatment of α-bromoacrylamide **12** under reductive Heck conditions (Pd­(OAc)_2_, HCO_2_Na, Et_3_N, TBACl, DMF, 80 °C, 18 h – Jeffrey’s conditions, Scheme 3A).^49^ Remarkably, rather than undergoing solely a single carbopalladation event and subsequent reductive termination, the resulting palladium enolate underwent a further intramolecular carbopalladation, and reductive termination occurred from the alkyl-Pd species to afford Me-cyclopropane **13**. While this additional cyclopropane ring-forming reactivity was undesired, the reaction demonstrated proof-of-concept for the application of Pd-mediated cyclization in forging the C_4_–C_5_ bond.^50^

Variation of either the α position of the cyclopentenone (C_6_) or the β position of the unsaturated amide (C_17_) was proposed to divert the course of the reaction to afford more useful intermediates than cyclopropane **13**. Initially, the α-installation of a cyclopentane/ene was investigated, reflecting the daphlongeranine E ring. Iodination and subsequent Suzuki-Miyaura coupling with cyclopenteneboronic acid pinacol ester was facile, affording **15**.^51^ Rh/C-catalyzed hydrogenation was effective for accessing the cyclopentane analogue **16**. Following synthesis of the corresponding α-bromoacrylamides (**18**, **19**), investigation of the Heck-type cyclization confirmed that, under either Heck or reductive Heck conditions, no or only trace cyclization was observed.^52,53^

Alternatively, Suzuki-Miyaura coupling could install a methyl group at C_6_,^54^ offering a potential β-hydrogen elimination pathway following the first carbopalladation in the Heck reaction. While, under select Heck conditions,^49^ the corresponding α-bromoacrylamide **20** did afford the targeted diene product, the yield was low and could not be raised above 21%. Equally, varying the β position of the unsaturated amide, crotonamide **21** was trialled in the Heck reaction; pleasingly, vinylcyclopropene **24** was observed, however only in a low yield that made application to the total synthesis unfeasible.^52,53^

Given these challenges, the original reductive Heck cyclopropanation reaction was revisited in the hope that, by careful modification of the reaction conditions, the pathway could be deviated from a double carbopalladation to a single Pd-mediated conjugate addition (Scheme 4A, entry 1; see also Supporting Information S1). Initially, by varying the H-source from HCO_2_Na to either *i*-PrOH or AcOH, a low yield of the desired tricyclic product **4** was observed (entries 2 and 3). In cases where a carboxylic acid was employed as the H-source, a non-negligible degree of γ-coupled product **26** was observed, likely from the corresponding (extended) enol coupling mode (entry 3). The yield of **4** could be further increased to 37% by switching TBACl to TBABr and increasing the equivalents from one to three (entry 5). Investigating alternative reductive Heck conditions led predominantly to direct reduction, i.e., proto-depalladation of the initial oxidative addition intermediate (entry 4).

**Scheme 4:**
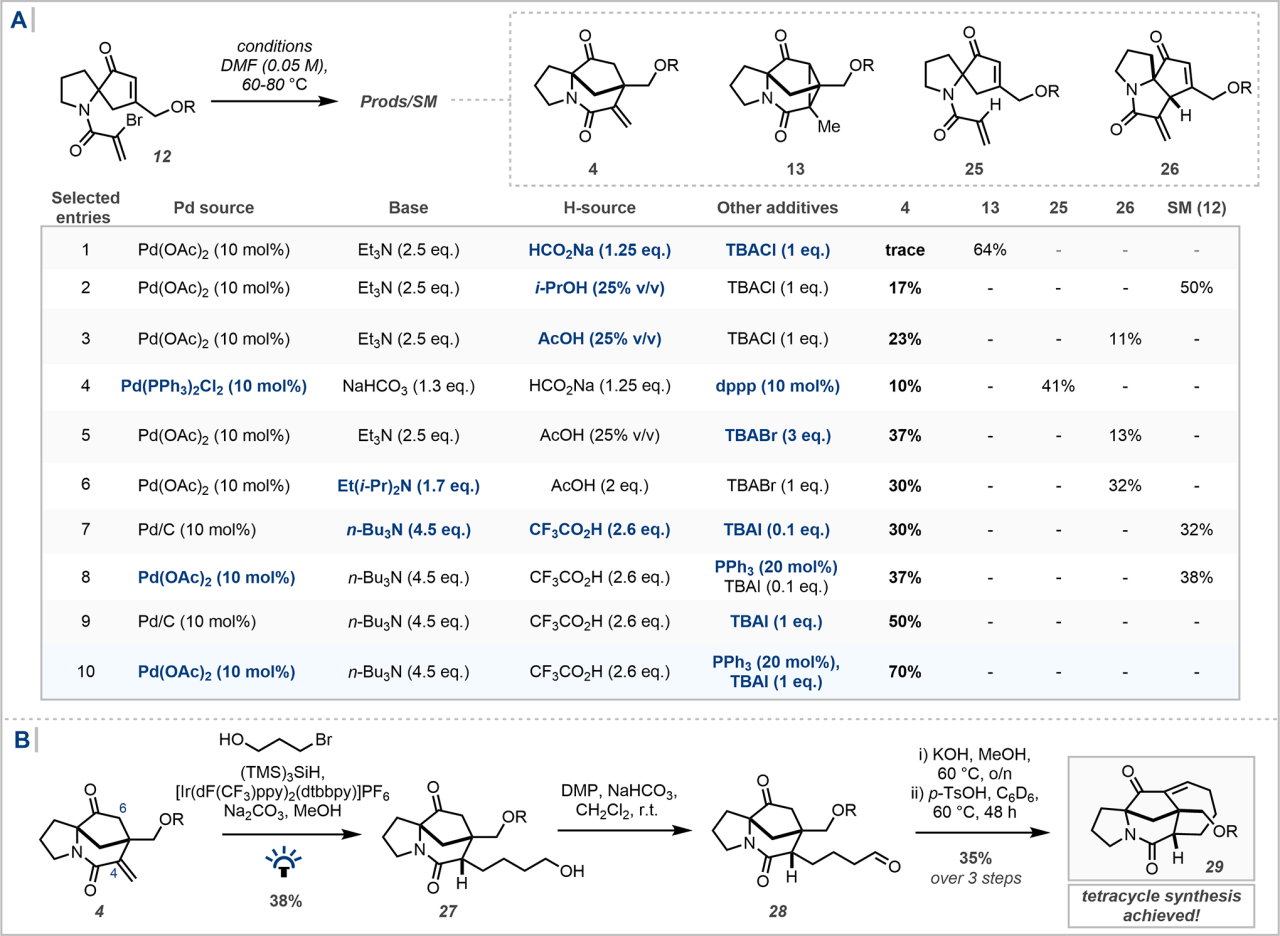
(A) Optimization of the Pd-Mediated Conjugate Addition for the Synthesis of Tricyclic Core **4**; (B) Advancement of the Synthesis to Tetracycle **29** ^a^

a Dppp, 1,3-bis­(diphenylphosphino)­propane; R = TBDPS.

In 1989, Cacchi reported that the combination of a tertiary amine and strong acid such as trifluoroacetic acid, in the presence of *tert*-butylammonium iodide (TBAI) as a phase transfer catalyst, led to preferential formation of conjugate addition products in intermolecular Heck reactions, over traditional Heck-type vinyl substitution products.^55^ Trialling these conditions gave a comparable yield to the previous optimal conditions but notably with considerable unreacted starting material observed (32%, entry 7). Increasing the quantity of TBAI and inclusion of PPh_3_ as a ligand led to a substantial increase in the yield of **4** to 70% NMR yield (entries 8, 9, and 10). It should be noted that XAT-mediated radical cyclization and organocuprate formation were also attempted, and the Pd-mediated conditions proved singularly effective (See Supporting Information S2).

With robust access to a tricyclic core containing differentiated functionality at C_4_ and C_6_, the installation of the seven-membered ring could then be investigated (Scheme 4B). In order to minimize nonstrategic protecting group manipulations, the unprotected three-carbon unit 1-bromopropan-3-ol was investigated under XAT-mediated radical addition. Initially, Co-catalyzed conditions reported by Escobar and Johannes afforded the desired product in a low yield (16%, 60% recovered SM);^56^ however, photocatalytic silyl radical-mediated conditions proved optimal,^57^ installing the desired three-carbon unit in moderate yield and good diastereoselectivity (60% crude NMR yield, 5.1 d.r., 38% isolated).

Oxidation to the aldehyde could be conducted trivially with Dess-Martin Periodinane (DMP), setting the stage for the intramolecular aldol reaction to close the seven-membered ring. Treatment with *p*-TsOH led to observation of an undesired dimeric product. Alternatively, treatment with methanolic hydroxide and heating lead to cyclization to a mixture of the aldol product and the corresponding elimination product. Heating of this mixture with *p*-TsOH in C_6_D_6_ led to smooth conversion of the aldol product to the desired enone **29** in 35% yield over three steps.

Emboldened by the successful synthesis of the tetracyclic core of the daphlongeranines, an enantioselective synthesis of spirocycle **11** was investigated in order to demonstrate the applicability of the ensuing strategy for an enantioselective synthesis of the natural products (Scheme 5). Enantioselective and diastereoselective variants of the nitrone/alkyne 1,3-dipolar cycloaddition were investigated but were unsuccessful in affording the corresponding isoxazoline with high enantioselectivity or diastereoselectivity. Accordingly, a new strategy was developed, inspired by work from Kałuża in which alkylation and Parham-type cyclization of Seebach adducts was employed to access spiro-indane-2,2′-pyrrolidine containing ligands.^58,59^ By modification of literature procedures, four-carbon allylic electrophile **31** was accessed in two steps. Alkylation of the Seebach adduct occurred in moderate yield but good diastereoselectivity. Treatment with *t*-BuLi effected rapid lithium–iodine exchange and concomitant cyclization to spirocycle **33** in good yield. A three-step γ-Rubottom protection sequence efficiently completed the synthesis of enantioenriched spirocycle **11.**

**Scheme 5:**
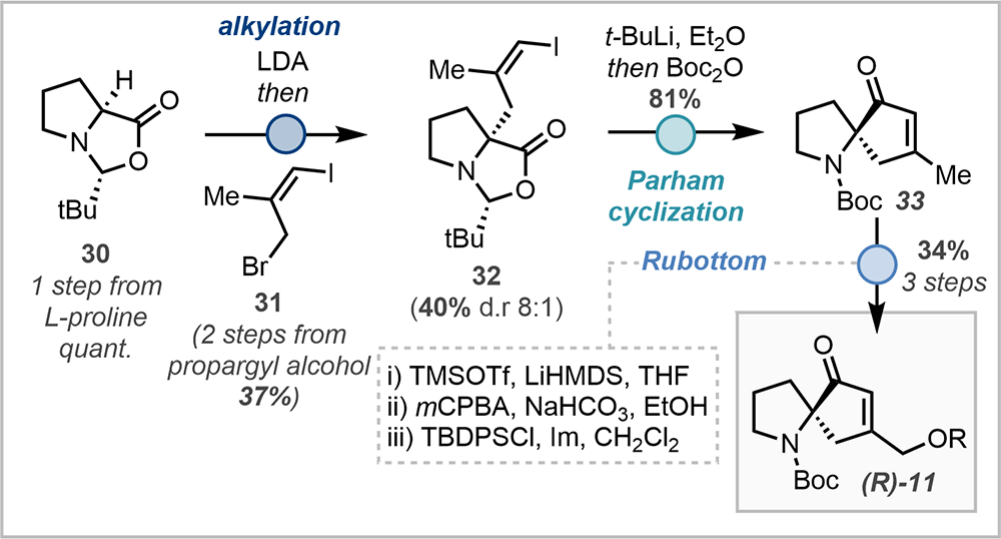
Proof-of-Concept for an Enantioselective Synthesis of Bicyclic Core **11** ^a^

a R = TBDPS.

In summary, an 11-step synthesis of a tetracyclic intermediate en route to the daphlongeranine natural products has been developed, and proof-of-concept for an enantioselective synthesis has been demonstrated. The synthesis features formation of two quaternary carbons and hinges upon the strategic application of a three-step spirocyclization procedure and on the development of a selective Pd-catalyzed conjugate addition reaction.

## Supplementary Material



## Data Availability

The data underlying this study are available in the published article and its Supporting Information.

## Notes

Synthetic procedures and full characterization data of all compounds (PDF)
