# Supplementary material for: Synthesis of the Tetracyclic Core of the Daphlongeranines
Source: Org Lett. 2025 Oct 7;27(41):11485–90. doi: 10.1021/acs.orglett.5c03362 (PMC12538578; doi:10.1021/acs.orglett.5c03362)
Supplement: Supplementary file 1 [file ol5c03362_si_001.pdf]

Supporting Information

for

Synthesis of the tetracyclic core of the daphlongeranines

Benjamin D. A. Shennan, Peter W. Smith, Yusuke Ogura, Eddy A. Källström, Moses Moustakim, Tudor Balan, Darren J. Dixon*

Department of Chemistry, Chemistry Research Laboratory, University of Oxford,

12 Mansfield Road, Oxford OX1 3TA, UK

E-mail: darren.dixon@chem.ox.ac.uk

General experimental

General techniques: Procedures using oxygen- and/or moisture-sensitive materials were performed with anhydrous solvents under an atmosphere of anhydrous argon or nitrogen in oven-dried flasks, using standard Schlenk techniques. Room temperature refers to 22 ± 2 °C. Reaction temperatures refer to external temperatures. Heating was performed using oil baths and DrySyn heating blocks. Photoirradiation was conducted in a HepatoChem EvoluChem™ PhotoRedOx Box equipped with an EvoluChem™ 450PF 450 nm 18 W blue LED light source.

Solvent and reagents: Commercially available reagents, ligands and deuterated solvents were purchased from Sigma Aldrich, Alfa Aesar, Acros Organics, Fluorochem, BLDpharm and Strem Chemicals and, unless otherwise stated, were used without further purification. Dry solvents were either collected fresh from an mBraun SPS–800 solvent purification system after having passed through anhydrous alumina columns (powder ~150 mesh, pore size 58 Å, basic, Sigma-Aldrich). Anhydrous THF, PhMe, CH_2Cl_2 and select other solvents were obtained from Acros Organics stored under AcroSeal® over molecular sieves.

Thin layer chromatography (TLC): Analytical thin-layer chromatography was performed using Merck aluminium-backed DC60 F254 plates (particle size 0.2 mm) and visualised using a combination of UV light (254 nm) and aqueous basic potassium permanganate stain. Where acid-sensitive or basic compounds were under analysis, the TLC plate was fully submerged in an appropriate Et_3N-containing eluent mixture, removed, briefly allowed to dry and then the sample was loaded and run in the usual manner. Where compounds are isolated by PTLC, this was conducted using either a whole plate of the same type described for TLC or a 10 cm x 10 cm section of the plate for smaller scale purifications. The plate was run in a glass chamber capped with aluminium foil, using ~300 mL/30mL for large/small plates. Bands were visualised using UV light (254 nm), scratched off the plate and the compound was eluded using $CHCl_3$:MeOH (9:1). Where the compound lacked a visible chromophore, a thin strip (0.5 – 1 cm) was cut from the side of the plate, visualised using $KMnO_4$ and the visible bands were extrapolated to the remainder of the plate.

Column chromatography: Purification by column chromatography was carried out using Merck silica gel 60 (particle size 43–60 μm).

Nuclear magnetic resonance (NMR): Proton, carbon and fluorine NMR spectra were recorded on Bruker 400, 500 and 600 MHz spectrometers. Chemical shifts for protons are reported in parts per million downfield from $Si(CH_3)_4$ and are referenced to residual protium in the deuterated solvent. NMR data are presented in the following format: chemical shift (multiplicity [app = apparent, br = broad, d = doublet, t = triplet, q = quartet, p = quintet, h = sextet, hept = septet, dd = doublet of doublets, dt = doublet of

triplets, dq = doublet of quartets, ddd = doublet of doublet of doublets, m = multiplet], number of equivalent nuclei by integration, coupling constant [in Hz]). Stereochemical assignments deduced from nOe signals are depicted after the analytical data as coloured arrows between the sites in question. Diastereomeric ratios were determined by analysis of ^{1}H NMR spectrum of the unpurified mixture following work-up unless otherwise specified. Crude NMR yields were determined from quantitative ^{1}H spectra using either 1,3,5-trimethylbenzene, 1,3,5-trimethoxybenzene or 1,2,4,5-tetramethylbenzene as internal standards.

Infrared spectroscopy (IR, neat or thin film) was carried out on a Bruker Tensor 27 FT–IR spectrometer within an internal calibration range of 4000 – 600 cm^{-1}. Selected diagnostic absorption maxima (ν_{max}) are reported in wavenumbers (cm^{-1}).

High resolution mass spectrometry (HRMS) data were recorded on an ACQUITY I-Class PLUS UPLC System (Waters, Milford, MA, USA) coupled to an ACQUITY RDa mass spectrometer (Waters, Milford, MA, USA) equipped with an ESI probe, in positive ion mode. The flow rate was set to 0.300 mL/min using a 50% methanol (aq) + 0.1% formic acid eluent. Scan parameters were set as follows: analyser mode, full scan; scan range, 50-2000 m/z; scan rate, 2 Hz; cone voltage, 30 V; capillary voltage, 1.5 kV; desolvation temperature, 550 °C; and intelligent data capture, on. Some HRMS data were recorded on a Bruker μTOF mass spectrometer.

Optical rotations were recorded using a Perkin Elmer 241 optical activity polarimeter at 25 °C. Specific rotations $[\alpha]_D^{25}$ are reported in 10^{-1} $deg.cm^2.g^{-1}$, with concentrations (c) in g/100 mL, and D refers to the D-line of sodium (589 nm). All polarimeter measurements were taken in $CHCl_3$ unless otherwise stated.

Melting points were obtained on a Leica Galen III Hot-stage melting point apparatus and microscope and are reported uncorrected.

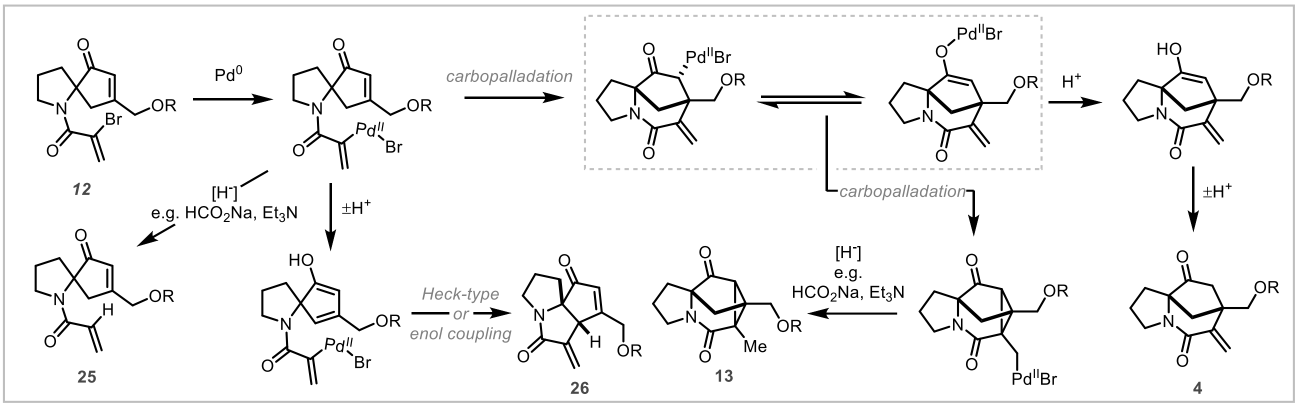

Scheme S1: Proposed mechanistic rationale for the products formed under different Heck reaction conditions. R = TBDPS

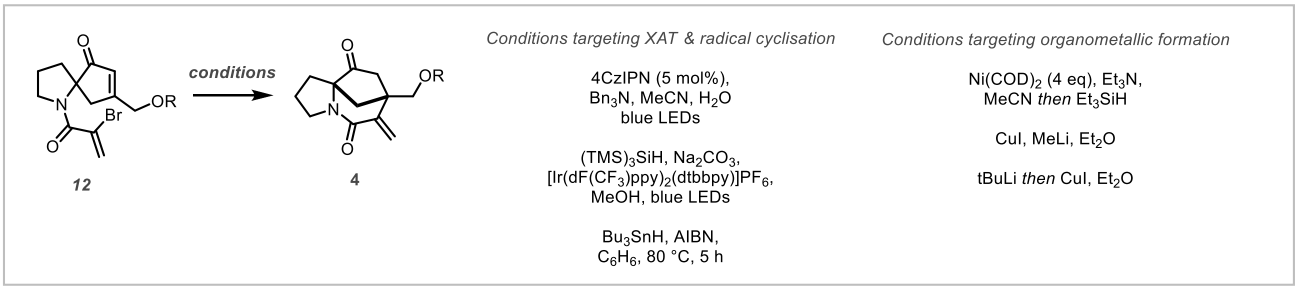

Scheme S2: Alternative conditions trialled for the cyclisation of bromoacrylamide **12**. R = TBDPS

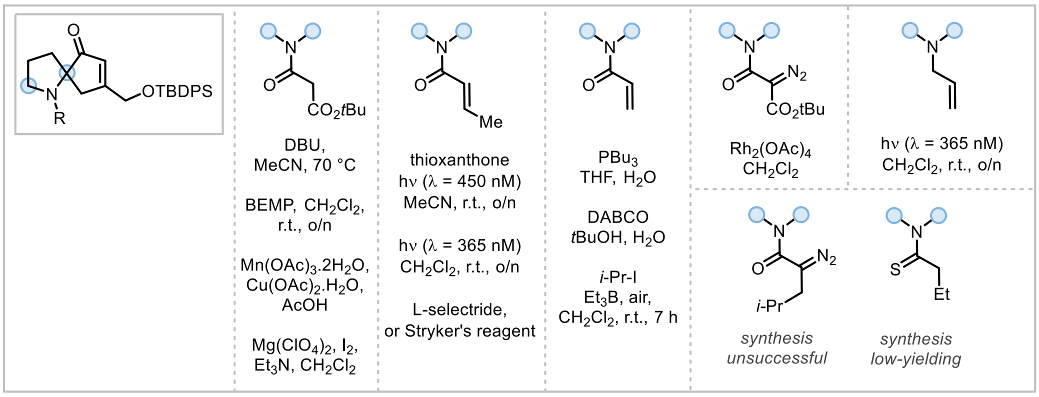

Scheme S3: Alternative substrates synthesised for the construction of the third ring and conditions trialled to effect cyclisation. Conditions shown afforded no desired product.

General procedures

General Procedure 1: *N*-Boc deprotection

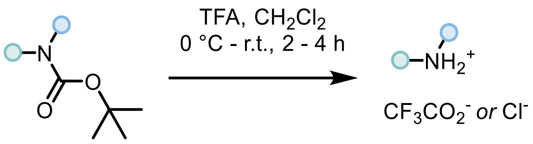

To a solution of *N*-Boc-protected compound in CH_2Cl_2 (0.05 M), cooled to 0 °C, was added TFA (25% v/v). The resulting solution was stirred for 1 hour at room temperature. TLC analysis (EtOAc) was employed to determine completion by observation of disappearance of starting material. One of the three following work ups were employed:

Work up 1: the pH was adjusted to >9 by the addition of sat. aq. Na_2CO_3 and the resulting biphasic mixture was extracted with $CHCl_3$:*i*-PrOH (3:1, 3 x 20 mL). The combined organics were dried with Na_2SO_4, filtered and the solvent was removed under reduced pressure.

Work up 2: The solvent was removed under reduced pressure and CH_2Cl_2 was added and removed under reduced pressure. This co-evaporation cycle was repeated four further times.

Work up 3: The solvent was removed under reduced pressure and the crude residue was dissolved with 4 M HCl in 1,4-dioxane and re-evaporated and this was repeated 3 times in order to obtain the crude HCl salt.

General Procedure 2: Acid chloride synthesis

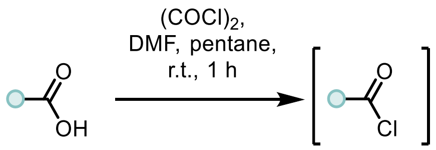

To a suspension or solution of carboxylic acid (1.0 eq.) in pentane (0.025 M) was added $(COCl)_2$ (4.75 eq.) and then DMF (1.05 eq.). Vigorous gas evolution and formation of an insoluble precipitate was observed and the reaction mixture was stirred for 1 hour at room temperature. The mixture was filtered and the filtrate was concentrated under reduced pressure. In cases of low M_w acid chloride products (e.g 2-bromo-acryloyl chloride), the mass upon evaporation was regularly monitored to ensure minimal evaporation of the desired product.

General Procedure 3: Acid chloride coupling

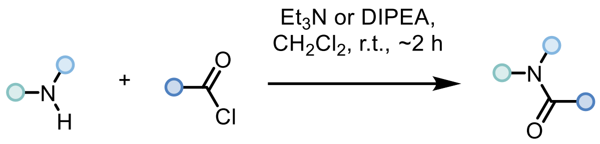

To a solution of freshly prepared amine (1 eq.) in CH_2Cl_2 (0.05 – 0.1 M), cooled to 0 °C, was added either Et_3N or DIPEA (4 eq.). The solution was stirred for 5−10 minutes before the addition of a solution of acid chloride (2 eq.) in CH_2Cl_2. The reaction mixture was allowed to come to room temperature and stirred until completion, as observed by TLC, typically 2 hours. Sat. aq. NH_4Cl was added and the biphasic mixture was diluted with H_2O and CH_2Cl_2. The layers were separated and the aqueous phase was extracted with CH_2Cl_2 a further two times. The combined organics were dried with Na_2SO_4, filtered and concentrated under reduced pressure. The resulting crude residue was purified by FCC (pentane:EtOAc).

Experimental procedures

Compound 6

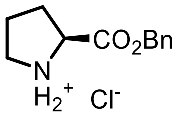

Following a modified literature procedure,[1] to a solution of (*S*)-proline (10 g, 87 mmol) in benzyl alcohol (174 mL), cooled to 0 °C, was added thionyl chloride (11.4 mL, 157 mmol) dropwise. The resulting solution was warmed to 95 °C and stirred for 6 hours. The reaction mixture was cooled to room temperature before the addition of Et_2O (~1.7 L). A white precipitate was observed and the reaction stirred overnight at room temperature. The mixture was then cooled to 0 °C, filtered and the solid residue was washed with cold Et_2O. The title compound was isolated as an off-white solid (18.0 g, 86%). All data were in agreement with the literature.[2]

^{1}H NMR (400 MHz, $CDCl_3$) δ 10.81 (s, 1H), 9.21 (s, 1H), 7.34 (m, 5H), 5.29 – 5.12 (m, 2H), 4.49 (dtd, *J* = 8.7, 6.3, 4.5 Hz, 1H), 3.61 – 3.42 (m, 2H), 2.46 – 2.31 (m, 1H), 2.22 – 1.87 (m, 3H).

^{13}C NMR (101 MHz, $CDCl_3$) δ 168.8, 134.6, 128.7, 128.7, 128.5, 68.4, 59.3, 46.0, 28.8, 23.5.

Compound 7

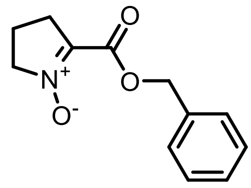

Following a literature procedure,[3] to a solution of compound **6** (4.0 g, 20 mmol) in a minimum amount of H_2O was added sat. aq. K_2CO_3 until the pH was observed to be >9. The aqueous phase was extracted with CH_2Cl_2 (3 x 200 mL) and the combined organics were dried with Na_2SO_4, filtered and the solvent was removed under reduced pressure. The resulting amine was dissolved in MeCN (32 mL) and THF (8 mL) and to this was added Na_2EDTA (0.01 M in H_2O, 28 mL) and $NaHCO_3$ (8.3 g, 99 mmol). The solution was cooled to 0 °C and Oxone® (12.7 g, 20.7 mmol) was added portionwise over 2 hours. After the addition, the mixture was stirred at 5 °C for 20 minutes before addition of EtOAc (20 mL) and H_2O (50 mL). The resulting biphasic mixture was extracted with CH_2Cl_2 and the combined organics were dried with $MgSO_4$, filtered and the solvent was removed under reduced pressure. The crude residue was purified by FCC (19:1 EtOAc:MeOH) to yield the title compound as a yellow oil (1.7 g, 39%). All data were in agreement with the literature.[2]

^{1}H NMR (400 MHz, $CDCl_3$) δ 7.44 – 7.26 (m, 5H), 5.29 (app. d, *J* = 2.0 Hz, 2H), 4.22 – 4.12 (m, 2H), 3.04 (ddt, *J* = 8.2, 6.5, 1.9 Hz, 2H), 2.23 – 2.10 (m, 2H).

^{13}C NMR (101 MHz, $CDCl_3$) δ 159.2, 135.4, 133.6, 128.6, 128.4, 128.4, 66.9, 66.7, 29.7, 16.8

Compound 8

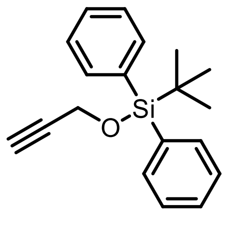

To a solution of propargyl alcohol (10 mL, 170 mmol) in CH_2Cl_2 (344 mL), cooled to 0 °C, was added imidazole (12.9 g, 189 mmol) and TBDPSCl (49 mL, 189 mmol). To solution was warmed to room temperature and stirred overnight before the addition of H_2O (344 mL). The resulting biphasic mixture was extracted with CH_2Cl_2 (3 x 200 mL) and the combined organics were dried with $MgSO_4$, filtered and concentrated under reduced pressure. The crude residue was purified by FCC (19:1 pentane:Et_2O) to yield the title compound as a white solid (42.8 g, 85%). All data were in agreement with the literature.[4]

^{1}H NMR (400 MHz, $CDCl_3$) δ 7.76 – 7.67 (m, 4H), 7.49 – 7.35 (m, 6H), 4.32 (d, *J* = 2.4 Hz, 2H), 2.38 (t, *J* = 2.4 Hz, 1H), 1.07 (s, 9H).

^{13}C NMR (101 MHz, $CDCl_3$) δ 135.6, 133.0, 129.9, 127.8, 82.0, 73.0, 52.5, 26.7, 19.2.

Compound 9

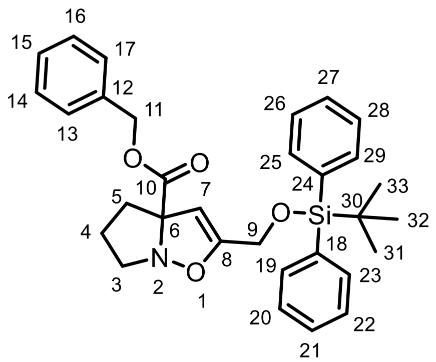

To a mixture of compound **7** (1.5 g, 6.8 mmol) in hexane (68 mL) was added compound **8** (4.0 g, 14 mmol). The reaction mixture was heated to reflux for 18 hours before being cooled to room temperature at which point the solvent was removed under reduced pressure. The crude residue was purified by FCC (2:1 pentane:Et_2O) to yield the title compound as a yellow oil (3.0 g, 87%).

^{1}H NMR (400 MHz, $CDCl_3$) δ 7.71 – 7.60 (m, 4H), 7.47 – 7.28 (m, 11H), 5.21 (d, *J* = 1.1 Hz, 2H), 4.79 (app. q, *J* = 1.2 Hz, 1H), 4.21 (app. q, *J* = 1.4 Hz, 2H), 3.32 (dd, *J* = 7.1, 5.7 Hz, 2H), 2.17 (dt, *J* = 12.6, 7.9 Hz, 1H), 2.02 (dt, *J* = 12.3, 5.9 Hz, 1H), 1.90 – 1.76 (m, 2H), 1.06 (s, 9H).

^{13}C NMR (101 MHz, $CDCl_3$) δ 173.0, 156.1, 136.0, 135.7, 135.7, 133.2, 133.1, 130.0, 130.0, 128.7, 128.3, 128.1, 127.9, 127.9, 96.3, 82.3, 67.0, 60.3, 58.0, 37.0, 26.9, 23.3, 19.4.

IR (thin film) ν_{max}/cm^{-1} 2932, 2859, 1735, 1107, 734, 699

HRMS (ESI) mass calculated for [M+H]+ ($C_{31}H_{36}O_4NSi$) requires *m/z* 514.2408, found *m/z* 514.2407

Compound 10

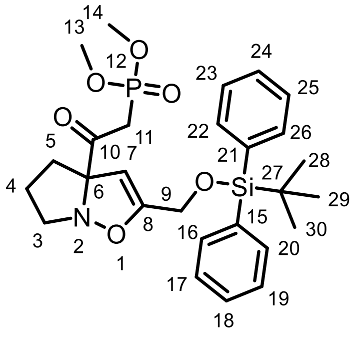

To a solution of dimethyl methylphosphonate (0.95 mL, 8.8 mmol) in THF (140 mL), cooled to −78 °C, was slowly added *n*-BuLi (3.4 mL, 2.5 M solution in hexanes, 8.5 mmol). The solution was stirred for 10 minutes before the dropwise addition of a solution of compound **9** (1.5 g, 2.9 mmol) in THF (5 mL). The reaction mixture was stirred at −78 °C for 4 hours before being warmed to room temperature and stirred for a further hour. Sat. aq. NH_4Cl (50 mL) was added and the biphasic mixture was extracted with EtOAc (3 x 100 mL). The combined organics were dried with Na_2SO_4, filtered and concentrated under reduced pressure. The crude residue was purified by FCC (2:1 to 1:0 pentane:EtOAc) to yield the title compound as a yellow oil (1.4 g, 91%).

^{1}H NMR (400 MHz, $CDCl_3$) δ 7.65 (m, 4H), 7.49 – 7.34 (m, 6H), 4.59 (d, *J* = 1.2 Hz, 1H), 4.22 (d, *J* = 1.2 Hz, 2H), 3.78 (app. dd, *J* = 11.2, 4.4 Hz, 6H), 3.73 – 3.60 (m, 1H), 3.33 – 3.15 (m, 3H), 2.27 (ddd, *J* = 12.7, 8.4, 7.1 Hz, 1H), 1.91 (ddd, *J* = 11.6, 6.8, 4.9 Hz, 1H), 1.86 – 1.70 (m, 2H), 1.06 (s, 9H).

^{13}C NMR (101 MHz, $CDCl_3$) δ 202.2 (d, J = 6.7 Hz), 157.2, 135.7, 135.6, 133.0, 133.0, 130.1, 130.1, 127.9, 96.4, 88.3 (d, J = 3.5 Hz), 60.0, 57.9, 53.2 – 52.9 (app. dd), 36.1 (d, J = 134.5 Hz), 33.9, 26.8, 23.3, 19.3.

IR (thin film) ν_{max}/cm^{-1} 2955, 2857, 1715, 1256, 1029, 702

HRMS (ESI) mass calculated for [M+H]+ ($C_{27}H_{37}O_6NPSi$) requires *m/z* 530.2122, found *m/z* 530.2122

Compound 11

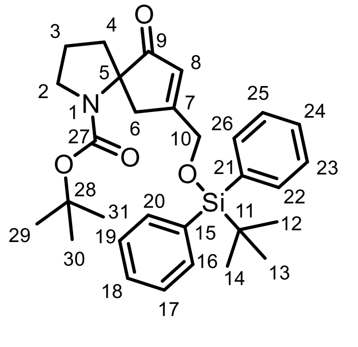

To a solution of ketophosphonate **10** (11.4 g, 21.5 mmol) in THF (430 mL), cooled to −78 °C, was added *n*-BuLi (14.1 mL, 1.6 M solution in hexanes, 22.6 mmol) and stirred for 30 minutes. A freshly prepared solution of NaNap (1 M in THF, see below for preparation) was added dropwise until a dark green colour persisted. As the persistence of the colour increased, TLC analysis was used to determine consumption of starting material. At the point when TLC analysis showed full consumption of starting material (~2.5 theor. eq., 54 mL), the cooling bath was removed and H_2O (400 mL) and sat. aq. K_2CO_3 (200 mL) were added. The dark brown-green solution rapidly turned light yellow with a white precipitate and was stirred at room temperature for 30 minutes. Boc_2O (28.0 g, 130 mmol) was added and the reaction mixture was stirred for 4 hours. The layers were separated and the aqueous phase was extracted with EtOAc (3 x 250 mL). The combined organics were dried with Na_2SO_4, filtered and concentrated under reduced pressure. The crude residue was purified by FCC (1:1 to 1:0 pentane:EtOAc) to yield the title compound as a colourless oil (7.2 g, 66%).

Note: upon completion of NaNap addition, the dark-green colour should just linger but should slowly fade if left longer. If the dark-green colour remains permanently, this represents over-addition and can reduce the yield.

^{1}H NMR (600 MHz, $CDCl_3$, mixture of rotamers) δ 7.65 (dddt, *J* = 8.1, 6.8, 3.5, 1.4 Hz, 4H), 7.47 – 7.42 (m, 2H), 7.39 (tdd, *J* = 7.3, 4.7, 3.7 Hz, 4H), 6.40 (p, *J* = 1.8 Hz, $1H_{min}$), 6.34 (p, *J* = 1.8 Hz, $1H_{maj}$), 4.53 (d, *J* = 17.7 Hz, $1H_{min}$), 4.45 (dd, *J* = 17.5, 1.7 Hz, $1H_{maj}$), 4.40 (dd, *J* = 17.5, 1.6 Hz, $1H_{maj}$), 4.35 (dd, *J* = 17.7, 1.7 Hz, $1H_{maj}$), 3.60 – 3.53 (m, $1H_{maj}$+$2H_{min}$), 3.53 – 3.48 (m, $1H_{maj}$), 3.01 (dd, *J* = 17.1, 2.1 Hz, $1H_{min}$), 2.84 (dd, *J* = 17.7, 1.9 Hz, $1H_{maj}$), 2.39 (dd, *J* = 17.7, 1.5 Hz, $1H_{maj}$), 2.33 (dd, *J* = 17.5, 1.3 Hz, $1H_{min}$), 2.07 (ddd, *J* = 12.0, 10.5, 6.4 Hz, $1H_{maj}$), 2.00 (dddd, *J* = 18.6, 12.2, 5.9, 3.6 Hz, 1H), 1.86 – 1.77 (m, 1H), 1.74 (ddd, *J* = 12.0, 6.1, 3.1 Hz, 1H), 1.69 (ddd, *J* = 11.5, 6.4, 3.0 Hz, $1H_{min}$), 1.43 (s, $9H_{min}$), 1.33 (s, $9H_{maj}$), 1.08 (s, $9H_{maj}$), 1.06 (s, $9H_{min}$).

^{13}C NMR (151 MHz, $CDCl_3$, mixture of rotamers) δ 207.6, 207.4, 176.1, 175.8, 153.5, 153.2, 135.7, 135.6, 135.6, 135.6, 133.1, 133.0, 132.9, 132.8, 130.2, 130.1, 130.0, 128.0, 128.0, 128.0, 126.3, 126.1, 80.4, 79.9, 69.0, 68.6, 64.1, 64.1, 48.3, 48.1, 42.9, 41.5, 40.2, 39.2, 28.6, 28.4, 26.8, 26.8, 24.0, 23.4, 19.4, 19.4.

IR (thin film) ν_{max}/cm^{-1} 2970, 2930, 1716, 1693, 1390, 1141, 703

HRMS (ESI) mass calculated for [M+Na]+ ($C_{30}H_{39}O_4NSiNa$) requires *m/z* 528.2541, found *m/z* 528.2539

Procedure for 0.1/1 M NaNap solution in THF: To a flask, purged with N_2 and evacuation heating cycles, was added naphthalene (1 eq.) and THF (0.1 or 1 M). Na metal (1.06 or 1.5 eq.) was added and the

flask was quickly put under N_2 atmosphere. The mixture was sonicated for 30 minutes and then stirred at room temperature for a further 1 hour. At this point, the resulting dark green solution was ready to use.

*For compounds **S-3**, **S-6**, **25** & **S-7**, stock solutions of amine hydrochloride **S-1** were produced using General Procedure 1 with Work-up 3, compound **11** (150 mg, 0.30 mmol) was converted to the crude amine hydrochloride salt and 0.10 mmol aliquots (assuming quant. Boc-deprotection) were used in the amide coupling reactions.*

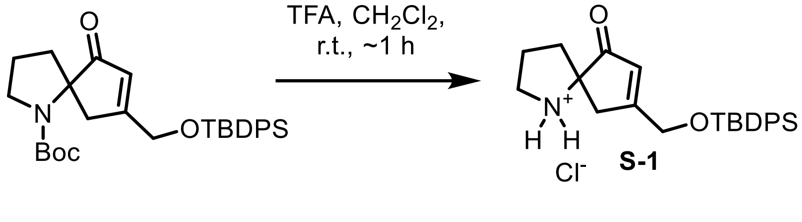

Compound S-2

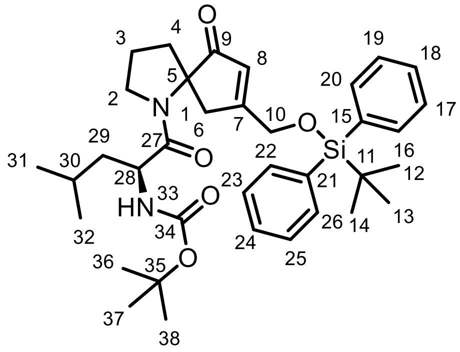

Following General Procedure 1 with work-up 3, compound **11** (49 mg, 0.10 mmol) was treated with TFA and the resulting crude amine hydrochloride salt was dissolved in CH_2Cl_2 (2 mL). DIPEA (51 µL, 0.29 mmol) was added and the solution was stirred for 10 minutes before cooling to 0 °C. Boc-L-leucine (22 mg, 0.097 mmol), HOBt·H_2O (30 mg, 0.19 mmol), and EDC (22 mg, 0.12 mmol) was added. The reaction mixture was stirred overnight at room temperature. Sat. aq. $NaHCO_3$ (2 mL) was added and the biphasic mixture was diluted with a further portion of CH_2Cl_2 (10 mL) and H_2O (10 mL). The layers were separated and the aqueous phase was extracted with CH_2Cl_2 (2 x 20 mL). The combined organics were dried with Na_2SO_4, filtered and concentrated under reduced pressure. The crude residue was purified by FCC (3:1 pentane:EtOAc) to yield the title compounds as colourless oils: **S-2A** (16 mg, 27%), **S-2B** (14 mg, 23%), mixture **A**:**B** (7.3 mg).

Isomer A

^{1}H NMR (400 MHz, $CDCl_3$) δ 7.65 (dt, *J* = 8.1, 1.7 Hz, 4H), 7.47 – 7.36 (m, 6H), 6.41 (t, *J* = 1.7 Hz, 1H), 5.02 (d, *J* = 9.2 Hz, 1H), 4.55 – 4.47 (m, 1H), 4.43 (td, *J* = 9.3, 4.6 Hz, 1H), 4.39 – 4.32 (m, 1H), 3.89 (t, *J*

= 8.5 Hz, 1H), 3.57 (td, *J* = 9.5, 6.1 Hz, 1H), 3.02 – 2.90 (m, 1H), 2.37 – 2.25 (m, 1H), 2.09 (dd, *J* = 9.8, 4.5 Hz, 1H), 2.07 – 1.91 (m, 2H), 1.79 – 1.67 (m, 2H), 1.48 (s, 2H), 1.41 (s, 9H), 1.06 (s, 9H), 0.99 (d, *J* = 6.5 Hz, 3H), 0.95 (d, *J* = 6.7 Hz, 3H).

^{13}C NMR (101 MHz, $CDCl_3$) δ 205.8, 175.5, 171.1, 155.9, 135.6, 135.6, 133.0, 132.9, 130.1, 130.0, 128.0, 128.0, 126.4, 79.6, 70.1, 64.0, 50.4, 48.2, 42.0, 41.3, 38.3, 28.5, 26.8, 24.7, 23.5, 21.9, 19.4.

IR (thin film) ν_{max}/cm^{-1} 3296, 2960, 1717, 1645

HRMS (ESI) mass calculated for [M+H]+ ($C_{36}H_{51}O_5N_2Si$) requires *m/z* 619.3562, found *m/z* 619.3563

[α]D (c = 0.93, $CHCl_3$) = 2.26.

Isomer B

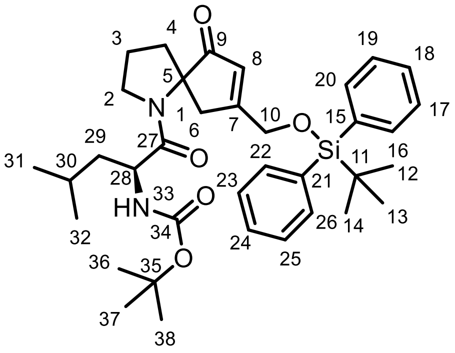

^{1}H NMR (400 MHz, $CDCl_3$) δ 7.59 (dq, *J* = 6.6, 1.4 Hz, 4H), 7.42 – 7.27 (m, 6H), 6.40 (t, J = 1.8 Hz, $1H_{min}$), 6.38 (t, J = 1.8 Hz, 1H), 5.18 (d, *J* = 9.0 Hz, 1H), 4.56 – 4.42 (m, 2H), 4.36 (dd, *J* = 17.7, 1.8 Hz, 1H), 3.85 – 3.68 (m, 1H), 3.68 – 3.52 (m, 1H), 2.87 (d, *J* = 17.8 Hz, 1H), 2.34 (d, *J* = 17.4 Hz, 1H), 2.21 – 2.10 (m, 1H), 2.10 – 2.00 (m, 1H), 1.95 (tt, *J* = 11.7, 5.4 Hz, 1H), 1.73 (m, 2H), 1.43 (m, 11H), 1.07 (s, 9H), 0.99 (d, *J* = 6.5 Hz, 3H), 0.91 (d, *J* = 6.7 Hz, 3H).

^{13}C NMR (101 MHz, $CDCl_3$) δ 205.7, 175.1, 170.8, 155.5, 135.7, 135.6, 133.0, 132.9, 130.1, 130.1, 128.0, 128.0, 126.2, 79.6, 69.7, 64.0, 50.3, 48.2, 43.2, 41.4, 38.0, 28.5, 26.8, 24.7, 24.6, 23.6, 22.2, 19.4.

IR (thin film) ν_{max}/cm^{-1} 3294, 2958, 1716, 1645, 703, 1428

HRMS (ESI) mass calculated for [M+H]+ ($C_{36}H_{51}O_5N_2Si$) requires *m/z* 619.3562, found *m/z* 619.3555

[α]D (c = 0.91, $CHCl_3$) = −33.3

Compound S-3

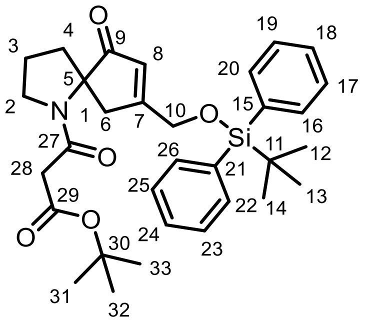

To a solution of **S-1** (0.10 mmol) in CH_2Cl_2 (2 mL) was added DIPEA (52 µL, 0.31 mmol) and the solution was stirred for 10 minutes. 3-(*Tert*-butoxy)-3-oxopropanoic acid (17 µL, 0.11 mmol), HATU (49 mg, 0.13 mmol) and DMAP (2.4 mg, 0.020 mmol) were added and the reaction mixture was stirred overnight at room temperature. Sat. aq. $NaHCO_3$ (2 mL) was added and the biphasic mixture was diluted with a further portion of CH_2Cl_2 (10 mL) and H_2O (10 mL). The layers were separated and the aqueous phase was extracted with CH_2Cl_2 (2 x 20 mL). The combined organics were dried with Na_2SO_4, filtered and concentrated under reduced pressure. The crude residue was purified by FCC (2:1 to 1:1 pentane:EtOAc) to yield the title compound as an off-white solid (44 mg, 81%).

^{1}H NMR (400 MHz, $CDCl_3$) δ 7.69 – 7.62 (m, 4H), 7.47 – 7.35 (m, 6H), 6.41 (p, *J* = 1.8 Hz, 1H), 4.51 (ddt, *J* = 17.6, 1.8, 0.8 Hz, 1H), 4.35 (ddt, *J* = 17.7, 1.9, 1.0 Hz, 1H), 3.66 – 3.58 (m, 2H), 3.34 (d, *J* = 15.5 Hz, 1H), 3.24 (d, *J* = 15.5 Hz, 1H), 3.10 – 3.00 (m, 1H), 2.32 (dd, *J* = 17.2, 1.3 Hz, 1H), 2.15 – 2.01 (m, 2H), 2.01 – 1.86 (m, 1H), 1.72 (ddd, *J* = 11.4, 5.9, 2.5 Hz, 1H), 1.46 (s, 9H), 1.06 (s, 9H).

^{13}C NMR (101 MHz, $CDCl_3$) δ 205.8, 175.4, 166.6, 164.1, 135.6, 135.6, 133.0, 132.8, 130.0, 130.0, 128.0, 127.9, 126.3, 82.1, 69.8, 64.0, 49.0, 43.8, 40.9, 38.5, 28.1, 26.8, 24.5, 19.3.

MP 130 °C

IR (thin film) ν_{max}/cm^{-1} 2931, 1717 (br), 1652, 1143

HRMS (ESI) mass calculated for [M+H]+ ($C_{32}H_{42}O_5NSi$) requires *m/z* 548.2827, found *m/z* 548.2831

Compound S-4

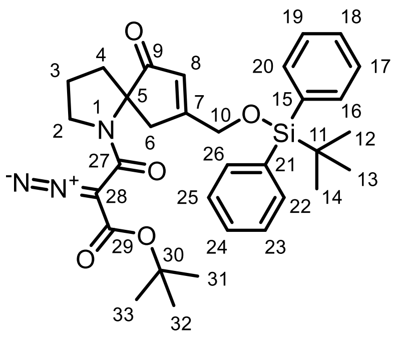

To a solution of **S-2** (15 mg, 0.027 mmol) in MeCN (270 µL), cooled to 0 °C, was added *p*-ABSA (7.8 mg, 0.033 mmol) followed by dropwise addition of Et_3N (4.6 µL, 0.033 mmol). The solution was stirred at 0 °C for 1 hour and then stirred at room temperature overnight. The volatiles were removed with a stream of N_2 and CH_2Cl_2 (2 mL) was added. The resulting suspension was filtered and the filtrate was concentrated under reduced pressure. The crude residue was purified by FCC (4:1 to 1:1 pentane:EtOAc) and then PTLC (3:1 hexane:EtOAc) to yield the title compound as an amorphous white solid (3.4 mg, 22%).

^{1}H NMR (600 MHz, $CDCl_3$) δ 7.65 (dt, *J* = 8.1, 1.6 Hz, 4H), 7.47 – 7.36 (m, 6H), 6.34 (p, *J* = 1.7 Hz, 1H), 4.50 (d, *J* = 17.8 Hz, 1H), 4.40 – 4.34 (ddd, J = 18.7, 2.3, 1.0 Hz, 1H), 3.81 (ddd, *J* = 10.4, 8.4, 6.5 Hz, 1H), 3.62 (ddd, *J* = 11.1, 7.3, 4.3 Hz, 1H), 3.16 – 3.10 (m, 1H), 2.38 – 2.32 (m, 1H), 2.19 (app. dq, *J* = 16.9, 7.8 Hz, 1H), 2.11 – 2.06 (m, 1H), 1.90 – 1.83 (m, 1H), 1.80 (ddd, *J* = 12.3, 9.2, 6.2 Hz, 1H), 1.49 (s, 9H), 1.07 (s, 9H).

^{13}C NMR (151 MHz, $CDCl_3$) δ 206.2, 175.6, 161.1, 160.0, 135.7, 135.7, 133.1, 132.9, 130.1, 130.1, 128.0, 128.0, 125.2, 82.7, 70.7, 68.1, 64.0, 50.3, 41.1, 37.5, 28.4, 26.9, 24.0, 19.4.

IR (thin film) ν_{max}/cm^{-1} 3133, 3047, 1715, 1402, 1112

HRMS (ESI) mass calculated for [M+H]+ ($C_{32}H_{40}O_5N_3Si$) requires *m/z* 574.2732, found *m/z* 574.2733

Compound S-5

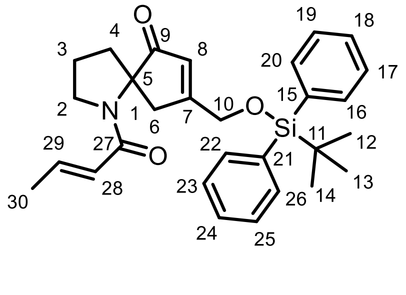

Following General Procedure 1 with work-up 3, compound **11** (50 mg, 0.10 mmol) was treated with TFA and the resulting crude amine hydrochloride salt was dissolved in CH_2Cl_2 (2 mL). General Procedure 3

was followed, employing but-2-enoyl chloride (11 µL, 0.11 mmol) as the coupling partner, DIPEA (87 µL, 0.50 mmol) as the base and a 4-hour reaction time. The crude residue was purified by FCC (1:1 pentane:EtOAc) to yield the title compound as a colourless oil (33 mg, 70%).

^{1}H NMR (400 MHz, $CDCl_3$) δ 7.72 – 7.59 (m, 4H), 7.49 – 7.34 (m, 6H), 6.89 (dq, *J* = 15.0, 6.9 Hz, 1H), 6.43 (dt, *J* = 3.2, 1.6 Hz, 1H), 6.11 (dq, *J* = 15.0, 1.6 Hz, 1H), 4.52 (ddt, *J* = 17.7, 1.8, 0.8 Hz, 1H), 4.35 (ddt, *J* = 17.7, 1.9, 1.0 Hz, 1H), 3.81 – 3.62 (m, 2H), 3.09 – 3.00 (m, 1H), 2.37 – 2.27 (m, 1H), 2.16 – 2.08 (m, 1H), 2.08 – 1.99 (m, 1H), 1.99 – 1.89 (m, 1H), 1.86 (dd, *J* = 6.9, 1.7 Hz, 3H), 1.72 (ddd, J = 11.4, 5.7, 3.0 Hz, 1H), 1.07 (s, 9H).

^{13}C NMR (101 MHz, $CDCl_3$) δ 206.2, 175.1, 164.0, 142.3, 135.6, 135.6, 133.1, 132.9, 130.0, 130.0, 128.0, 128.0, 126.3, 122.6, 69.9, 64.0, 48.3, 41.3, 38.2, 26.8, 24.5, 19.4, 18.3.

IR (thin film) ν_{max}/cm^{-1} 2931, 1716, 1664, 1141

HRMS (ESI) mass calculated for [M+H]+ ($C_{29}H_{36}O_3NSi$) requires *m/z* 474.2459, found *m/z* 474.2458

Compound S-6

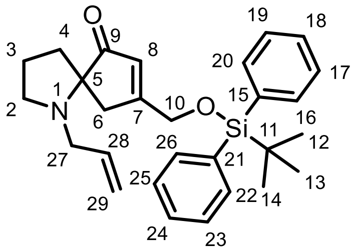

To a solution of **S-1** (0.10 mmol) in CH_2Cl_2 (2 mL) was added DIPEA (34 µL, 0.20 mmol) and the solution was stirred for 10 minutes. Allyl bromide (10 µL, 0.12 mmol) was added and the solution was stirred overnight. The reaction mixture was treated with sat. aq. $NaHCO_3$ (2 mL) and the biphasic mixture was diluted with a further portion of CH_2Cl_2 (10 mL) and H_2O (10 mL). The layers were separated and the aqueous phase was extracted with CH_2Cl_2 (2 x 20 mL). The combined organics were dried with Na_2SO_4, filtered and concentrated under reduced pressure. Crude ^{1}H NMR analysis confirmed low conversion to the allylated product **S-6** therefore the crude residue was treated with DMF (1.0 mL), K_2CO_3 (15 mg, 0.11 mmol) and allyl bromide (18 µL, 0.2 mmol). The reaction mixture was stirred for 5 hours at room temperature. Sat. aq. K_2CO_3 (2 mL) was added and the aqueous phase was extracted with CH_2Cl_2 (3 x 2 mL). The combined organics were dried with Na_2SO_4, filtered and concentrated under reduced pressure. The crude residue was purified by FCC (2:1 to 1:1 pentane:EtOAc) to yield the title compound as a colourless oil (18 mg, 40%).

^{1}**H NMR** (400 MHz, $CDCl_3$) δ 7.65 (app. dq, *J* = 6.5, 1.6 Hz, 4H), 7.45 – 7.34 (m, 6H), 6.23 (p, *J* = 1.8 Hz, 1H), 5.82 (dddd, *J* = 17.1, 10.0, 7.1, 6.0 Hz, 1H), 5.14 (dq, *J* = 17.0, 1.6 Hz, 1H), 5.02 (ddd, *J* = 10.0, 2.0, 1.1 Hz, 1H), 4.43 (td, *J* = 2.0, 1.0 Hz, 2H), 3.08 – 2.92 (m, 3H), 2.88 (td, *J* = 8.6, 3.8 Hz, 1H), 2.59 (d, *J* = 17.9 Hz, 1H), 2.39 (dd, *J* = 18.6, 1.6 Hz, 1H), 2.11 – 1.98 (m, 2H), 1.89 – 1.75 (m, 2H), 1.08 (s, 9H).

^{13}**C NMR** (101 MHz, $CDCl_3$) δ 211.7, 178.1, 136.5, 135.6, 132.9, 130.1, 128.0, 127.2, 116.9, 71.7, 64.2, 53.0, 52.1, 39.0, 37.3, 26.9, 21.9, 19.4.

IR (thin film) ν_{max}/cm^{-1} 2931, 1699, 1139

HRMS (ESI) mass calculated for [M+H]+ ($C_{28}H_{36}O_2NSi$) requires *m/z* 446.2510, found *m/z* 446.2528

Compound 25

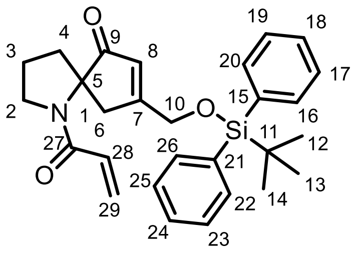

To a solution of **S-1** (0.10 mmol) in CH_2Cl_2 (2 mL) was added DIPEA (86 µL, 0.50 mmol) and the solution was stirred for 10 minutes before cooling to 0 °C. General Procedure 3 was followed, employing acryloyl chloride (16 µL, 0.2 mmol), DIPEA as the base and a 2.5-hour reaction time. The crude residue was purified by FCC (1:1 pentane:EtOAc) to yield the title compound as a colourless oil (32 mg, 71%).

^{1}**H NMR** (400 MHz, $CDCl_3$) δ 7.70 – 7.62 (m, 4H), 7.40 (m, 6H), 6.47 – 6.31 (m, 3H), 5.67 (dd, *J* = 9.8, 2.4 Hz, 1H), 4.57 – 4.45 (m, 1H), 4.36 (ddt, *J* = 17.7, 1.9, 1.0 Hz, 1H), 3.73 (ddd, *J* = 8.5, 4.8, 3.0 Hz, 2H), 3.11 – 3.01 (m, 1H), 2.34 (dd, *J* = 17.3, 1.4 Hz, 1H), 2.20 – 2.09 (m, 1H), 2.09 – 2.01 (m, 1H), 2.00 – 1.88 (m, 1H), 1.73 (ddd, *J* = 11.6, 5.8, 3.1 Hz, 1H), 1.07 (s, 9H).

^{13}**C NMR** (101 MHz, $CDCl_3$) δ 206.0, 175.3, 163.5, 135.6, 135.6, 133.0, 132.8, 130.0, 130.0, 128.6, 128.2, 128.0, 127.9, 126.3, 69.9, 64.0, 48.4 , 41.2, 38.1, 26.8, 24.5, 19.3.

IR (thin film) ν_{max}/cm^{-1} 2931, 1715, 1428, 1141

HRMS (ESI) mass calculated for [M+H]+ ($C_{28}H_{34}O_3NSi$) requires *m/z* 460.2302, found *m/z* 460.2303

Compound S-7

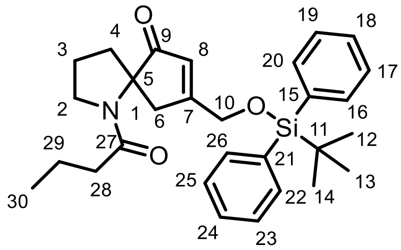

To a solution of **S-1** (0.10 mmol) in CH_2Cl_2 (2 mL) was added DIPEA (86 µL, 0.50 mmol) and the solution was stirred for 10 minutes before cooling to 0 °C. General Procedure 3 was followed, employing butyryl chloride (21 µL, 0.20 mmol), DIPEA (52 µL, 0.30 mmol) as the base and a 2-hour reaction time. The crude residue was purified by FCC (3:1 to 1:1 pentane:EtOAc) to yield the title compound as a colourless oil (46 mg, 97%).

^{1}H NMR (400 MHz, $CDCl_3$) δ 7.66 (app. ddt, *J* = 6.8, 1.7, 0.9 Hz, 4H), 7.49 – 7.34 (m, 6H), 6.42 (dt, *J* = 3.3, 1.7 Hz, 1H), 4.51 (ddt, *J* = 17.7, 1.9, 0.8 Hz, 1H), 4.34 (ddt, *J* = 17.6, 1.9, 1.0 Hz, 1H), 3.66 – 3.55 (m, 2H), 2.98 (br. d, *J* = 17.3, 1H), 2.37 – 2.17 (m, 3H), 2.14 – 1.97 (m, 2H), 1.97 – 1.87 (m, 1H), 1.73 – 1.57 (m, 4H), 1.06 (s, 9H), 0.94 (t, *J* = 7.4 Hz, 3H).

^{13}C NMR (101 MHz, $CDCl_3$) δ 206.5, 175.1, 171.0, 135.7, 135.6, 133.1, 132.9, 130.1, 130.0, 128.0, 128.0, 126.4, 69.7, 64.0, 48.5, 41.4, 38.4, 36.6, 26.8, 24.6, 19.4, 18.0, 14.0.

IR (thin film) ν_{max}/cm^{-1} 2961, 1716, 1645, 1141

HRMS (ESI) mass calculated for [M+H]+ ($C_{29}H_{38}O_3NSi$) requires *m/z* 476.2615, found *m/z* 476.2610

Compound S-8

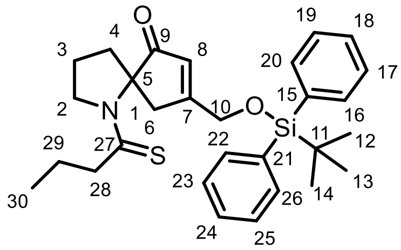

To a solution of **S-7** (20 mg, 0.042 mmol) in PhMe (420 µL) was added Lawesson's reagent (9.4 mg, 0.023 mmol). The solution was heated to 100 °C for 16 hours and then the solvent was removed under reduced pressure. The crude residue was purified by PTLC (2:1 hexane:EtOAc) to yield the title compound as a colourless oil (0.5 mg, 2%), as well as **S-7** (starting material) (6.0 mg, 30%). Further analytic data was obtained from purification of additional runs.

^{1}H NMR (600 MHz, $CDCl_3$) δ 7.66 (ddt, *J* = 8.0, 6.6, 1.5 Hz, 4H), 7.50 – 7.36 (m, 6H), 6.47 – 6.44 (m, 1H), 4.46 (d, *J* = 17.8 Hz, 1H), 4.38 (d, *J* = 16.8 Hz, 1H), 3.86 – 3.81 (m, 1H), 3.75 (td, *J* = 10.9, 6.1 Hz, 1H), 3.61 – 3.55 (m, 1H), 2.69 – 2.57 (m, 2H), 2.29 (d, *J* = 17.1 Hz, 1H), 2.17 – 2.09 (m, 2H), 2.03 – 1.93 (m, 1H), 1.86 – 1.72 (m, 3H), 1.07 (s, 9H), 0.99 (t, *J* = 7.4 Hz, 3H).

^{13}C NMR (151 MHz, $CDCl_3$) δ 202.4, 201.4, 172.2, 135.6, 135.6, 133.2, 132.8, 130.1, 130.1, 128.1, 128.0, 127.9, 74.8, 63.9, 53.1, 46.4, 39.0, 38.7, 26.8, 24.1, 22.6, 19.4, 13.8.

IR (thin film) ν_{max}/cm^{-1} 2960, 2930, 2857, 1713, 1139, 1112, 1084

HRMS (ESI) mass calculated for [M+H]+ ($C_{29}H_{38}O_2NSiS$) requires *m/z* 492.2387, found *m/z* 492.2387

Compound 12

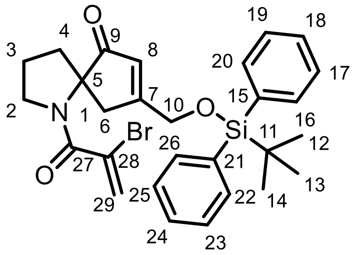

To a solution of compound **11** (0.10 mmol), General Procedure 3 was followed, employing 2-bromo-acryloyl chloride (0.2 M in CH_2Cl_2, 1.0 mL, assumed 0.20 mmol, prepared following General Procedure 2), DIPEA (86 μL, 0.50 mmol) as the base. After 2 hours, a further portion of 2-bromo-acryloyl chloride (0.2 M in CH_2Cl_2, 1.0 mL, 0.20 mmol) was added and the solution was stirred for a further 2 hours. The crude residue was purified by FCC (3:1 to 1:1 pentane:EtOAc) to yield the title compound as an off-white solid (33 mg, 61%).

^{1}H NMR (400 MHz, $CDCl_3$) δ 7.71 – 7.61 (m, 4H), 7.51 – 7.35 (m, 6H), 6.41 (p, *J* = 1.8 Hz, 1H), 6.13 (d, *J* = 2.5 Hz, 1H), 5.87 (d, *J* = 2.5 Hz, 1H), 4.52 (ddt, *J* = 17.8, 1.8, 0.8 Hz, 1H,), 4.38 (ddt, *J* = 17.8, 1.9, 1.0 Hz, 1H), 3.79 – 3.62 (m, 2H), 3.08 (ddt, *J* = 17.5, 2.2, 1.2 Hz, 1H), 2.35 (dd, *J* = 17.5, 1.4 Hz, 1H), 2.19 – 2.02 (m, 2H), 1.99 – 1.84 (m, 1H), 1.83 – 1.73 (m, 1H), 1.07 (s, 9H).

^{13}C NMR (101 MHz, $CDCl_3$) δ 205.4, 175.7, 163.6, 135.6, 133.0, 132.8, 130.1, 130.1, 128.1, 128.0, 126.0, 122.3, 122.2, 69.9, 64.0, 50.5, 40.7, 38.2, 26.8, 24.5, 19.4.

MP 122-126 °C

IR (thin film) ν_{max}/cm^{-1} 2858, 1716, 1643, 1427, 1141

HRMS (ESI) mass calculated for [M+H]+ ($C_{28}H_{33}O_3NBrSi$) requires *m/z* 538.1408 & 540.1389, found *m/z* 538.1410 & 540.1387

Compound 13

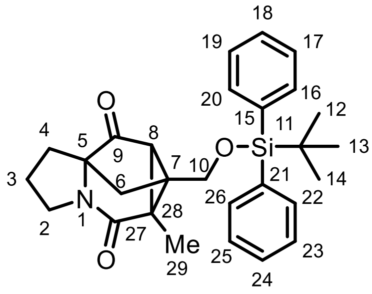

A microwave vial was charged with compound **12** (25 mg, 0.047 mmol), $Pd(OAc)_2$ (0.4 mg, 2 μmol) and $NaHCO_2$ (4.0 mg, 0.059 mmol). The vial was sealed with a crimped cap then evacuated and back-filled N_2 and this was repeated two times. A solution of TBACl (12.9 mg, 0.047 mmol) in DMF (1.0 mL) was added, followed by addition of Et_3N (16.3 μL, 0.12 mmol). The resulting mixture was heated to 80 °C for 18 hours before it was cooled to room temperature and EtOAc (10 mL) was added. The organic layer was washed with brine, dried with Na_2SO_4, filtered and concentrated under reduced pressure. The crude residue was purified by FCC (1:1 pentane:EtOAc) to yield the title compound as a white solid (15.3 mg, 71%).

^{1}H NMR (500 MHz, $CDCl_3$) δ 7.69 – 7.59 (m, 4H), 7.51 – 7.35 (m, 6H), 3.94 (d, *J* = 11.5 Hz, 1H), 3.87 (d, *J* = 11.5 Hz, 1H), 3.46 (td, *J* = 6.8, 1.3 Hz, 2H), 2.40 (dt, *J* = 13.3, 6.7 Hz, 1H), 2.29 (d, *J* = 11.9 Hz, 1H), 1.94 (d, *J* = 11.9 Hz, 1H), 1.87 (p, *J* = 7.0 Hz, 2H), 1.74 (dt, *J* = 13.1, 7.6 Hz, 1H), 1.56 (s, 1H), 1.33 (s, 3H), 1.05 (s, 9H).

^{13}C NMR (126 MHz, $CDCl_3$) δ 204.8, 167.6, 135.7, 135.7, 133.0, 133.0, 130.2, 130.1, 128.0, 128.0, 65.2, 62.6, 45.0, 40.9, 38.7, 36.5, 34.7, 27.0, 26.9, 23.8, 19.4, 13.1.

MP 94-100 °C

IR (thin film) ν_{max}/cm^{-1} 2932 1749, 1667, 1113

HRMS (ESI) mass calculated for [M+H]+ ($C_{28}H_{34}O_3NSi$) requires *m/z* 460.2302, found *m/z* 460.2301

Compound 14

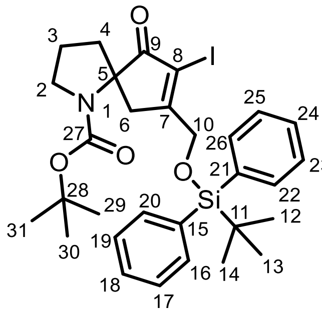

To a solution of compound **11** (4.5 g, 8.9 mmol) in CH_2Cl_2 (89 mL) was added pyridine (1.8 mL, 22 mmol) and I_2 (7.9 g, 31 mmol). The resulting mixture was covered with aluminium foil and stirred overnight at room temperature. Sat. aq. $Na_2S_2O_3$ (50 mL) and sat. aq. $NaHCO_3$ (50 mL) were added. The layers were separated and the aqueous phase was extracted with CH_2Cl_2 (2 x 100 mL). The combined organics were dried with Na_2SO_4, filtered and the solvent was removed under reduced pressure. The crude residue was purified by FCC (4:1 to 2:1 pentane:EtOAc) to yield the title compound as an off-white solid (4.7 g, 83%).

^{1}H NMR (600 MHz, $CDCl_3$, mixture of rotamers) δ 7.66 (m, 4H), 7.49 – 7.45 (m, 2H), 7.42 (m, 4H), 4.64 (s, $2H_{min}$), 4.60 (s, $2H_{maj}$), 3.66 – 3.56 (m, $2H_{maj}+1H_{min}$), 3.54 (m, $1H_{min}$), 3.30 (d, *J* = 18.0 Hz, $1H_{min}$), 3.18 – 3.11 (m, $1H_{maj}$), 2.88 (br. d, *J* = 18.3, 1H), 2.08 – 1.96 (m, 2H), 1.92 – 1.81 (m, 1H), 1.78 – 1.71 (m, $1H_{maj}$), 1.70 – 1.66 (m, $1H_{min}$), 1.42 (s, $9H_{min}$), 1.28 (s, $9H_{maj}$), 1.10 (s, $9H_{maj}$), 1.08 (s, $9H_{min}$).

^{13}C NMR (151 MHz, $CDCl_3$, mixture of rotamers) δ 202.4, 202.3, 176.9, 176.9, 153.3, 152.9, 135.7, 135.6, 132.9, 132.7, 132.6, 132.5, 130.3, 130.2, 130.2, 128.1, 128.1, 128.1, 128.1, 96.2, 95.8, 80.7, 80.1, 66.5, 66.3, 66.3, 65.8, 48.2, 47.9, 46.4, 44.7, 40.2, 39.3, 28.6, 28.4, 26.9, 23.9, 23.3, 19.4.

MP 38 °C

IR (thin film) ν_{max}/cm^{-1} 1730, 1698, 1390, 1114, 703

HRMS (ESI) mass calculated for [M+Na]+ ($C_{30}H_{38}O_4NSiINa$) requires *m/z* 654.1507, found *m/z* 654.1507

<u>Compound 15</u>

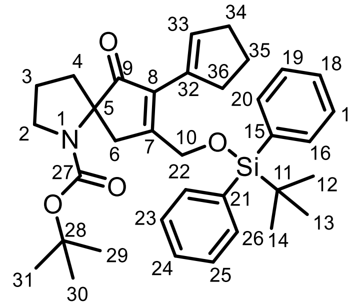

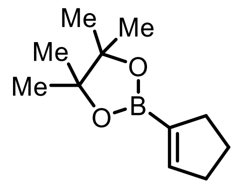

S-9

Following a modified literature procedure,[5] to a round-bottomed flask was added compound **14** (300 mg, 0.471 mmol), $AsPh_3$ (14.0 mg, 0.0457 mmol) and Ag_2O (169 mg, 0.728 mmol). The flask was evacuated and back-filled with N_2 three times. 1,4-Dioxane (48 ml) was added, followed by a solution of 2-(cyclopent-1-en-1-yl)-4,4,5,5-tetramethyl-1,3,2-dioxaborolane **S-9** (134 mg, 0.69 mmol) in 1,4-dioxane (10 mL) and H_2O (5.8 mL). The solution was sparge with argon gas for 5 minutes and $Pd(PhCN)_2Cl_2$ (8.8 mg, 0.0231 mmol) was added. The resulting mixture was stirred at room temperature for 5 hours before it was passed through a Celite® plug and the volatiles were removed. The crude residue was purified by FCC (5:1 to 3:1 pentane:EtOAc) to yield the title compound as a colourless oil (250 mg, 93%).

^{1}H NMR (600 MHz, $CDCl_3$, mixture of rotamers) δ 7.66 (m, 4H), 7.48 – 7.43 (m, 2H), 7.43 – 7.36 (m, 4H), 6.01 (t, J = 2.3 Hz, $1H_{min}$), 5.93 (p, J = 2.2 Hz, $1H_{maj}$), 4.77 (d, J = 15.6 Hz, $1H_{min}$), 4.73 – 4.62 (m, 2H), 3.66 – 3.50 (m, 2H), 3.20 (d, J = 18.2 Hz, $1H_{min}$), 3.09 (d, J = 18.6 Hz, $1H_{maj}$), 2.81 (d, J = 18.3 Hz, $1H_{min}$), 2.76 (d, J = 18.6 Hz, $1H_{maj}$), 2.49 – 2.37 (m, 2H), 2.37 – 2.31 (m, $2H_{maj}$), 2.31 – 2.26 (m, $2H_{min}$), 2.07 – 1.95 (m, 2H), 1.92 – 1.83 (m, 1H), 1.80 (pd, J = 7.5, 1.4 Hz, 1H), 1.76 – 1.66 (m, 1H), 1.43 (s, $9H_{min}$), 1.29 (s, $9H_{maj}$), 1.09 (s, $9H_{maj}$), 1.07 (s, $9H_{min}$).

^{13}C NMR (151 MHz, $CDCl_3$, mixture of rotamers) δ 206.7, 206.6, 166.5, 166.4, 153.3, 153.3, 135.7, 135.7, 134.3, 134.3, 133.3, 133.1, 133.1, 133.0, 132.9, 130.1, 130.0, 130.0, 128.0, 128.0, 128.0, 127.9, 80.1, 79.6, 68.2, 67.7, 62.4, 62.4, 48.3, 48.1, 42.7, 41.1, 40.3, 39.5, 34.7, 34.6, 33.0, 32.9, 28.6, 28.4, 26.9, 24.0, 23.5, 23.4, 23.3, 19.4.

IR (thin film) ν_{max}/cm^{-1} 2959, 1717, 1696, 1391, 1112

HRMS (ESI) mass calculated for [M+Na]+ ($C_{35}H_{45}O_4NSiNa$) requires m/z 594.3010, found m/z 594.3031

Compound 16

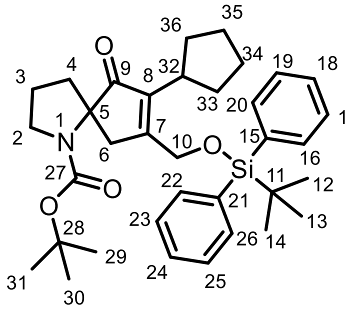

To a solution of compound **15** (40 mg, 0.070 mmol) in EtOH (4.7 mL) was added Rh/C (20 mg, 5 wt%). The resulting suspension was sparged with H_2 for 5 minutes and stirred for 2 hours. A further portion of Rh/C (22 mg) was added followed by a further 5 minutes sparging with H_2. This was repeated two further times, roughly separated by 2 hours each. The reaction mixture was passed through a Celite® plug and concentrated under reduced pressure. The crude residue was purified by FCC (4:1 pentane:EtOAc) to yield the title compound as a colourless oil (28.9 mg, 72%).

^{1}H NMR (400 MHz, $CDCl_3$, mixture of rotamers) δ 7.67 (m, 4H), 7.48 – 7.36 (m, 6H), 4.68 – 4.48 (m, 2H), 3.65 – 3.45 (m, 2H), 3.04 (d, *J* = 18.0 Hz, 1H), 2.70 – 2.55 (m, 2H), 2.02 – 1.92 (m, 2H), 1.91 – 1.43 (m, 10H), 1.32 (m, 9H), 1.07 (m, 9H).

^{13}C NMR (101 MHz, $CDCl_3$, mixture of rotamers) δ 207.5, 207.5, 165.6, 165.4, 153.4, 139.4 135.7, 135.7, 135.7, 135.6, 133.3, 133.1, 133.0, 132.9, 130.1, 130.0, 128.0, 127.9, 127.9, 80.0, 79.4, 68.0, 67.9, 61.8, 61.7, 48.4, 48.4, 42.2, 40.8, 40.5, 39.3, 35.8, 35.7, 30.9, 30.8, 28.6, 28.5, 26.9, 26.3, 26.2, 23.9, 23.3, 19.4.

IR (thin film) ν_{max}/cm^{-1} 2955, 1712, 1697, 1391

HRMS (ESI) mass calculated for [M+Na]+ ($C_{35}H_{47}O_4NSiNa$) requires *m/z* 596.3167, found *m/z* 596.3165

<u>Compound 17</u>

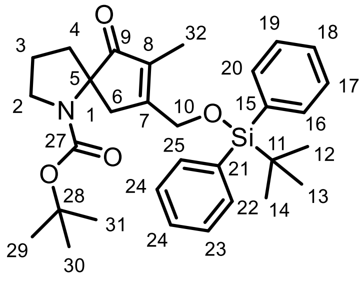

Following a modified literature procedure,[6] to a solution of compound **14** (100 mg, 0.158 mmol) in 1,4-dioxane (5.3 mL) was added Cs_2CO_3 (191 mg, 0.586 mmol), $Me_3B_3O_3$ (66.0 μL, 0.474 mmol) then $PdCl_2$(dppf) (4.6 mg, 6.3 μmol). The mixture was stirred at reflux for 5 hours then cooled to room

temperature and H_2O (10 mL) and EtOAc (20 mL) were added. The layers were separated and the aqueous layer was extracted with EtOAc (2 x 20 mL). The combined organics were dried with Na_2SO_4, filtered and concentrated under reduced pressure. The crude residue was purified by FCC (5:1 to 4:1 pentane:EtOAc) to yield the title compound as a colourless oil (63.7 mg, 78%).

^{1}H NMR (600 MHz, $CDCl_3$, mixture of rotamers) δ 7.70 – 7.63 (m, 4H), 7.48 – 7.36 (m, 6H), 4.63 – 4.48 (m, 2H), 3.65 – 3.59 (m, 1H), 3.59 – 3.50 (m, 1H), 3.04 (d, *J* = 17.5 Hz, $1H_{min}$), 2.93 (dd, *J* = 17.7, 2.6 Hz, $1H_{maj}$), 2.68 – 2.57 (m, 1H), 2.05 – 1.93 (m, 2H), 1.91 – 1.81 (m, 1H), 1.72 – 1.65 (m, $1H_{maj}$), 1.64 – 1.58 (m, 3H), 1.42 (s, $9H_{min}$), 1.28 (s, $9H_{maj}$), 1.08 (s, $9H_{maj}$), 1.06 (s, $9H_{min}$).

^{13}C NMR (151 MHz, $CDCl_3$, mixture of rotamers) δ 208.1, 208.0, 166.2, 166.0, 153.3, 153.2, 135.7, 135.7, 135.6, 133.2, 133.2, 133.0, 133.0, 133.0, 132.9, 130.2 , 130.0, 128.0, 128.0, 128.0, 80.0, 79.7, 67.9, 67.4, 62.1, 62.0, 48.2, 48.0, 42.8, 41.3, 40.2, 39.4, 28.6, 28.3, 26.9, 24.1, 23.5, 19.4, 8.6, 8.5.

IR (thin film) ν_{max}/cm^{-1} 2931, 1714, 1696, 1392

HRMS (ESI) mass calculated for [M+Na]+ ($C_{31}H_{41}O_4NSiNa$) requires *m/z* 542.2697, found *m/z* 542.2690

Compound 18

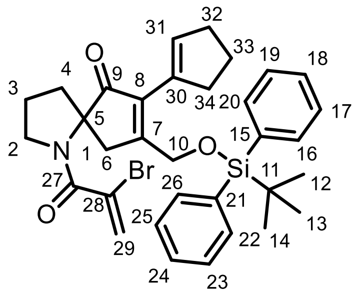

Following General Procedure 1 with work-up 3, compound **15** (125 mg, 0.220 mmol) was reacted, following General Procedure 3, with 2-bromo-acryloyl chloride (assumed 0.44 mmol, prepared following General Procedure 2). Further portions of DIPEA (110 μL) and acid chloride solution (0.59 mL, 0.44 mmol) were added after 2 hours and the reaction was stirred for a further 2.5 hours before work-up in the usual manner. The crude residue was purified by FCC (3:1 to 2:1 pentane:EtOAc) to yield the title compound as a colourless oil (66.2 mg, 50%).

^{1}H NMR (400 MHz, $CDCl_3$) δ 7.73 – 7.58 (m, 4H), 7.52 – 7.32 (m, 6H), 6.12 (d, *J* = 2.5 Hz, 1H), 5.97 (p, *J* = 2.2 Hz, 1H), 5.86 (d, *J* = 2.5 Hz, 1H), 4.78 (d, *J* = 15.5 Hz, 1H), 4.67 (d, *J* = 15.5 Hz, 1H), 3.73 (m, 2H), 3.23 (d, *J* = 18.3 Hz, 1H), 2.83 (d, *J* = 18.3 Hz, 1H), 2.52 – 2.41 (m, 1H), 2.32 (m, 3H), 2.19 – 1.92 (m, 3H), 1.81 – 1.70 (m, 3H), 1.07 (s, 9H).

^{13}**C NMR** (101 MHz, $CDCl_3$) δ 204.8, 166.2, 163.3, 135.7, 135.6, 134.1, 133.3, 133.2, 132.9, 130.0, 130.0, 128.0, 127.9, 122.4, 122.0, 69.1, 62.3, 50.5, 40.5, 38.4, 34.5, 32.9, 26.9, 24.6, 23.3, 19.4.

IR (thin film) ν_{max}/cm^{-1} 2928, 1714, 1642, 1427, 1111

HRMS (ESI) mass calculated for [M+Na]+ ($C_{33}H_{38}O_3NBrSiNa$) requires *m/z* 626.1697, found *m/z* 626.1710

Compound 19

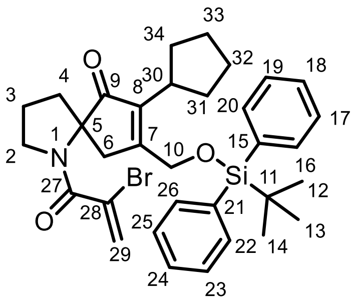

Following General Procedure 1 with work-up 2, compound **16** (50.5 mg, 0.088 mmol) was reacted, following General Procedure 3, with 2-bromo-acryloyl chloride (500 µL, 0.34 M in CH_2Cl_2, assumed 0.175 mmol, prepared via General Procedure 2). The reaction was monitored by TLC and a further portion of DIPEA (30 µL, 0.173 mmol) and acid chloride solution (250 µL) was added after 2 hours. The reaction was stirred for a further 2 hours before work-up. The crude residue was purified by FCC (3:1 to 2:1 pentane:EtOAc) to yield the title compound as a colourless oil (30.4 mg, 57%).

^{1}**H NMR** (400 MHz, $CDCl_3$) δ 7.67 (m, 4H), 7.42 (m, 6H), 6.11 (d, *J* = 2.5 Hz, 1H), 5.86 (d, *J* = 2.5 Hz, 1H), 4.66 (dt, *J* = 15.0, 1.2 Hz, 1H), 4.54 (dt, *J* = 14.9, 1.3 Hz, 1H), 3.78 – 3.62 (m, 2H), 3.09 (dt, *J* = 17.6, 1.5 Hz, 1H), 2.75 – 2.55 (m, 2H), 2.17 – 1.90 (m, 3H), 1.80 – 1.50 (m, 7H), 1.45 (tt, *J* = 7.3, 3.8 Hz, 2H), 1.07 (s, 9H).

^{13}**C NMR** (101 MHz, $CDCl_3$) δ 205.5, 165.2, 163.2, 139.8, 135.7, 135.6, 133.2, 132.9, 130.1, 130.0, 128.0, 128.0, 122.4, 121.9, 69.1, 61.7, 50.5, 40.3, 38.3, 35.7, 30.6, 30.4, 26.9, 26.5, 26.4, 24.7, 19.4.

IR (thin film) ν_{max}/cm^{-1} 2954, 1711, 1644, 1428

HRMS (ESI) mass calculated for [M+H]+ ($C_{33}H_{41}O_3NBrSi$) requires *m/z* 606.2034, found *m/z* 606.2026

Compound 20

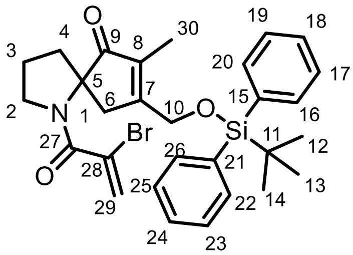

Following General Procedure 1 with work-up 3, compound **17** (60.0 mg, 0.116 mmol) was reacted, following General Procedure 3, with 2-bromo-acryloyl chloride (463 µL, 0.50 M in CH_2Cl_2, assumed 0.23 mmol, prepared via General Procedure 2). The crude residue was purified by FCC (3:1 to 2:1 pentane:EtOAc) to yield the title compound as a colourless oil (44.2 mg, 69%).

^{1}H NMR (400 MHz, $CDCl_3$) δ 7.71 – 7.63 (m, 4H), 7.50 – 7.35 (m, 6H), 6.11 (d, *J* = 2.5 Hz, 1H), 5.86 (d, *J* = 2.5 Hz, 1H), 4.62 (dq, *J* = 15.4, 1.1 Hz, 1H), 4.52 (dq, *J* = 15.5, 1.3 Hz, 1H), 3.80 – 3.64 (m, 2H), 3.15 – 3.04 (m, 1H), 2.70 – 2.60 (m, 1H), 2.19 – 1.90 (m, 2H), 1.75 (dt, *J* = 11.4, 5.1 Hz, 1H), 1.60 (p, *J* = 1.2 Hz, 3H), 1.07 (s, 9H).

^{13}C NMR (101 MHz, $CDCl_3$) δ 206.2, 165.7, 163.3, 135.7, 135.6, 133.1, 133.1, 132.9, 130.1, 130.1, 128.0, 128.0, 122.4, 122.0, 68.8, 62.0, 50.5, 40.6, 38.3, 26.9, 24.6, 19.4, 8.4.

IR (thin film) ν_{max}/cm^{-1} 2931, 1717, 1642, 1428, 1113

HRMS (ESI) mass calculated for [M+Na]+ ($C_{29}H_{34}O_3NBrSiNa$) requires *m/z* 574.1384, found *m/z* 574.1390

Compound S-10

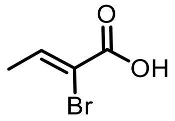

Following a literature procedure,[7] to a solution of methyl crotonate (2.10 mL, 19.7 mmol) in CH_2Cl_2 (25 mL), cooled to 0 °C, was added Br_2 (1.1 mL, 21.7 mmol) over 1 hour. The solution was stirred for a further 15 minutes at 0 °C then for 1 hour at room temperature. The volatiles were removed under reduced pressure (10 mbar, 35 °C). DMSO (57 mL) and H_2O (3 mL) was added and the resulting solution was heated at 85 °C for 16 hours. The mixture was cooled to room temperature and cold aq. $NaHCO_3$ (120 mL) was added. The aqueous layer was extracted with Et_2O (2 x 100 mL) and the combined organics were washed with brine (50 mL), dried with Na_2SO_4, filtered and concentrated under reduced pressure. THF (20 mL) was added followed by dropwise addition of $LiOH·H_2O$ (2.50 g, 60 mmol) in H_2O (20 mL). The

resulting solution was stirred at room temperature for 12 hours before the addition of 10% aq. $KHSO_4$ (150 mL). The aqueous layer was extracted with EtOAc (3 x 100 mL) and the combined organics were dried with Na_2SO_4, filtered and concentrated under reduced pressure. The resulting solid was washed with pentane and dried in vacuo to yield the title compound as a light pink-white solid (1.64 g, 51%). Data were in agreement with the literature.[7]

^{1}H NMR (400 MHz, $CDCl_3$) δ 11.99 (s, 1H), 7.55 (q, *J* = 6.9 Hz, 1H), 1.99 (d, *J* = 6.8 Hz, 3H).

^{13}C NMR (101 MHz, $CDCl_3$) δ 168.1, 144.8, 116.8, 18.3.

Compound 21

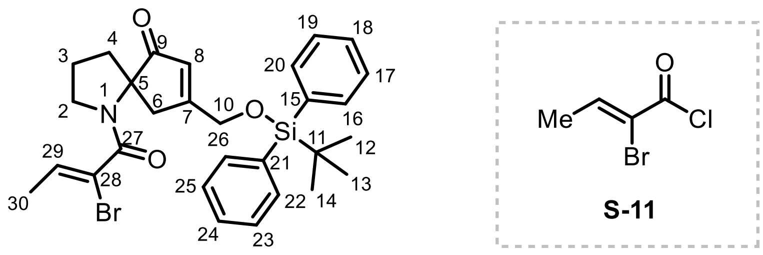

Following General Procedure 1 with work-up 3, compound **11** (100 mg, 0.20 mmol) was reacted, following General Procedure 3 with acid chloride **S-11** (600 μL, 0.65 M in CH_2Cl_2, assumed 0.39 mmol, prepared via General Procedure 2 from **S-10**). The crude residue was purified by FCC (2:1 to 1:1 pentane:EtOAc) to yield the title compound as a colourless oil (75.9 mg, 69%).

^{1}H NMR (400 MHz, $CDCl_3$) δ 7.71 – 7.62 (m, 4H), 7.49 – 7.34 (m, 6H), 6.44 – 6.34 (m, 2H), 4.56 – 4.47 (m, 1H), 4.44 – 4.33 (m, 1H), 3.73 – 3.60 (m, 2H), 3.15 – 3.05 (m, 1H), 2.33 (dd, *J* = 17.4, 1.6 Hz, 1H), 2.10 (m, 2H), 1.96 – 1.74 (m, 5H), 1.07 (s, 9H).

^{13}C NMR (101 MHz, $CDCl_3$) δ 205.7, 175.6, 164.3, 135.6, 133.0, 132.8, 131.3, 130.1, 130.0, 128.0, 128.0, 126.0, 118.0, 69.9, 64.0, 50.6, 40.7, 38.2, 26.8 , 24.4, 19.4, 16.6.

IR (thin film) ν_{max}/cm^{-1} 2931, 1716, 1627, 1408

HRMS (ESI) mass calculated for [M+H]+ ($C_{29}H_{35}O_3NBrSi$) requires *m/z* 552.1564 & 554.1546, found *m/z* 552.1567 & 554.1546

Compound 23

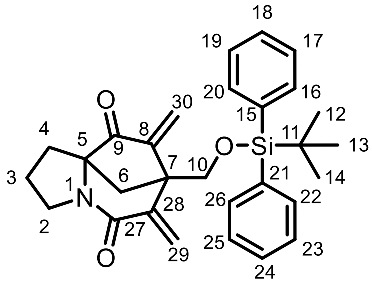

A microwave vial was charged with compound **20** (10 mg, 0.018 mmol), $Pd(OAc)_2$ (0.4 mg, 2 μmol). The vial was sealed with a crimped cap then evacuated and back-filled N_2 and this was repeated two times. A solution of TBACl (400 μL, 0.045 M in DMF, 0.018 mmol) was added, followed by addition of Et_3N (6.3 μL, 0.045 mmol). The resulting mixture was heated to 80 °C for 18 hours before it was cooled to room temperature and EtOAc (10 mL) was added. The organic layer was washed with brine, dried with Na_2SO_4, filtered and concentrated under reduced pressure. The crude residue was purified by PTLC (3:2 hexane:EtOAc) to yield the title compound as a white solid (1.8 mg, 21%).

^{1}H NMR (600 MHz, $CDCl_3$) δ 7.63 (app. ddt, *J* = 8.1, 2.5, 1.4 Hz, 4H), 7.53 – 7.43 (m, 2H), 7.43 – 7.37 (m, 4H), 6.27 (s, 1H), 6.15 (d, *J* = 1.3 Hz, 1H), 5.29 (d, *J* = 1.3 Hz, 1H), 5.25 (s, 1H), 4.13 (dd, *J* = 10.5, 1.3 Hz, 1H), 4.00 (dd, *J* = 10.6, 1.3 Hz, 1H), 3.69 (dt, *J* = 12.2, 8.3 Hz, 1H), 3.56 – 3.45 (m, 1H), 2.44 (ddd, *J* = 12.5, 7.4, 2.8 Hz, 1H), 2.24 – 2.15 (m, 2H), 2.05 – 1.96 (m, 2H), 1.91 (ddd, *J* = 12.4, 10.3, 8.0 Hz, 1H), 1.05 (s, 9H).

^{13}C NMR (151 MHz, $CDCl_3$) δ 198.2, 162.0, 145.5, 141.4, 135.9, 135.8, 133.0, 132.9, 130.2, 130.2, 128.0, 128.0, 120.3, 117.7, 68.3, 64.4, 49.1, 45.8, 40.2, 29.3, 27.1, 22.5, 19.5.

IR (thin film) ν_{max}/cm^{-1} 2930, 1738, 1662, 1618, 1113

HRMS (ESI) mass calculated for [M+H]+ ($C_{29}H_{34}O_3NSi$) requires *m*/*z* 472.2303, found *m*/*z* 472.2300

Compound 24

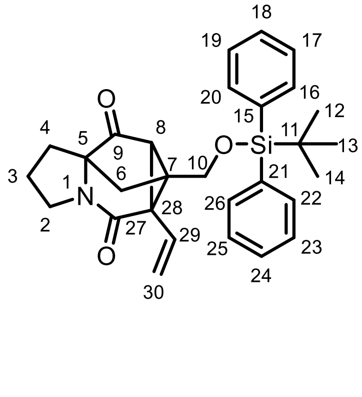

A microwave vial was charged with compound **21** (10 mg, 0.018 mmol), $Pd(OAc)_2$ (0.4 mg, 2 μmol), PPh_3 (1.6 mg, 7.2 μmol), Proton-sponge® (4.6 mg, 0.022 mmol), K_2CO_3 (6.6 mg, 0.048 mmol) then PhMe (360 μL). The reaction mixture was heated to 110 °C for 9 hours (200 μL additional PhMe was added at 5 hours). The mixture was cooled and passed through a silica plug. The solvent was removed and the crude residue was purified by PTLC (1:1 hexane:EtOAc) to yield the title compound as an amorphous white solid (1.4 mg, 17%).

^{1}H NMR (600 MHz, $CDCl_3$) δ 7.62 – 7.59 (m, 4H), 7.47 – 7.43 (m, 2H), 7.39 (app. tt, *J* = 6.7, 1.0 Hz, 4H), 6.22 (dd, *J* = 17.4, 10.8 Hz, 1H), 5.21 (dd, *J* = 10.8, 0.8 Hz, 1H), 4.82 (dd, *J* = 17.5, 0.9 Hz, 1H), 3.86 (d, *J* = 11.4 Hz, 1H), 3.75 (d, *J* = 11.4 Hz, 1H), 3.51 – 3.45 (m, 2H), 2.47 – 2.40 (m, 1H), 2.38 (d, *J* = 12.2 Hz, 1H), 2.06 – 2.00 (m, 2H), 1.94 – 1.84 (m, 2H), 1.79 (dt, *J* = 13.1, 7.6 Hz, 1H), 1.04 (s, 9H).

^{13}C NMR (151 MHz, $CDCl_3$) δ 204.1, 166.1, 135.8, 135.7, 133.1, 133.1, 130.2, 130.1, 129.2, 128.0, 128.0, 119.1, 65.2, 61.9, 45.0, 44.5, 42.5, 36.4, 30.6, 27.0, 26.9, 23.8, 19.4.

MP 44-46 °C

IR (thin film) ν_{max}/cm^{-1} 2930, 1751, 1668 1112

HRMS (ESI) mass calculated for [M+H]+ ($C_{29}H_{34}O_3NSi$) requires *m/z* 472.2302, found *m/z* 472.2304

Compound 4

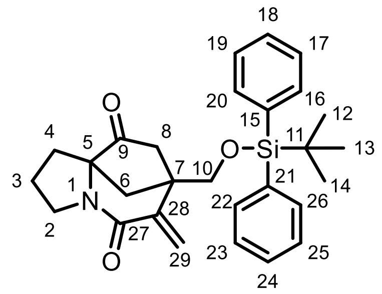

A microwave vial was charged with compound **12** (10 mg, 0.019 mmol), $Pd(OAc)_2$ (0.4 mg, 2 μmol), PPh_3 (1.0 mg, 4 μmol) and TBAI (7 mg, 0.019 mmol). The vial was sealed and charged with DMF (400 μL), TFA (3.6 μL, 0.049 mmol) and *n*-Bu_3N (20 μL, 0.086 mmol). The reaction mixture was heated to 60 °C and stirred overnight. The vial was cooled to room temperature and the reaction mixture was passed through a silica plug and the volatiles were removed under reduced pressure. The product was determined to be formed in 70% yield as determined by crude ^{1}H NMR using 1,2,4,5-tetramethylbenzene as internal standard.

Alternative procedure, with purification: A microwave vial was charged with compound **12** (10 mg, 0.019 mmol), $Pd(OAc)_2$ (0.4 mg, 2 μmol), adamantane carboxylic acid (6.8 mg, 0.038 mmol) and TBACl (5.2 mg, 0.019 mmol). The vial was sealed then evacuated and backfilled with N_2 three times. DMF (400 μL) was added followed by Et_3N (6.6 μL, 0.048 mmol). The reaction mixture was heated to 80 °C for 16 hours. The mixture was cooled to room temperature and worked up as above. The crude residue was purified by PTLC (1:1 hexane:EtOAc) to yield the title compound as a waxy solid (2.4 mg, 27%) and Compound **26** (3.0 mg, 34%).

Pure **4** was also obtained by PTLC (1:1 hexane:EtOAc) or by FCC (2:1 to 1:1 pentane:EtOAc) on combined crude mixtures from a number of optimisation runs and afforded the title compound as waxy solid.

^{1}H NMR (600 MHz, $CDCl_3$) δ 7.63 (app. dt, *J* = 6.9, 1.4 Hz, 4H), 7.49 – 7.43 (m, 2H), 7.41 (app. tdd, *J* = 8.1, 3.5, 2.0 Hz, 4H), 6.31 (s, 1H), 5.26 – 5.22 (m, 1H), 3.91 (d, *J* = 10.2 Hz, 1H), 3.82 (d, *J* = 10.3 Hz, 1H), 3.69 (dt, *J* = 12.3, 8.1 Hz, 1H), 3.56 – 3.48 (m, 1H), 2.68 – 2.61 (m, 1H), 2.36 (ddd, *J* = 12.7, 7.5, 3.2 Hz, 1H), 2.25 (d, *J* = 12.2 Hz, 1H), 2.22 – 2.08 (m, 2H), 1.95 (ddt, *J* = 12.1, 7.3, 3.5 Hz, 1H), 1.89 (ddd, *J* = 12.6, 10.0, 8.1 Hz, 1H), 1.76 (dd, *J* = 12.2, 3.4 Hz, 1H), 1.07 (s, 9H).

^{13}C NMR (151 MHz, $CDCl_3$) δ 209.4, 162.0, 142.4, 135.8, 135.7, 132.9, 132.9, 130.2, 128.1, 128.0, 118.6, 70.1, 65.5, 45.6, 44.6, 44.0, 40.8, 29.3, 27.1, 22.4, 19.5.

IR (thin film) ν_{max}/cm^{-1} 2929, 1753, 1662, 1616, 1112

HRMS (ESI) mass calculated for [M+H]+ ($C_{28}H_{34}O_3NSi$) requires *m/z* 460.2302, found *m/z* 460.2303

Compound 26

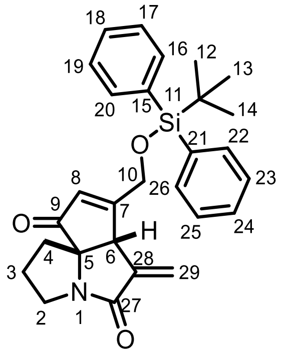

Prepared as above, isolated as a light brown waxy solid.

^{1}**H NMR** (600 MHz, $CDCl_3$) δ 7.62 (m, 4H), 7.47 – 7.42 (m, 2H), 7.39 (td, *J* = 7.1, 5.2 Hz, 4H), 6.33 (t, *J* = 2.0 Hz, 1H), 6.08 (s, 1H), 5.19 (s, 1H), 4.37 (dd, *J* = 15.6, 1.9 Hz, 1H), 4.30 (dd, *J* = 15.7, 2.0 Hz, 1H), 3.68 (s, 1H), 3.65 (td, *J* = 7.1, 3.7 Hz, 1H), 3.57 (dt, *J* = 12.2, 7.5 Hz, 1H), 2.47 (dt, *J* = 13.2, 8.5 Hz, 1H), 2.02 (m, 2H), 1.89 (dt, *J* = 13.0, 5.8 Hz, 1H), 1.07 (s, 9H).

^{13}**C NMR** (151 MHz, $CDCl_3$) (151 MHz, $CDCl_3$) δ 197.0, 161.0, 149.1, 136.6, 135.6, 135.6, 132.9, 132.8, 130.2, 130.2, 128.0, 120.6, 70.9, 60.8, 56.7, 45.5, 26.9, 26.6, 23.4, 19.4.

IR (thin film) ν_{max}/cm^{-1} 2931, 1781, 1663, 1619, 1114

HRMS (ESI) mass calculated for [M+H]+ ($C_{28}H_{32}O_3NSi$) requires *m/z* 458.2146, found *m/z* 458.2153

Compound 27

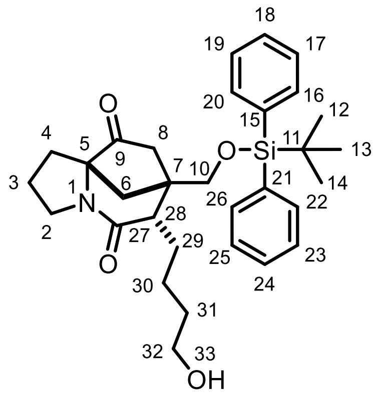

Following a modified literature procedure,[8] a vial was charged with compound **4** (20 mg, 0.044 mmol), [Ir(dF(CF_3)ppy)$_2$(dtbbpy)]PF_6 (0.5 mg, 0.44 μmol), Na_2CO_3 (9.3 mg, 0.088 mmol), MeOH (880 μL), 1-bromo-3-propanol (5.8 μL, 0.066 mmol) then $(TMS)_3SiH$ (10 μL, 0.033 mmol). The mixture was sparged with N_2 for 30 seconds and the vial was sealed. The mixture was irradiated with blue LEDs (450 nm) and stirred for 3.5 hours. H_2O (10 mL) was added and the mixture was extracted with EtOAc (3 x 10 mL). The combined organics were dried with Na_2SO_4, filtered and concentrated under reduced pressure. The crude residue (crude ratio *maj:min* 4.7:1, only major isolated) was purified by PTLC (EtOAc) to yield the title compound as a colourless oil (8.6 mg, 38%).

^{1}**H NMR** (500 MHz, $CDCl_3$) δ 7.62 (m, 4H), 7.47 – 7.37 (m, 6H), 3.72 (d, *J* = 10.4 Hz, 1H), 3.66 – 3.54 (m, 3H), 3.49 (d, *J* = 10.3 Hz, 1H), 3.39 (ddd, *J* = 12.1, 8.5, 3.8 Hz, 1H), 2.65 – 2.57 (m, 1H), 2.38 (dd, *J* = 19.1, 3.4 Hz, 1H), 2.27 (ddd, *J* = 12.5, 7.4, 3.1 Hz, 1H), 2.12 – 2.04 (m, 1H), 2.04 – 1.97 (m, 2H), 1.91 (m, 3H), 1.83 (ddd, *J* = 12.5, 10.0, 7.9 Hz, 1H), 1.71 – 1.44 (m, 5H), 1.34 (dddd, *J* = 11.9, 9.3, 6.0, 3.4 Hz, 1H), 1.07 (s, 9H).

^{13}C NMR (126 MHz, $CDCl_3$) δ 209.6, 171.9, 135.8, 135.7, 132.9, 132.9, 130.2, 130.2, 128.0, 69.1, 67.2, 62.5, 46.5, 45.4, 44.6, 42.2, 39.9, 32.7, 29.0, 27.2, 27.1, 26.5, 22.3, 19.5.

IR (thin film) ν_{max}/cm^{-1} 3408, 2931, 1753, 1630, 1427, 1108

HRMS (ESI) mass calculated for [M+H]+ ($C_{31}H_{42}O_4NSi$) requires *m/z* 520.2878, found *m/z* 520.2896

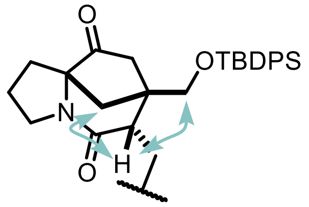

Diagnostic nOe data for compound 27 from 2D NOESY

Compound 29

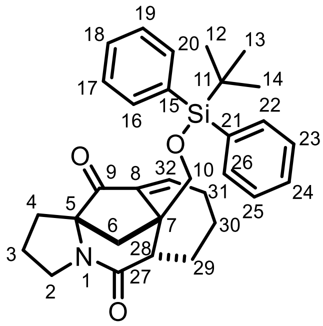

To a solution of compound **27** (5.0 mg, 9.6 μmol) in CH_2Cl_2, cooled to 0 °C, was added $NaHCO_3$ (4.0 mg, 48 μmol) then DMP (6.0 mg, 14 μmol). The mixture was stirred at this temperature for 30 minutes then warmed to room temperature. When the reaction was observed to be complete by TLC, sat. aq. $NaHCO_3$ (1 mL), $Na_2S_2O_3$ (1 mL) and CH_2Cl_2 (2 mL) were added. The layers were separated and the aqueous phase was extracted with CH_2Cl_2 (2 x 2 mL). The combined organics were dried with Na_2SO_4, filtered and concentrated under reduced pressure. The crude residue was purified by FCC (2:1 to 1:1 pentane:EtOAc) to yield the corresponding aldehyde which was used directly in the next step.

MeOH (1 mL) was added, cooled to 0 °C, and to this was added K_2CO_3 (4.0 mg, 0.029 mmol). The reaction mixture was stirred for 3 hours and upon observation of no conversion, KOH (1.6 mg, 0.029 mmol) was added and the reaction mixture was stirred for 13 hours. Upon observation of steady conversion, by TLC and LRMS, the mixture was heated to 70 °C for 6 hours. The MeOH was removed under a stream of N_2 and EtOAc (1 mL) and H_2O (1 mL) was added. The aqueous phase was extracted with EtOAc (3 x 1 mL) and the combined organics were dried with Na_2SO_4, filtered and concentrated

under reduced pressure. The crude residue was dissolved in benzene-d_6 (500 μL) and to this was added $TsOH \cdot H_2O$ (1.0 mg, 5.2 μmol). The mixture was heated in an NMR tube to 60 °C and intermittently monitored by ^1H NMR. After 3 days at 60 °C the mixture was cooled to room temperature and the volatiles were removed under reduced pressure. The crude residue was purified by PTLC (3:2 hexane:EtOAc) to yield the title compound as a colourless oil (1.7 mg, 35%).

^{1}H NMR (600 MHz, $CDCl_3$) δ 7.68 – 7.59 (m, 4H), 7.49 – 7.37 (m, 6H), 6.91 (dd, *J* = 8.9, 6.6 Hz, 1H), 4.24 (d, *J* = 10.3 Hz, 1H), 3.77 (d, *J* = 10.3 Hz, 1H), 3.53 (dt, *J* = 11.9, 8.4 Hz, 1H), 3.41 (ddd, *J* = 12.0, 8.7, 3.3 Hz, 1H), 2.87 (t, *J* = 4.2 Hz, 1H), 2.60 (dd, *J* = 15.0, 3.9 Hz, 1H), 2.36 (ddd, *J* = 12.6, 7.3, 2.6 Hz, 1H), 2.14 – 1.91 (m, 6H), 1.91 – 1.79 (m, 2H), 1.50 (m, 1H), 1.24 – 1.15 (m, 1H), 1.07 (s, 9H).

^{13}C NMR (151 MHz, $CDCl_3$) δ 201.4, 170.0, 145.3, 140.1, 135.8, 135.8, 133.2, 133.1, 130.2, 130.1, 128.0, 128.0, 69.2, 64.6, 47.2, 45.3, 44.3, 41.2, 28.3, 27.1, 26.9, 26.8, 22.3, 21.5, 19.5.

IR (thin film) ν_{max}/cm^{-1} 2931, 1737, 1650, 1112

HRMS (ESI) mass calculated for [M+H]+ ($C_{31}H_{38}O_3NSi$) requires *m/z* 500.2616, found *m/z* 500.2607

Compound 30

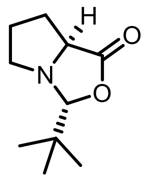

Following a modified literature procedure,[9] to a solution of L-proline (2.0 g, 17 mmol) in hexane (62 mL) was added pivaldehyde (5.6 mL, 52 mmol) and TFA (31 μL). The resulting mixture was refluxed with Dean-Stark apparatus for 48 hours. The volatiles were removed taking care to minimise exposure to air to yield the title compound as a brown solid (3.2 g, 99%). Data were in agreement with the literature.[9]

^{1}H NMR (400 MHz, $CDCl_3$) δ 4.51 (s, 1H), 3.81 (dd, *J* = 8.9, 4.4 Hz, 1H), 3.20 (dt, *J* = 10.4, 6.4 Hz, 1H), 2.80 (dt, *J* = 10.5, 6.4 Hz, 1H), 2.15 (ddt, *J* = 12.9, 8.9, 7.3 Hz, 1H), 2.09 – 1.99 (m, 1H), 1.87 – 1.76 (m, 1H), 1.76 – 1.62 (m, 1H), 0.93 (s, 9H).

^{13}C NMR (101 MHz, $CDCl_3$) δ 178.1, 108.3, 62.9, 58.5, 37.6, 29.8, 25.3, 24.3

Stereochemical configuration was assigned by analogy with the literature.

$[\alpha]_D$ (c = 1.04, $CHCl_3$) = −34.4. (*cf Seebach, c = 2.4, $CHCl_3$) = −24.7)*[9]

Compound S-14

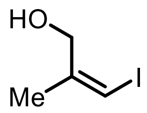

To a suspension of CuI (430 mg, 2.2 mmol) in THF (20 mL), cooled to 0 °C, was added propargyl alcohol (1.3 g, 22 mmol). MeMgBr (3 M solution in Et_2O, 13 mL, 44 mmol) was added dropwise and the resulting mixture was stirred at 0 °C for 1.5 hours. A solution of I_2 (8.4 g, 33 mmol) in Et_2O (28 mL, an additional 2 x 20 mL used to rinse the flask) was added and the reaction mixture was stirred for a further 1.5 hours. Sat. aq. NH_4Cl (15 mL), sat. aq. $Na_2S_2O_3$ (15 mL) and H_2O (15 mL) were added and the mixture was warmed to room temperature. The aqueous layer was extracted with Et_2O (3 x 100 mL) and the combined organics were washed with brine (50 mL). The combined organics were dried with Na_2SO_4, filtered and concentrated under reduced pressure. The crude residue was purified by FCC (19:1 to 4:1 pentane:EtOAc) to yield the title compound as a colourless oil (2.6 g, 59%). Data were in agreement with the literature.[10]

^{1}H NMR (400 MHz, $CDCl_3$) δ 5.98 (dh, *J* = 1.4, 0.7 Hz, 1H), 4.25 (d, *J* = 6.2 Hz, 2H), 1.98 (d, *J* = 1.5 Hz, 3H).

^{13}C NMR (101 MHz, $CDCl_3$) δ 146.1, 74.9, 68.2, 21.7.

Compound 32

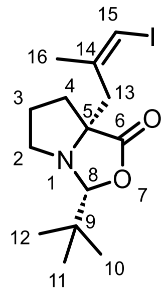

*Preparation of allyl bromide **31***

Due to its instability under ambient conditions, allyl bromide **31** was freshly prepared, following a modified literature procedure,[11] for use in the alkylation reaction. A solution of **S-14** (2.6 g, 13 mmol) and PPh_3 (4.6 g, 17 mmol) in CH_2Cl_2 (67 mL) was cooled to −20 °C and to this was added NBS (3.6 g, 20 mmol). The resulting orange solution was stirred for 1 hour and pentane (100 mL) was added. The suspension was filtered through a silica plug, eluting with cold pentane. The filtrate was evaporated and the crude residue

was suspended in pentane and filtered and this was repeated once more. Evaporation yielded allyl bromide **31** as a colourless oil (2.4 g, 69%).

Alkylation procedure

A solution of diisopropylamine (1.15 mL, 8.42 mmol) in THF (17 mL) was cooled to −78 °C and *n*-BuLi (5.34 mL, 1.6 M solution in hexanes, 8.54 mmol) was added. The solution was stirred for 30 minutes at this temperature before dropwise addition of a solution of compound **30** (1.1 g, 6.1 mmol) in THF (12 mL). The reaction mixture was stirred for 30 minutes at −78 °C. The electrophile was added (17 mL, 0.54 M in THF, 9.2 mmol) and the mixture was warmed to –40 °C and stirred for 4 hours. H_2O and brine were added and the resulting biphasic mixture was extracted with $CHCl_3$ (3 x 50 mL). The combined organics were dried with Na_2SO_4, filtered and concentrated under reduced pressure. The crude residue was purified by FCC (1:0 to 9:1 to 8:2 pentane:EtOAc), the title compound was obtained as a low-melting waxy solid (890 mg, 40% w.r.t. compound **30**).

^{1}H NMR (400 MHz, $CDCl_3$) δ 6.15 (d, *J* = 1.5 Hz, 1H), 4.26 (s, 1H), 3.04 (ddd, *J* = 12.1, 9.7, 5.9 Hz, 1H), 2.95 (d, *J* = 13.5 Hz, 1H), 2.82 (ddd, *J* = 12.0, 6.7, 3.3 Hz, 1H), 2.41 (d, *J* = 13.6 Hz, 1H), 2.07 – 1.99 (m, 5H), 1.82 (ddddd, *J* = 10.1, 6.5, 5.9, 4.4, 3.3 Hz, 1H), 1.70 – 1.61 (m, 1H), 0.94 (s, 9H).

^{13}C NMR (101 MHz, $CDCl_3$) δ 178.1, 144.1, 105.9, 80.4, 72.4, 58.0, 44.7, 36.6, 35.5, 24.9 (app. d, *J* = 1.6 Hz), 24.4.

IR (thin film) ν_{max}/cm^{-1} 2973, 1778, 1192

HRMS (ESI) mass calculated for [M+H]+ ($C_{14}H_{23}O_2NI$) requires *m/z* 364.0768, found *m/z* 364.0769

$[\alpha]_D$ (c = 0.98, $CHCl_3$) = +73.3.

<u>Compound 33</u>

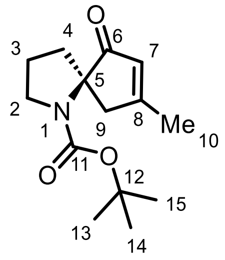

To a solution of compound **32** (880 mg, 2.4 mmol) in Et_2O (24 mL), cooled to −78 °C, was added *t*-BuLi (1.6 M in pentane, 2.4 mL, 3.9 mmol). After 20 minutes, sat. aq. K_2CO_3 (10 mL) and H_2O (10 mL) were

added and the mixture was stirred for 5 minutes. A solution of Boc_2O (1.6 mg, 7.3 mmol) in THF (10 mL) was added and the mixture was stirred overnight at room temperature. The aqueous phase was extracted with EtOAc (3 x 50 mL) and the combined organics were dried with Na_2SO_4, filtered and concentrated under reduced pressure. The crude residue was purified by FCC (2:1 to 1:1 pentane:EtOAc) to yield the title compound as a white solid (490 mg, 81%).

^{1}H NMR (400 MHz, $CDCl_3$, mixture of rotamers) δ 6.00 (h, J = 1.4 Hz, $1H_{min}$), 5.97 – 5.93 (m, $1H_{maj}$), 3.62 – 3.46 (m, 2H), 3.10 (d, J = 17.6 Hz, $1H_{min}$), 2.90 (d, J = 17.9 Hz, $1H_{maj}$), 2.52 – 2.36 (m, 1H), 2.10 (app. dp, J = 3.4, 1.1 Hz, 3H), 2.08 – 1.93 (m, 2H), 1.90 – 1.70 (m, 2H), 1.42 (d, J = 1.3 Hz, $9H_{min}$), 1.31 (d, J = 1.2 Hz, $9H_{maj}$).

^{13}C NMR (101 MHz, $CDCl_3$, mixture of rotamers) δ 208.2, 208.2, 174.1, 173.7, 153.5, 153.2, 128.7, 128.5, 80.3, 79.9, 69.4, 69.0, 48.3, 48.1, 47.9, 46.7, 40.1, 39.1, 28.6, 28.4, 24.0, 23.4, 20.0, 19.6.

MP 40-42 °C

IR (thin film) ν_{max}/cm^{-1} 2974, 1715, 1693, 1391

HRMS (ESI) mass calculated for [M+Na]+ ($C_{14}H_{21}O_3NNa$) requires *m/z* 274.1414, found *m/z* 274.1411

[α]D (c = 1.00, $CHCl_3$) = +29.4.

Compound S-15

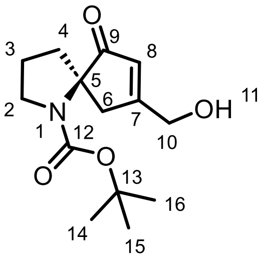

To a solution of compound **33** (58 mg, 0.23 mmol) and TMSCl (110 μL, 0.87 mmol) in THF (2.3 mL) was cooled to −78 °C and to this was added LiHMDS (1 M solution in THF, 0.76 mL, 0.76 mmol). The mixture was warmed to −20 °C and stirred for 2 hours. EtOAc (2 mL) and sat. aq. $NaHCO_3$ (2 mL) were added. The organic phase was washed with sat. aq. $NaHCO_3$ (2 mL), dried with Na_2SO_4, filtered and concentrated under reduced pressure. EtOH (2.3 mL) was added followed by $NaHCO_3$ (60 mg, 0.71 mmol) and *m*CPBA (60 mg, 0.35 mmol, purified according to a literature procedure).[12] The resulting mixture was stirred at room temperature overnight. Sat. aq. $NaHCO_3$ (2 mL) and sat. aq. $Na_2S_2O_3$ (2 mL) were added and the aqueous phase was extracted with CH_2Cl_2 (3 x 2 mL). The combined organics were

dried with Na_2SO_4, filtered and concentrated under reduced pressure. The crude residue was purified by FCC (1:1 pentane:EtOAc) to yield the title compound as a colourless oil (27 mg, 44%) as well as residual starting material (13 mg, 22%).

^{1}H NMR (400 MHz, $CDCl_3$, mixture of rotamers) δ 6.23 (p, *J* = 1.7 Hz, $1H_{min}$), 6.19 (p, *J* = 1.7 Hz, $1H_{maj}$), 4.54 – 4.35 (m, 2H), 3.62 – 3.43 (m, 2H), 3.07 (dt, *J* = 17.3, 1.4 Hz, $1H_{min}$), 2.94 – 2.85 (m, $1H_{maj}$), 2.55 – 2.36 (m, $2H_{min}$+$2H_{maj}$), 2.14 – 1.94 (m, $2H_{min}$+$2H_{maj}$), 1.94 – 1.70 (m, $2H_{min}$+$2H_{maj}$), 1.41 (s, $9H_{min}$), 1.32 (s, $9H_{maj}$).

^{13}C NMR (101 MHz, $CDCl_3$, mixture of rotamers) δ 207.5, 207.5, 176.3, 175.8, 153.6, 153.2, 126.1, 125.8, 80.6, 80.1, 68.8, 68.5, 63.0, 62.9, 48.3, 48.1, 43.0, 41.9, 40.2, 39.1, 28.6, 28.4, 24.0, 23.4.

IR (thin film) ν_{max}/cm^{-1} 3435, 2976, 1696, 1622, 1395

HRMS (ESI) mass calculated for [M+Na]+ ($C_{14}H_{21}O_4NNa$) requires *m/z* 290.1363, found *m/z* 290.1361

[α]$_D$ (c = 0.16, $CHCl_3$) = −10.4.

<u>Compound (R)-11</u>

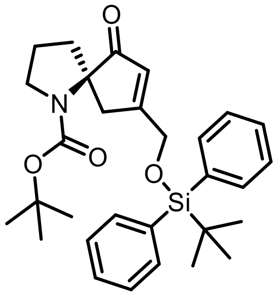

To a solution of **S-15** (10.0 mg, 0.037 mmol) in CH_2Cl_2 (300 μL) was added TBDPSCl (17 μL, 0.067 mmol) and imidazole (7.5 mg, 0.111 mmol). The reaction mixture was stirred overnight at room temperature. H_2O (1 mL) was added and the resulting biphasic mixture was extracted with CH_2Cl_2 (3 x 2 mL). The combined organics were dried with Na_2SO_4, filtered and concentrated under reduced pressure. The crude residue was purified by PTLC (hexane:EtOAc 2:1) to afford a colourless oil (14.7 mg, 79%).

Data were consistent with those obtained previously – see compound **11**.

[α]$_D$ (c = 1.36, $CHCl_3$) = +25.2

NMR Spectra

Compound 9 (^{1}H NMR, 400 MHz, $CDCl_3$)

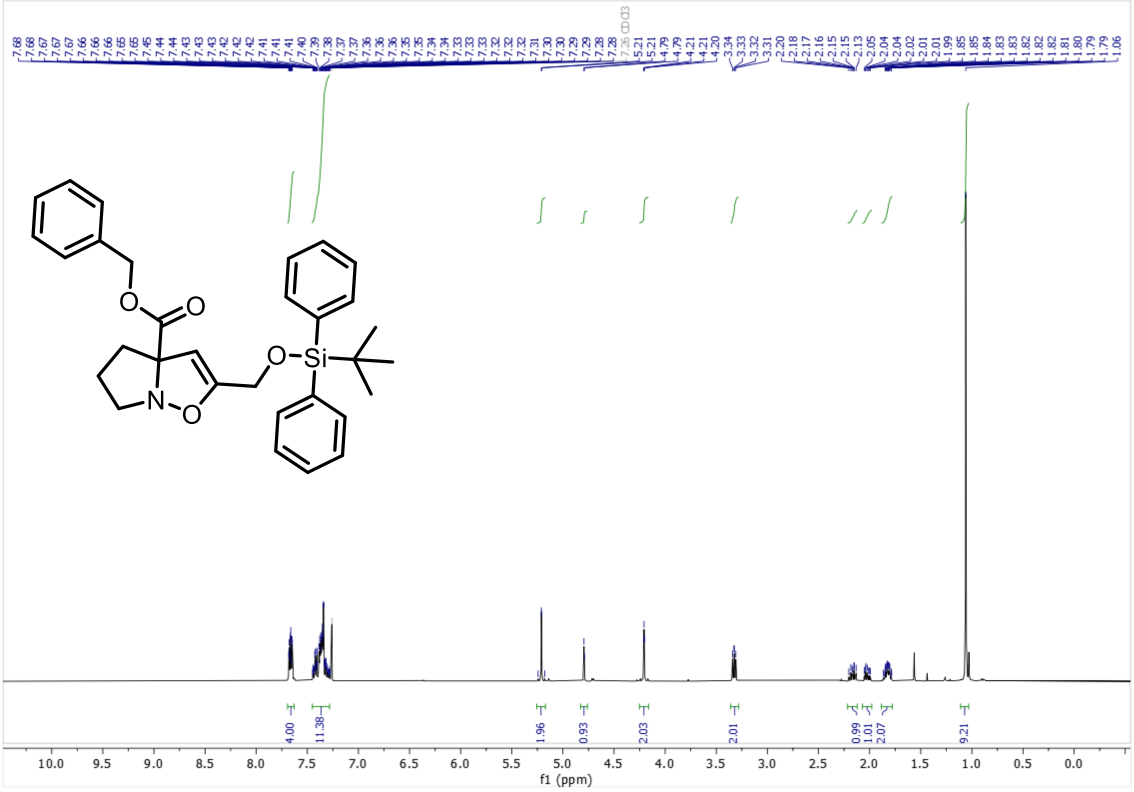

Compound 9 (^{13}C NMR, 101 MHz, $CDCl_3$)

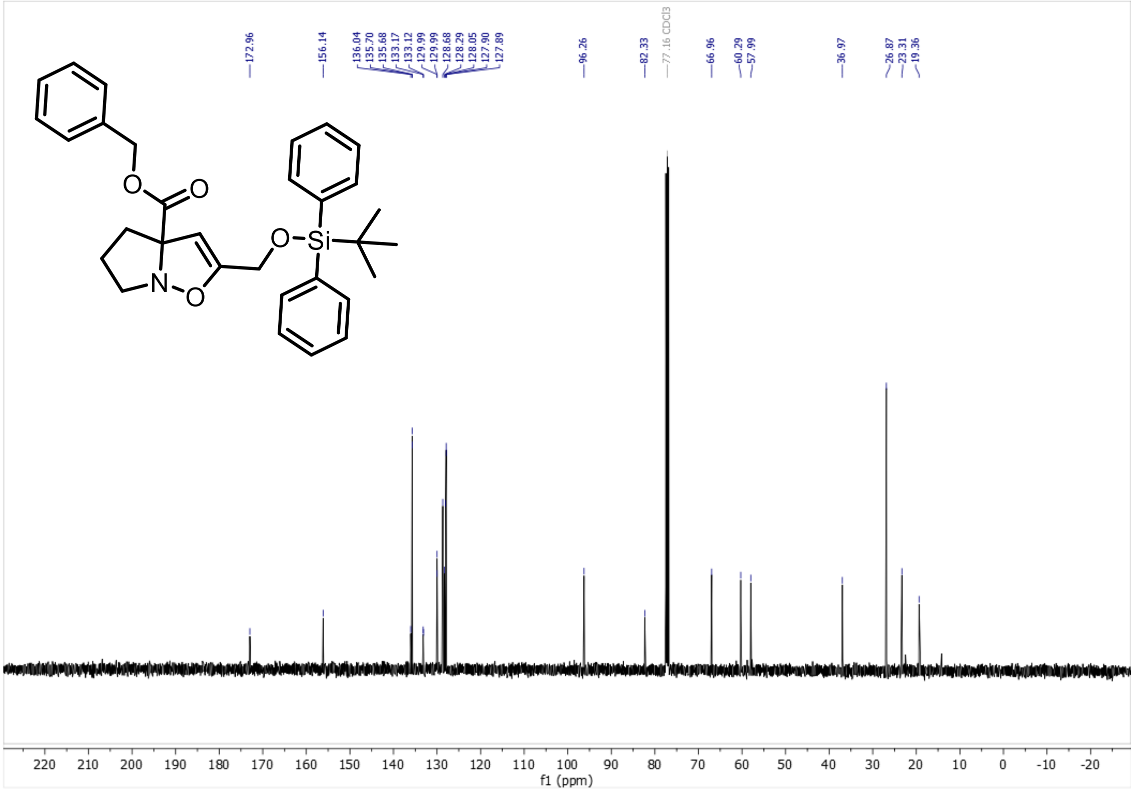

Compound 10 (^{1}H NMR, 400 MHz, $CDCl_3$)

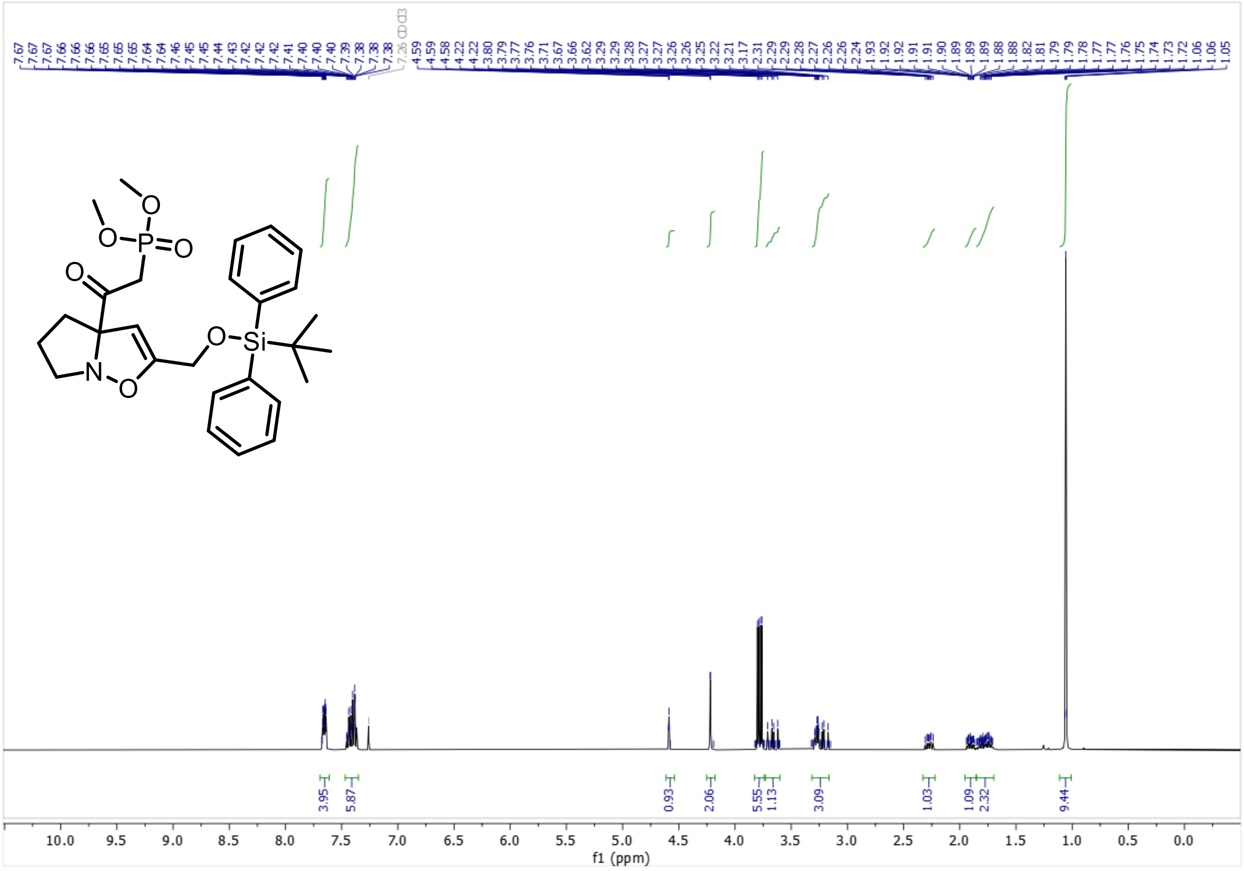

Compound 10 (^{13}C NMR, 101 MHz, $CDCl_3$)

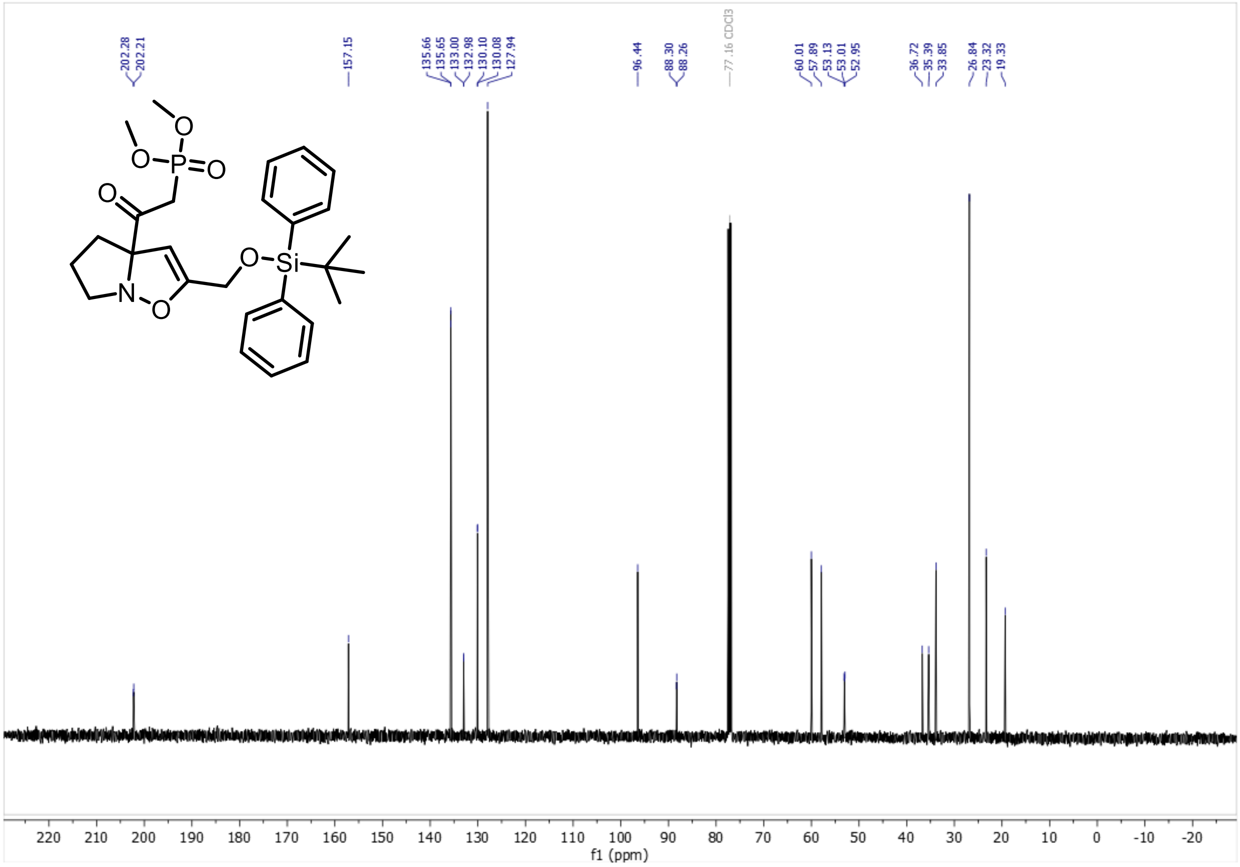

Compound 11 (^1H NMR, 400 MHz, $CDCl_3$)

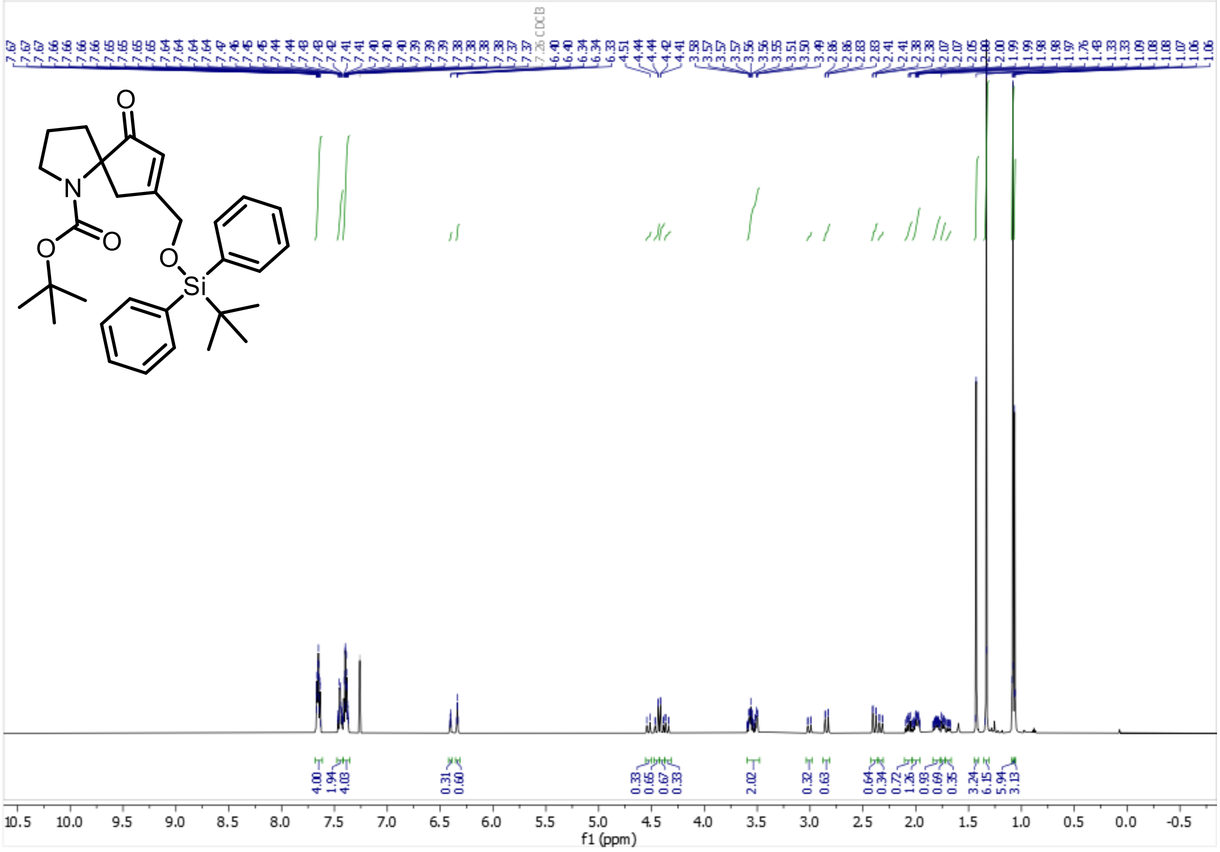

Compound 11 (^{13}C NMR, 151 MHz, $CDCl_3$)

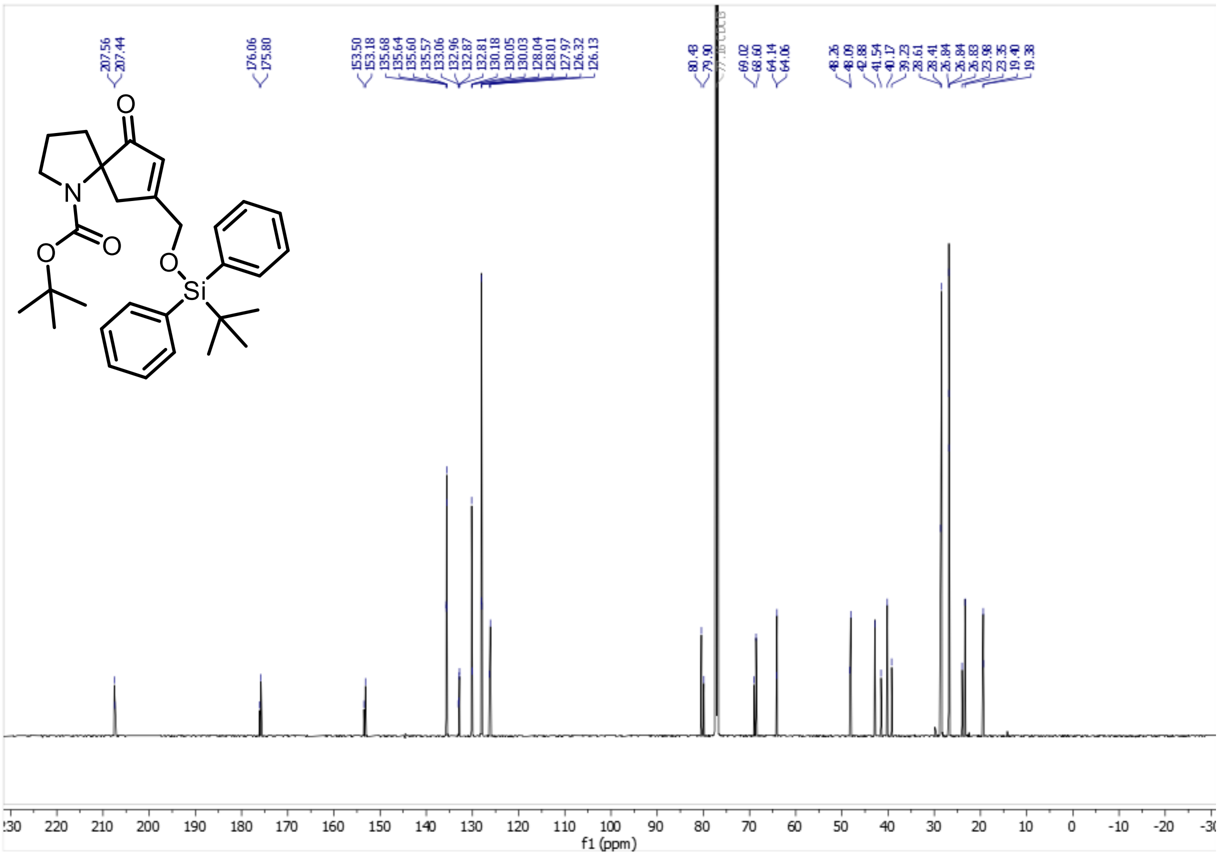

Compound S-2A (^1H NMR, 400 MHz, $CDCl_3$)

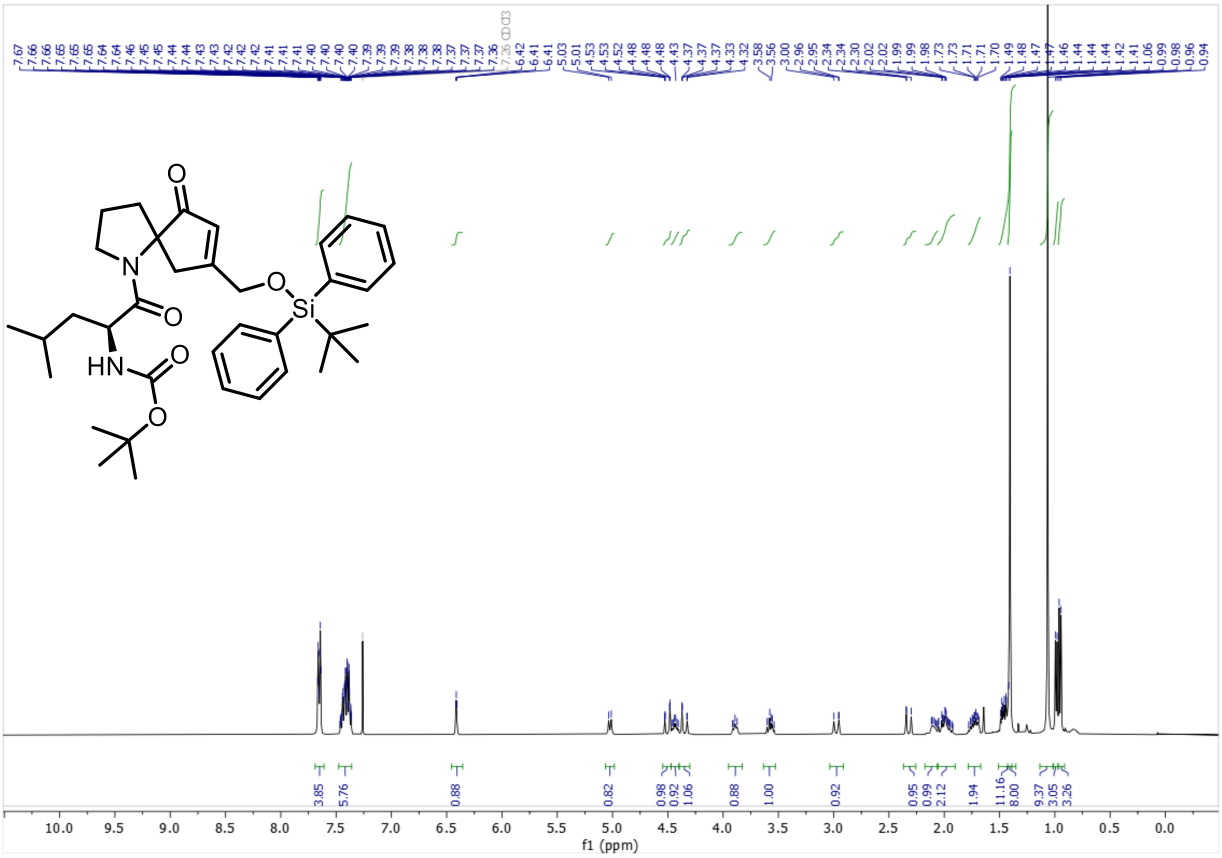

Compound S-2A (^{13}C NMR, 101 MHz, $CDCl_3$)

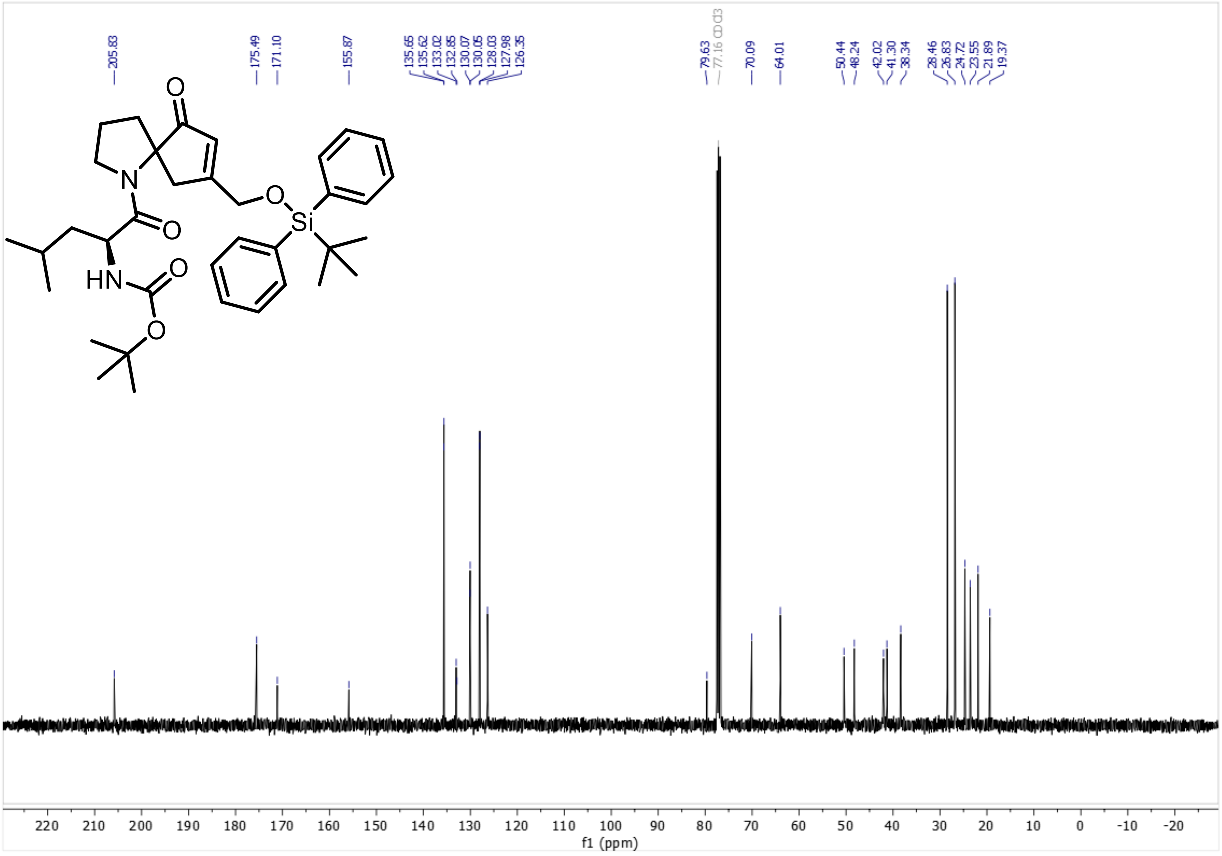

Compound S-2B (^{1}H NMR, 400 MHz, $CDCl_3$)

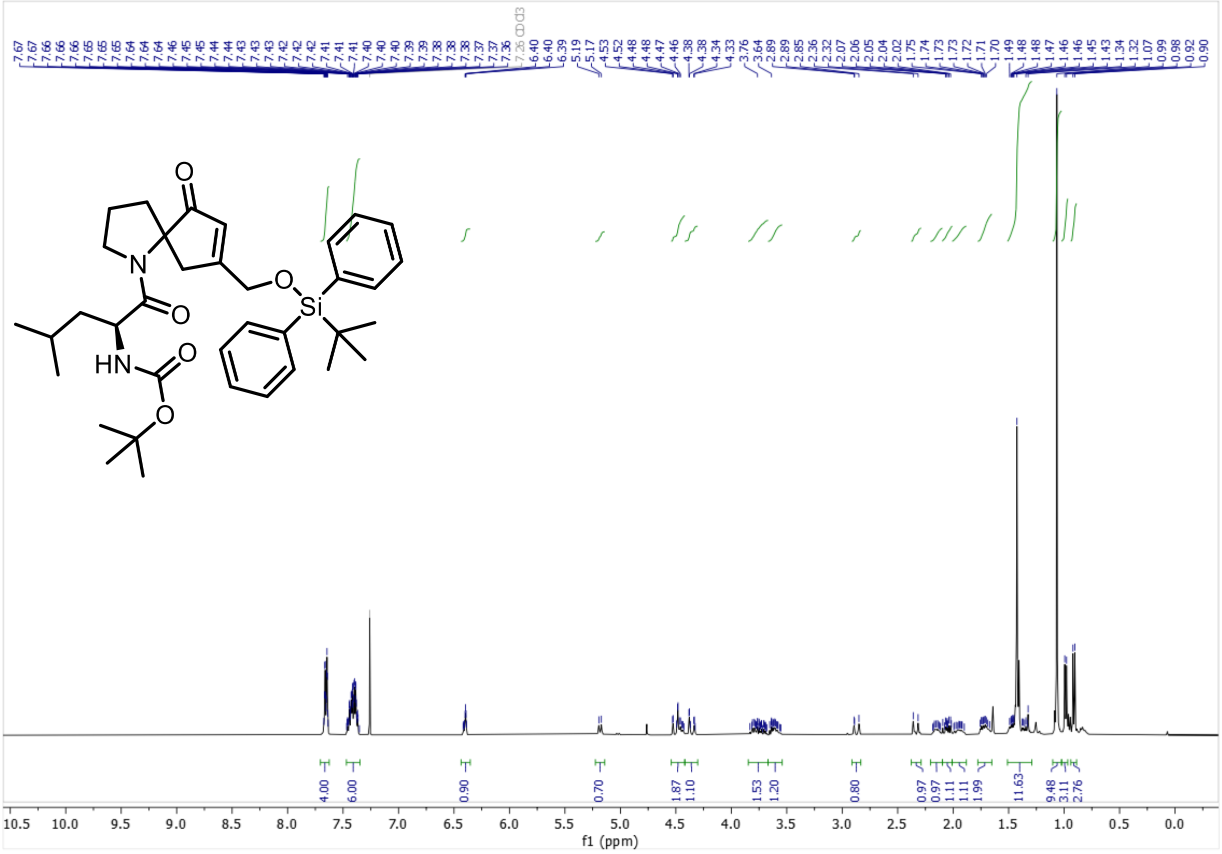

Compound S-2B (^{13}C NMR, 101 MHz, $CDCl_3$)

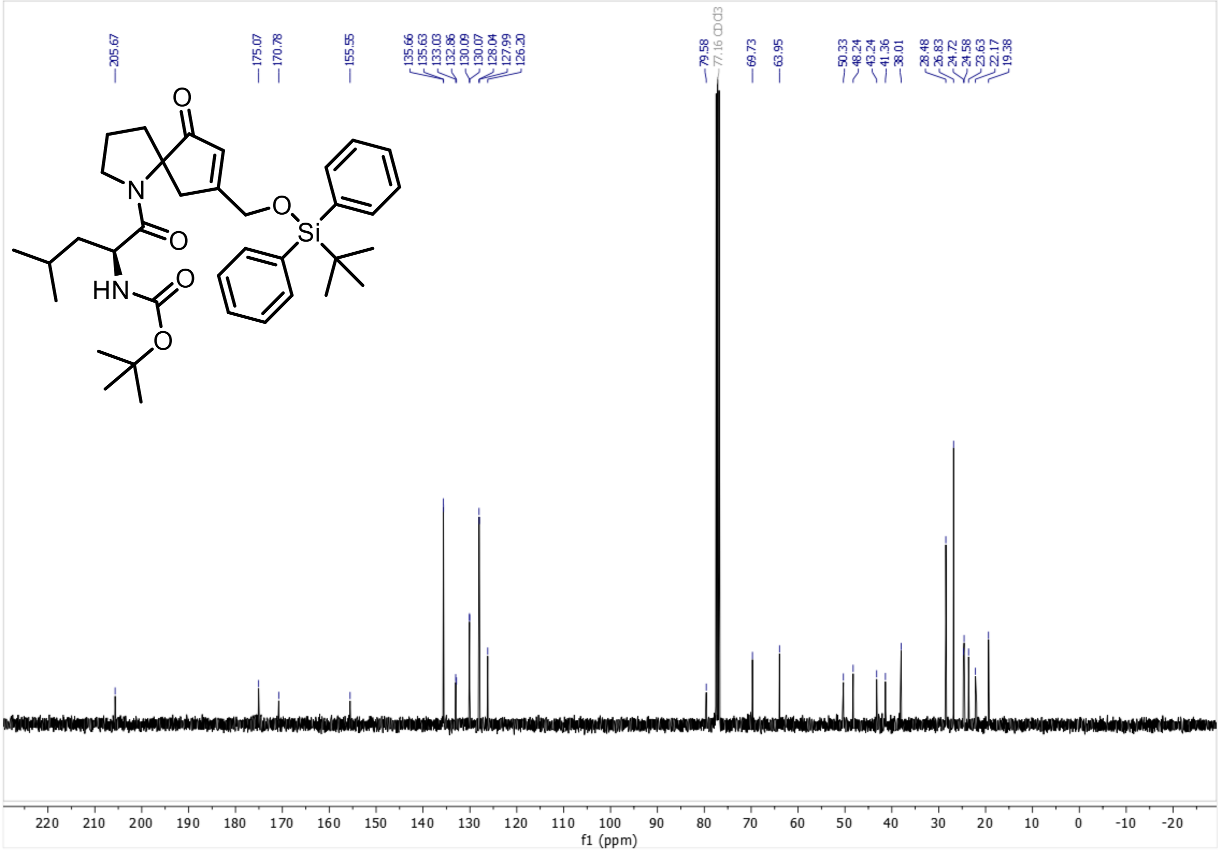

Compound S-3 (^{1}H NMR, 400 MHz, $CDCl_3$)

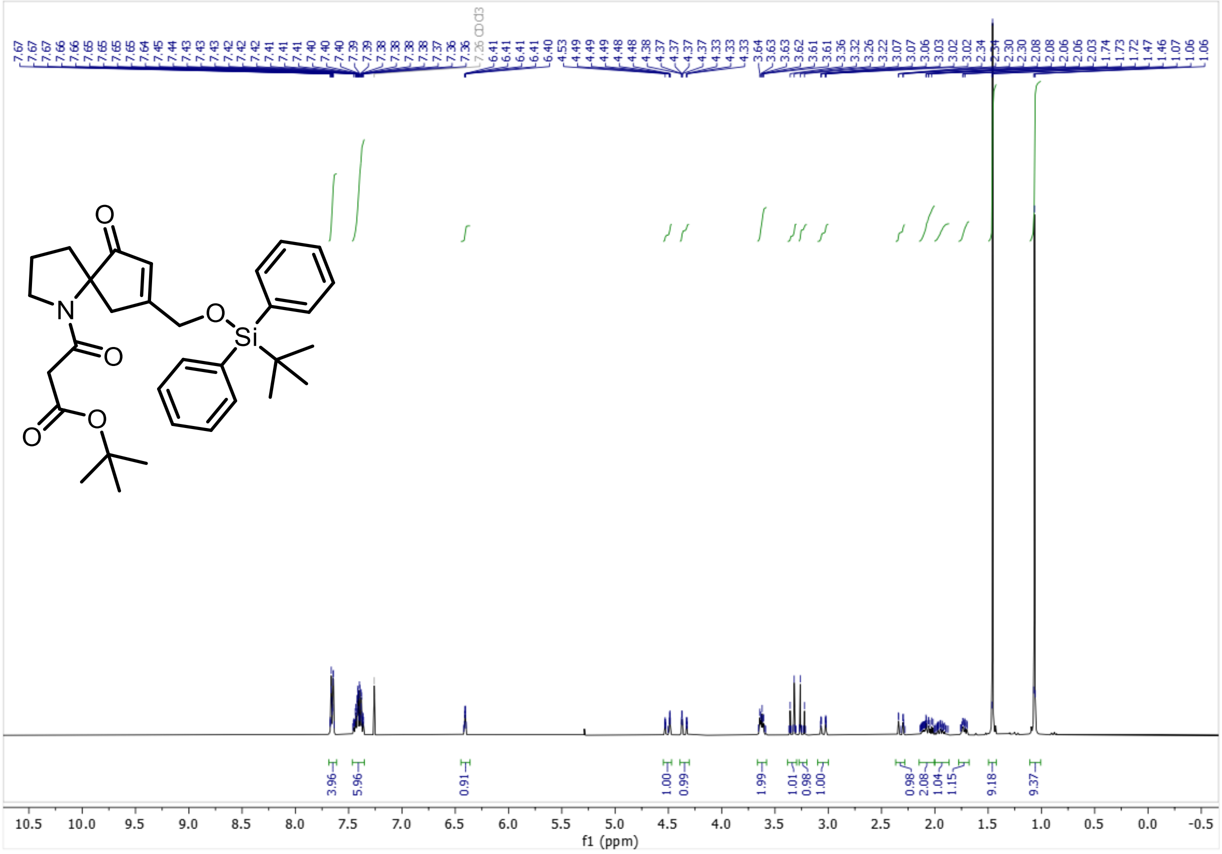

Compound S-3 (^{13}C NMR, 101 MHz, $CDCl_3$)

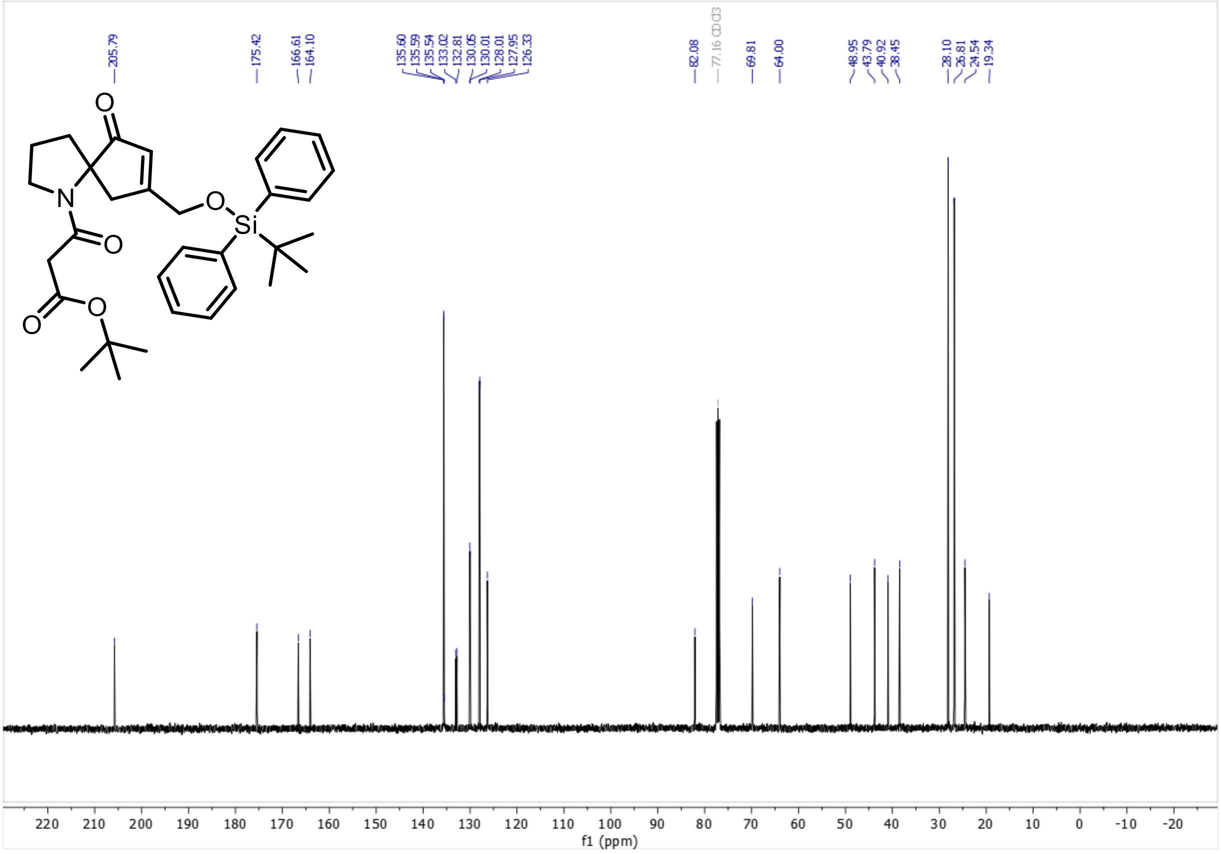

Compound S-4 (^{1}H NMR, 600 MHz, CDCl$_3$)

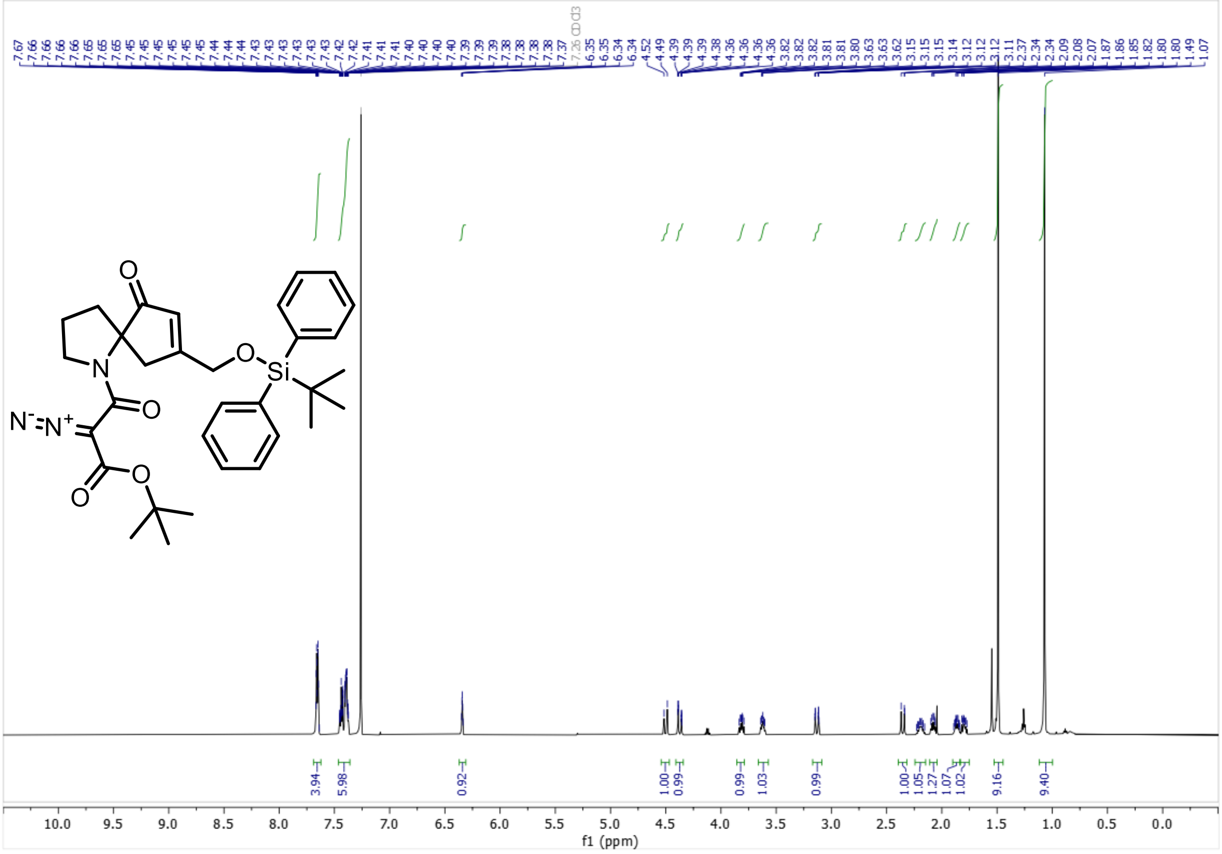

Compound S-4 (^{13}C NMR, 151 MHz, CDCl$_3$)

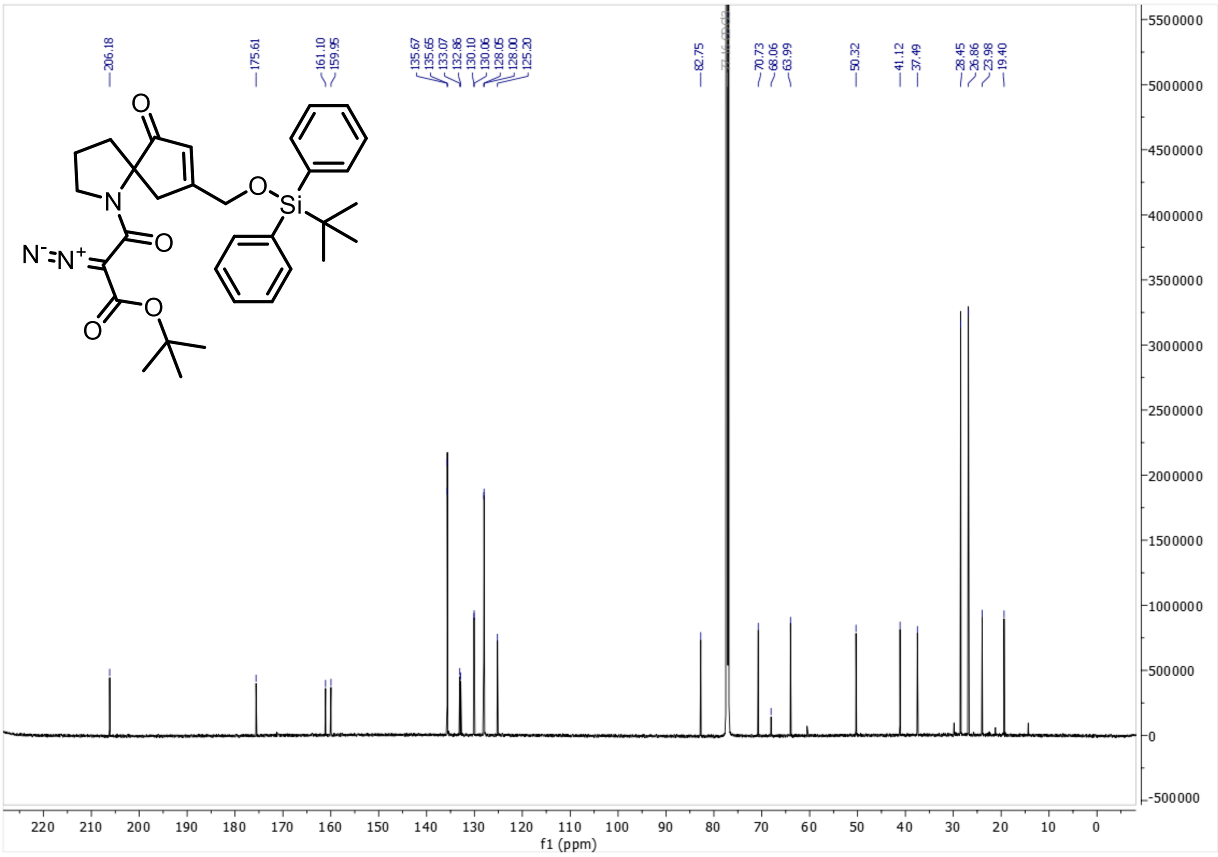

Compound S-5 (^{1}H NMR, 400 MHz, $CDCl_3$)

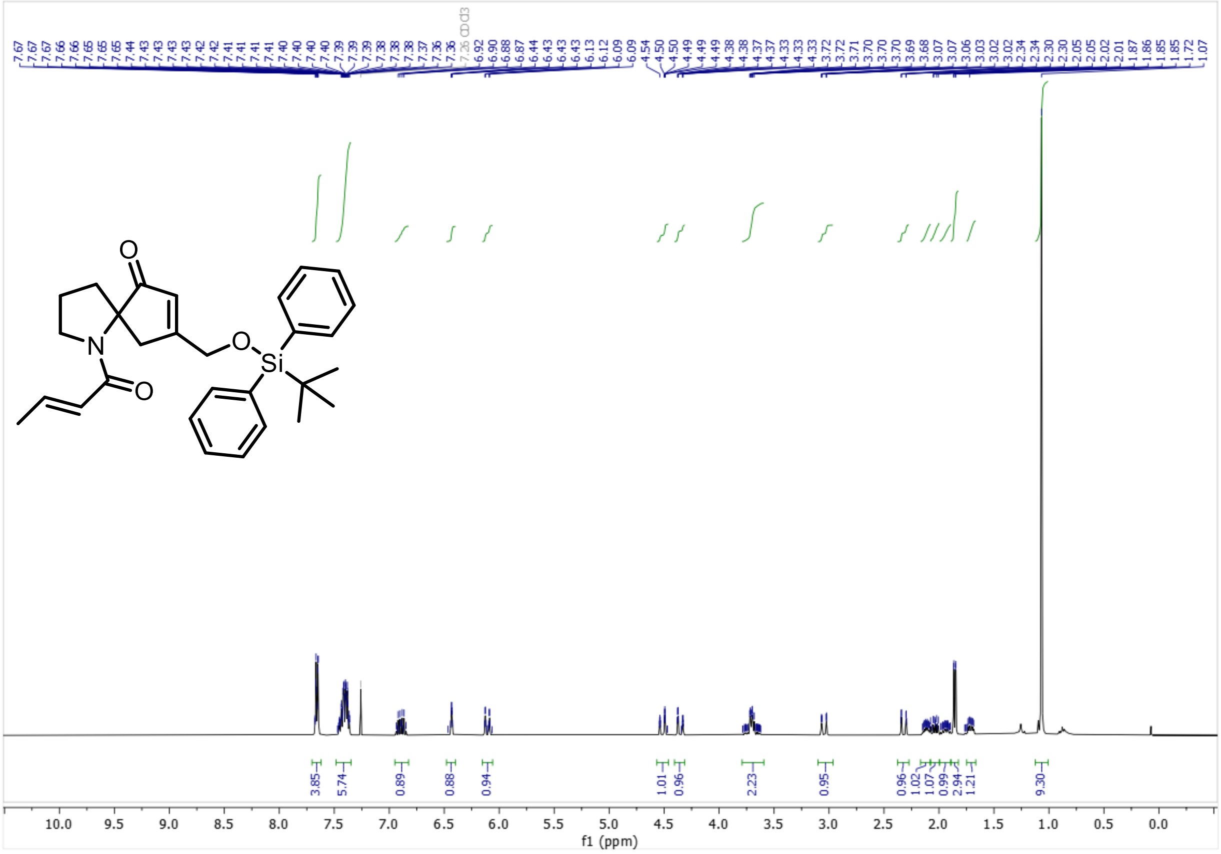

Compound S-5 (^{13}C NMR, 101 MHz, $CDCl_3$)

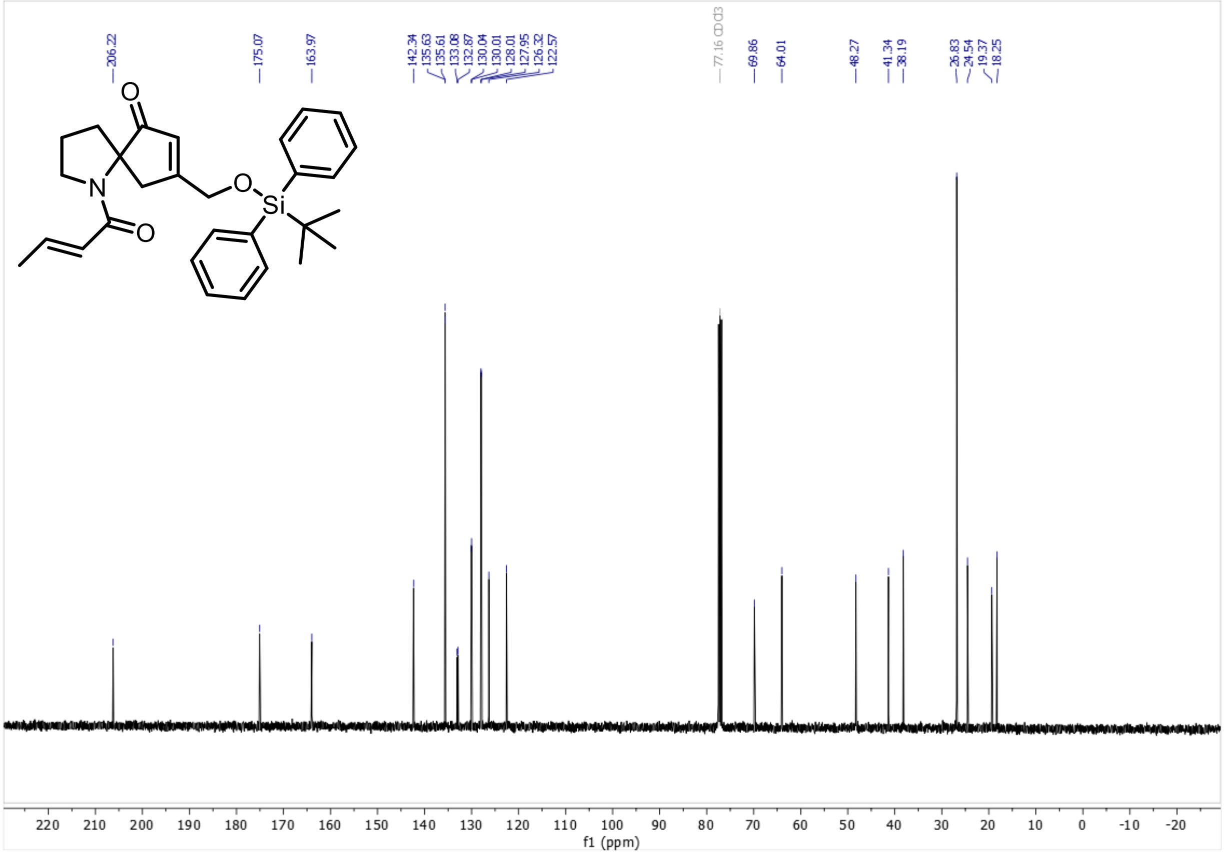

Compound S-6 (^{1}H NMR, 400 MHz, $CDCl_3$)

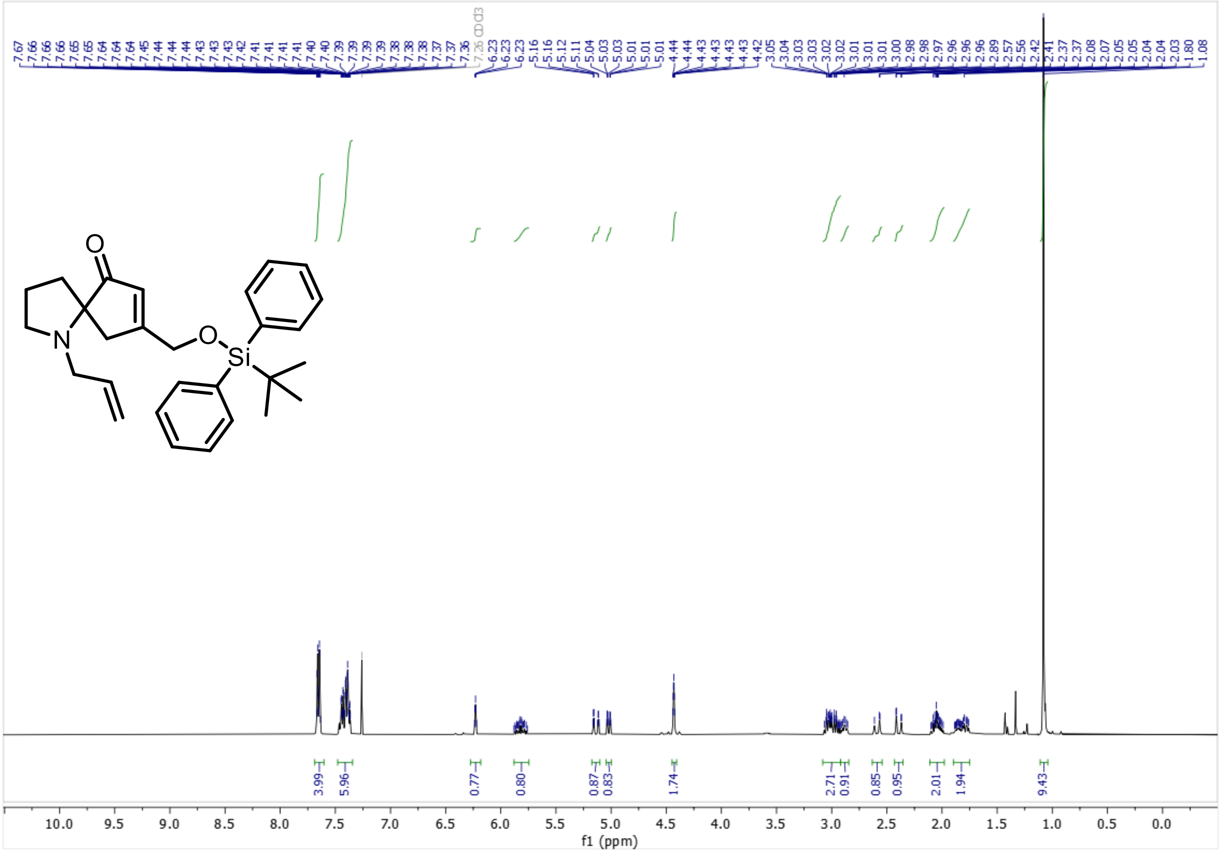

Compound S-6 (^{13}C NMR, 101 MHz, $CDCl_3$)

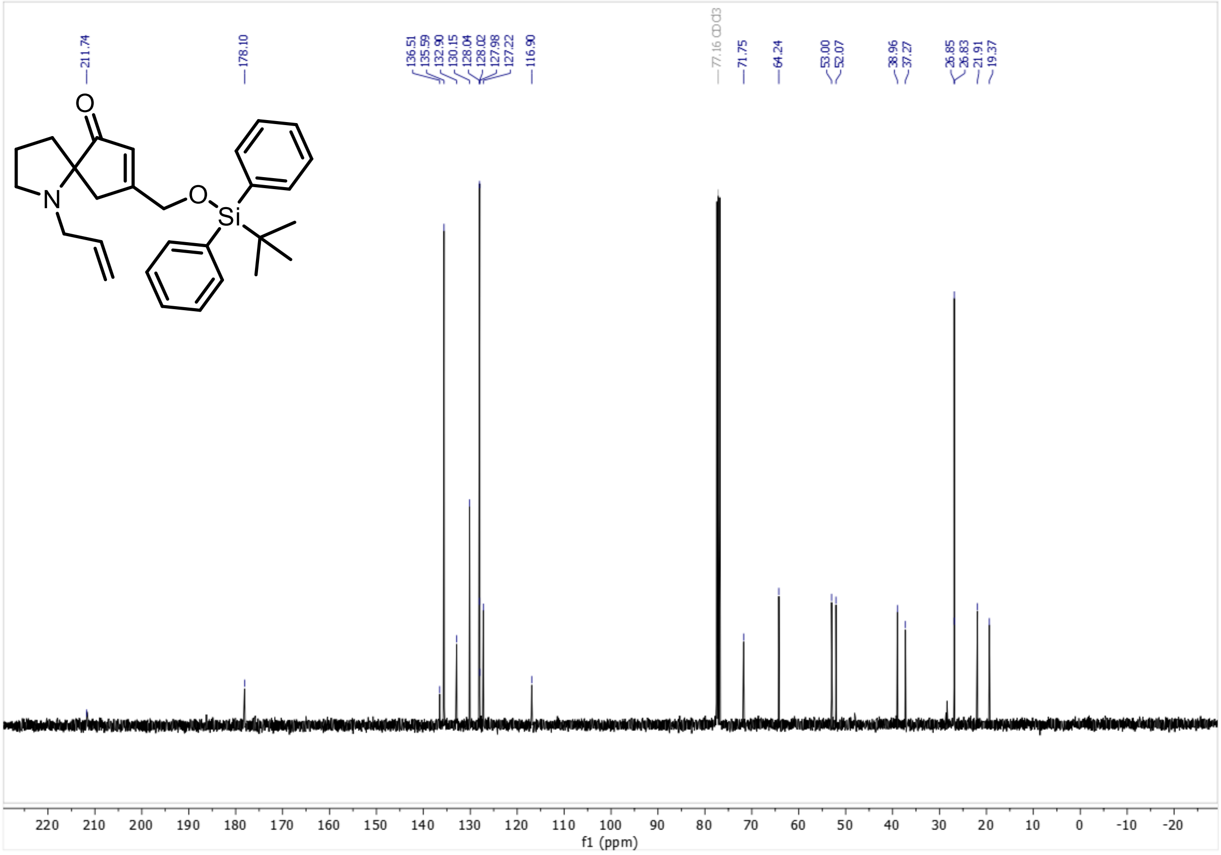

Compound 25 (^{1}H NMR, 400 MHz, $CDCl_3$)

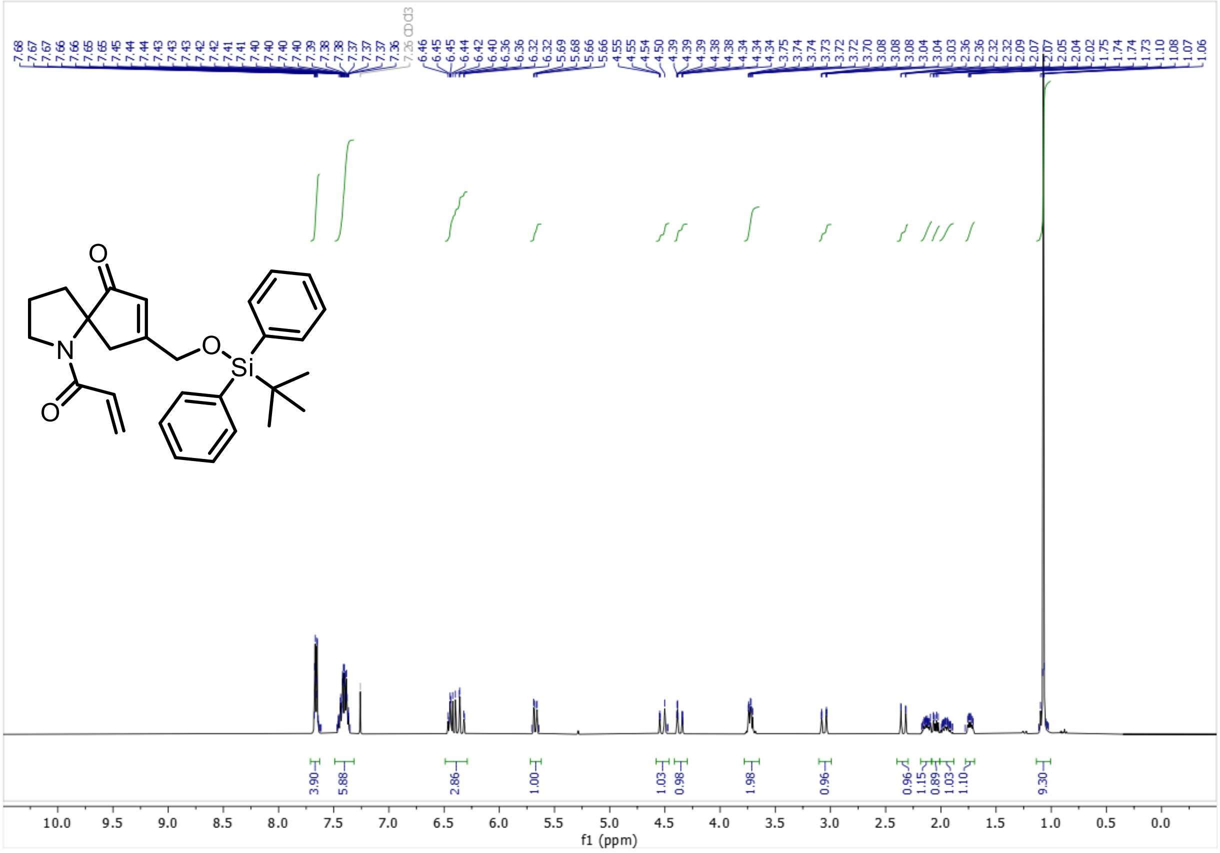

Compound 25 (^{13}C NMR, 101 MHz, $CDCl_3$)

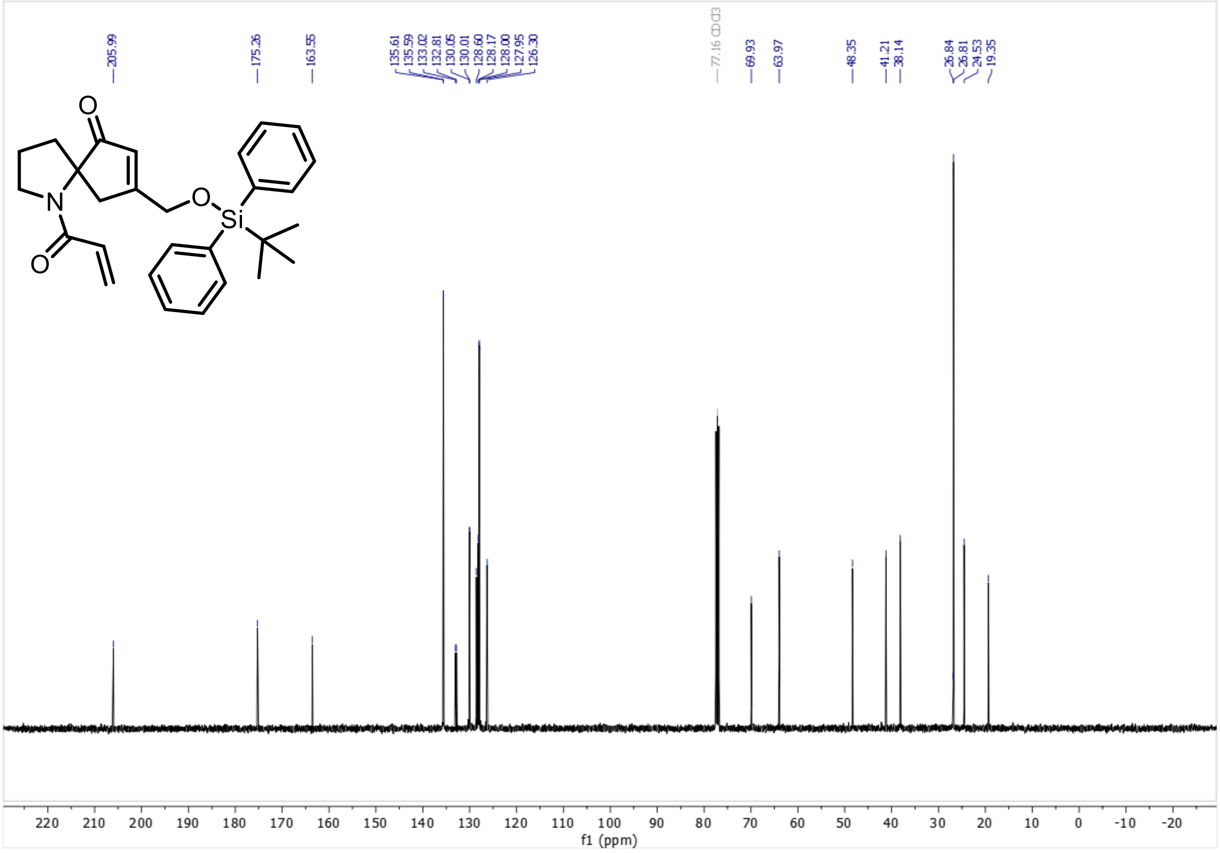

Compound S-7 (^{1}H NMR, 400 MHz, $CDCl_3$)

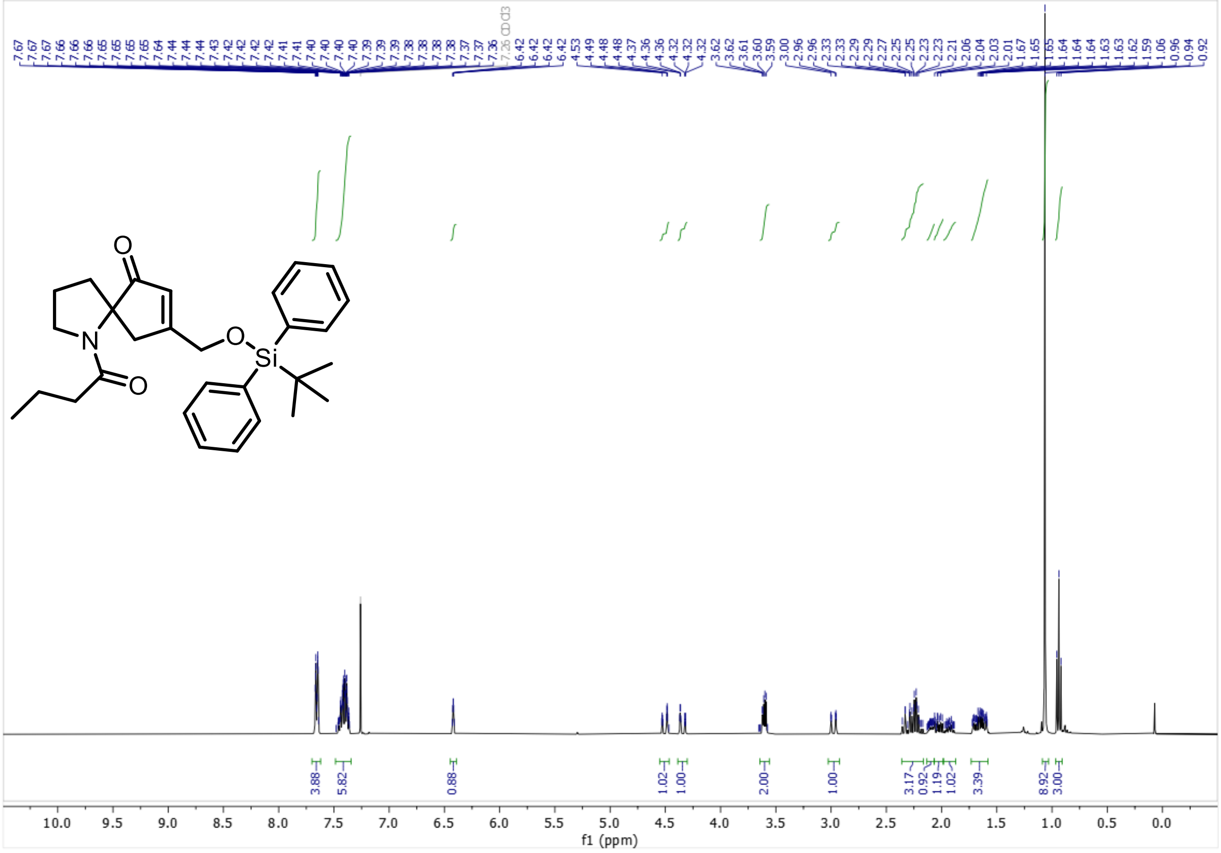

Compound S-7 (^{13}C NMR, 101 MHz, $CDCl_3$)

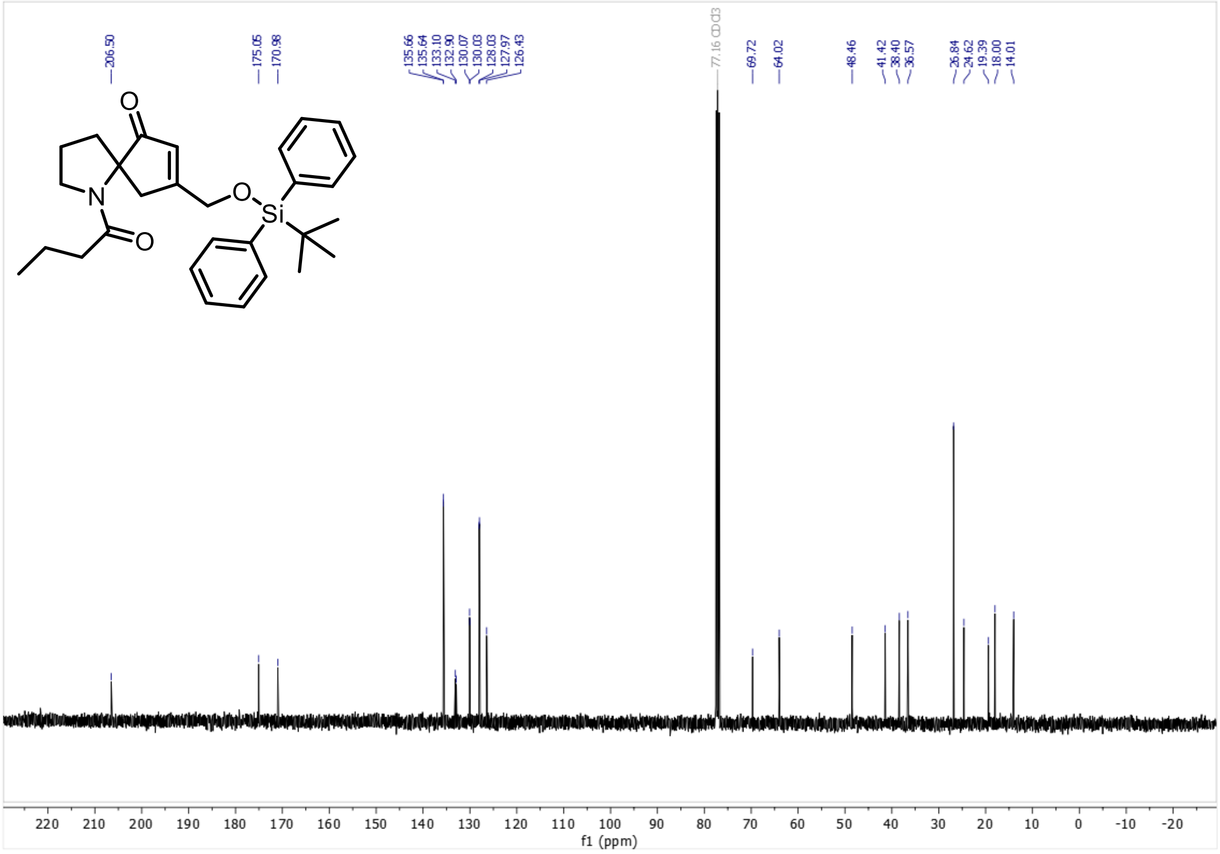

Compound S-8 (^{1}H NMR, 600 MHz, $CDCl_3$)

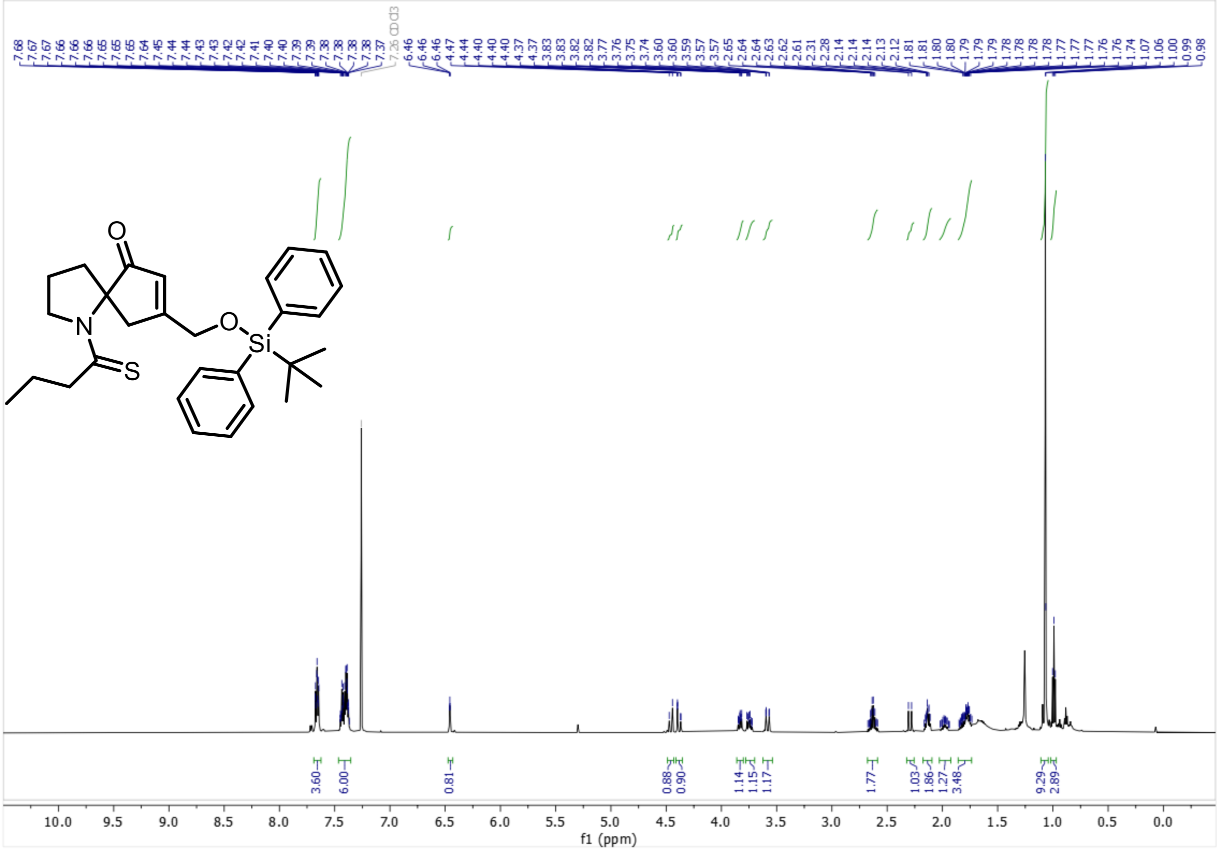

Compound S-8 (^{13}C NMR, 151 MHz, $CDCl_3$)

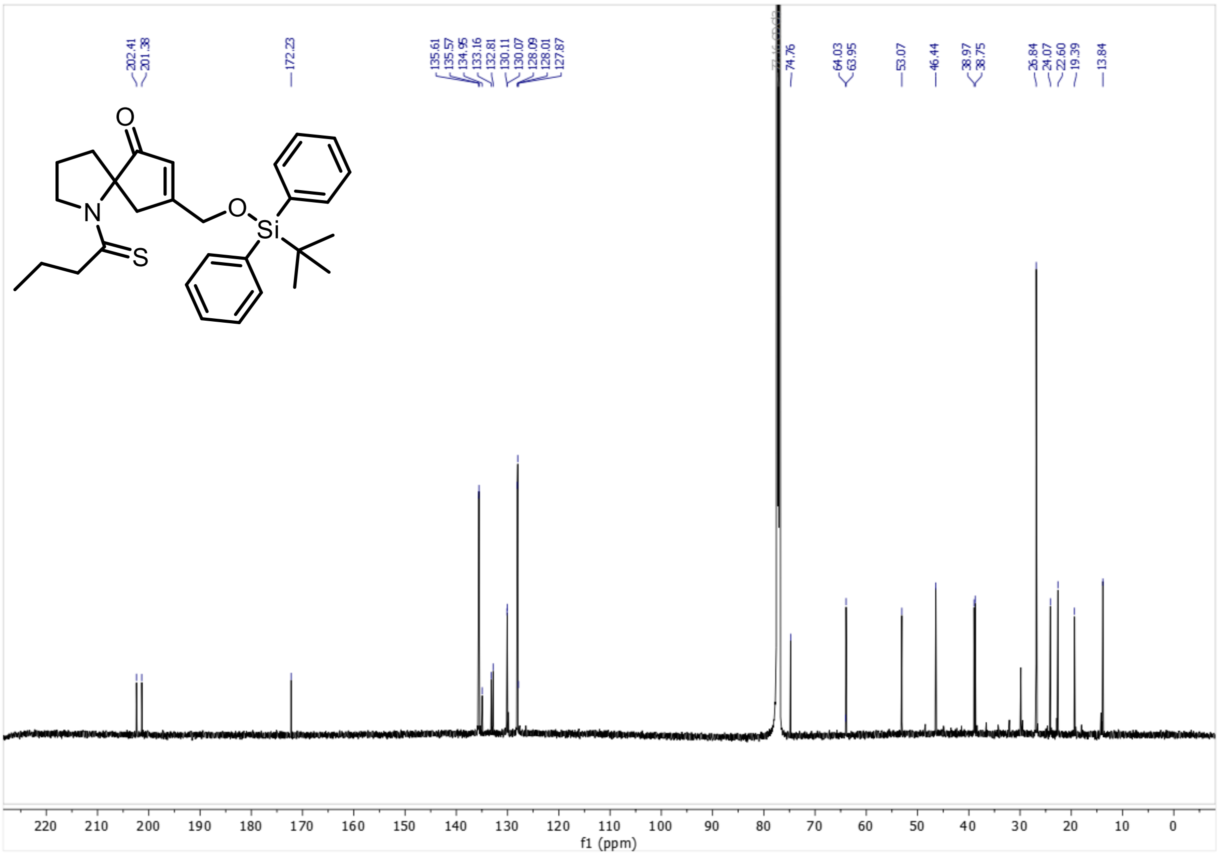

Compound 12 (^{1}H NMR, 400 MHz, $CDCl_3$)

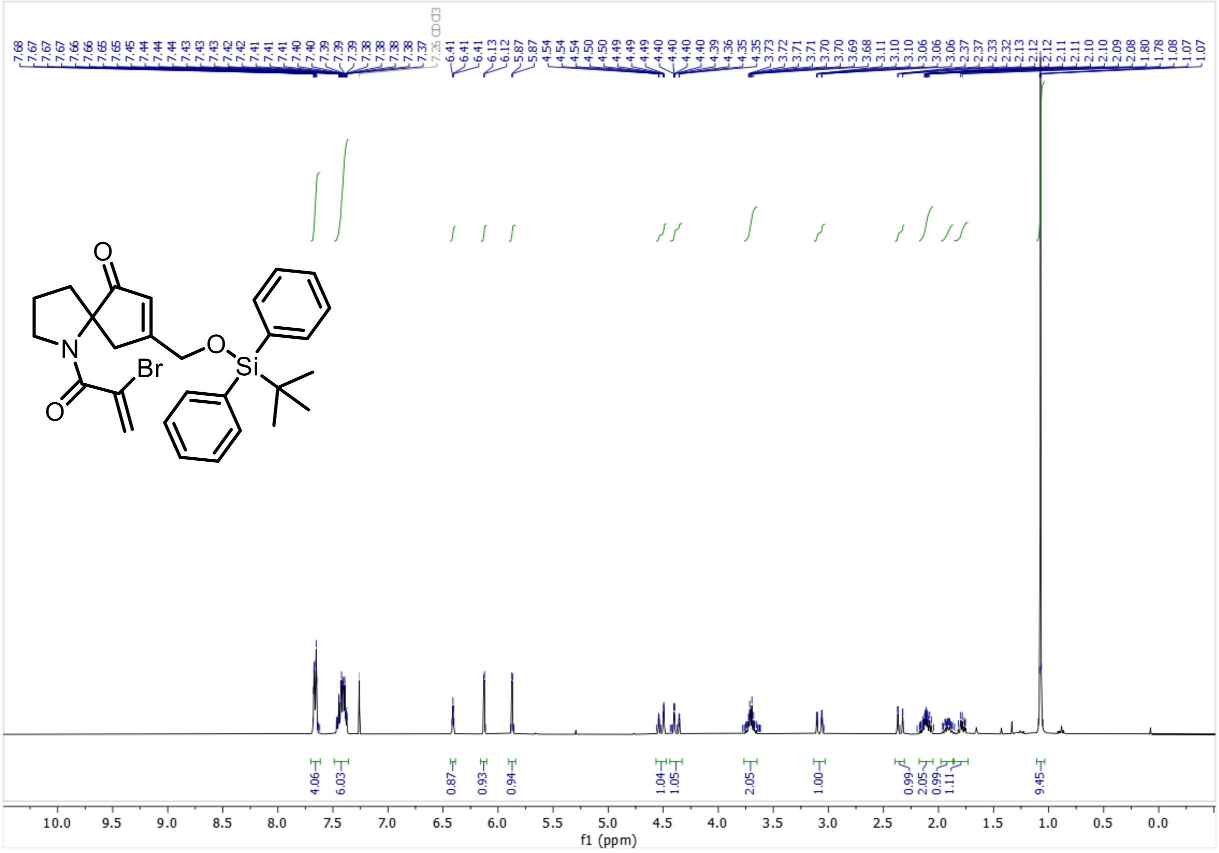

Compound 12 (^{13}C NMR, 101 MHz, $CDCl_3$)

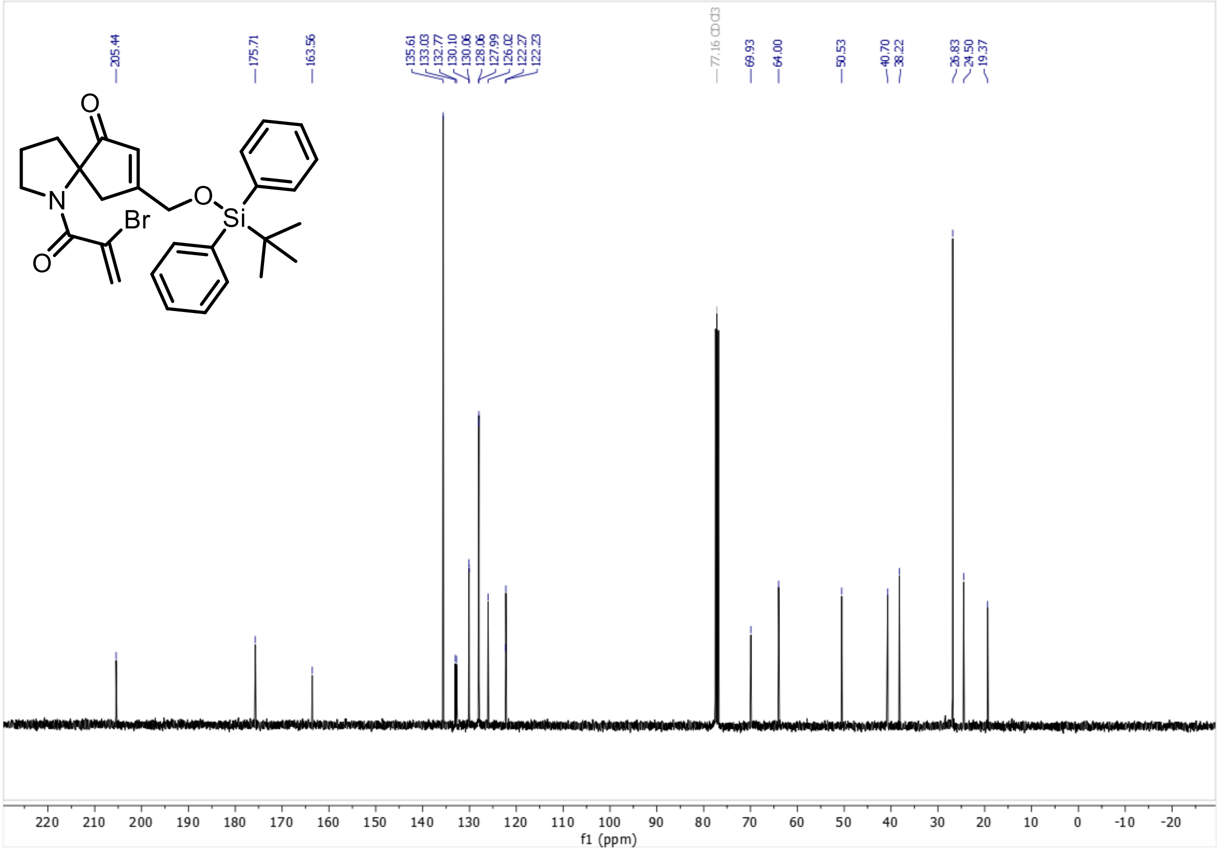

Compound 13 (^{1}H NMR, 500 MHz, $CDCl_3$)

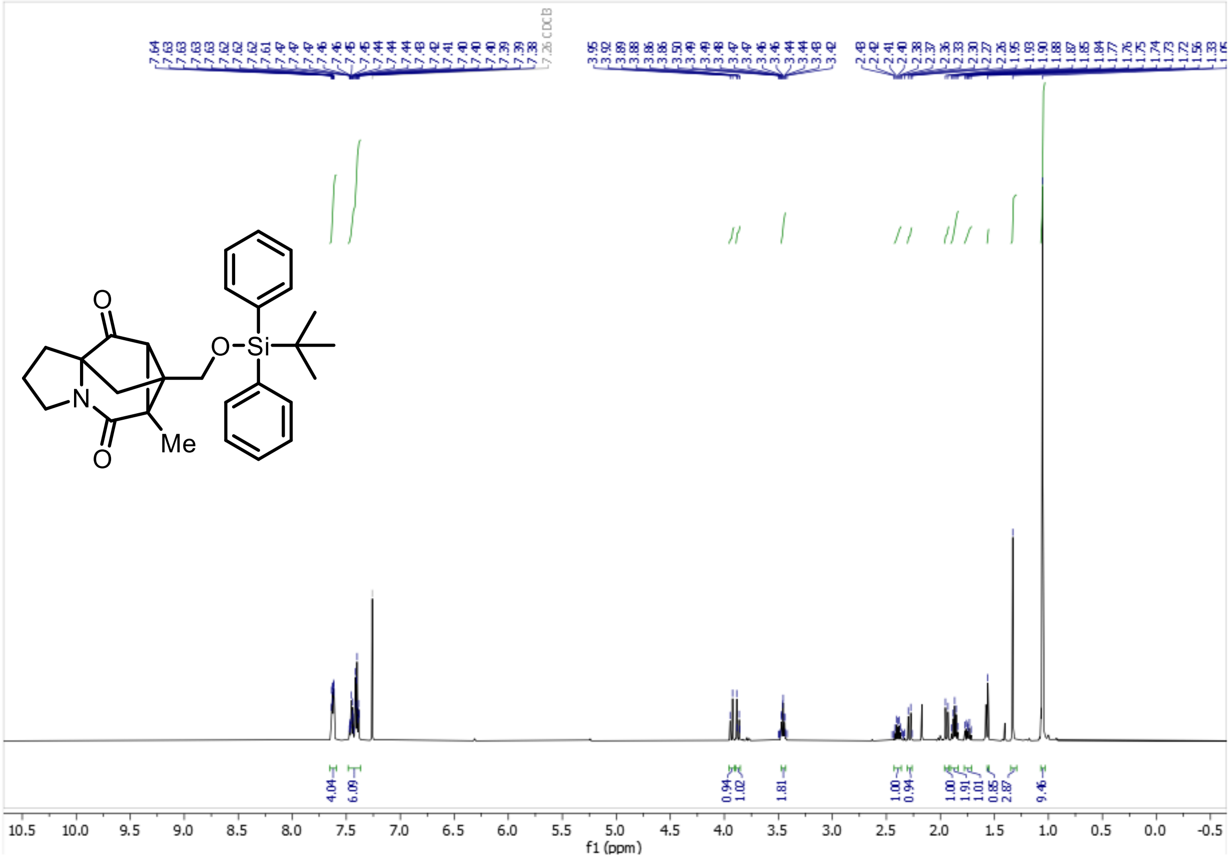

Compound 13 (^{13}C NMR, 126 MHz, $CDCl_3$)

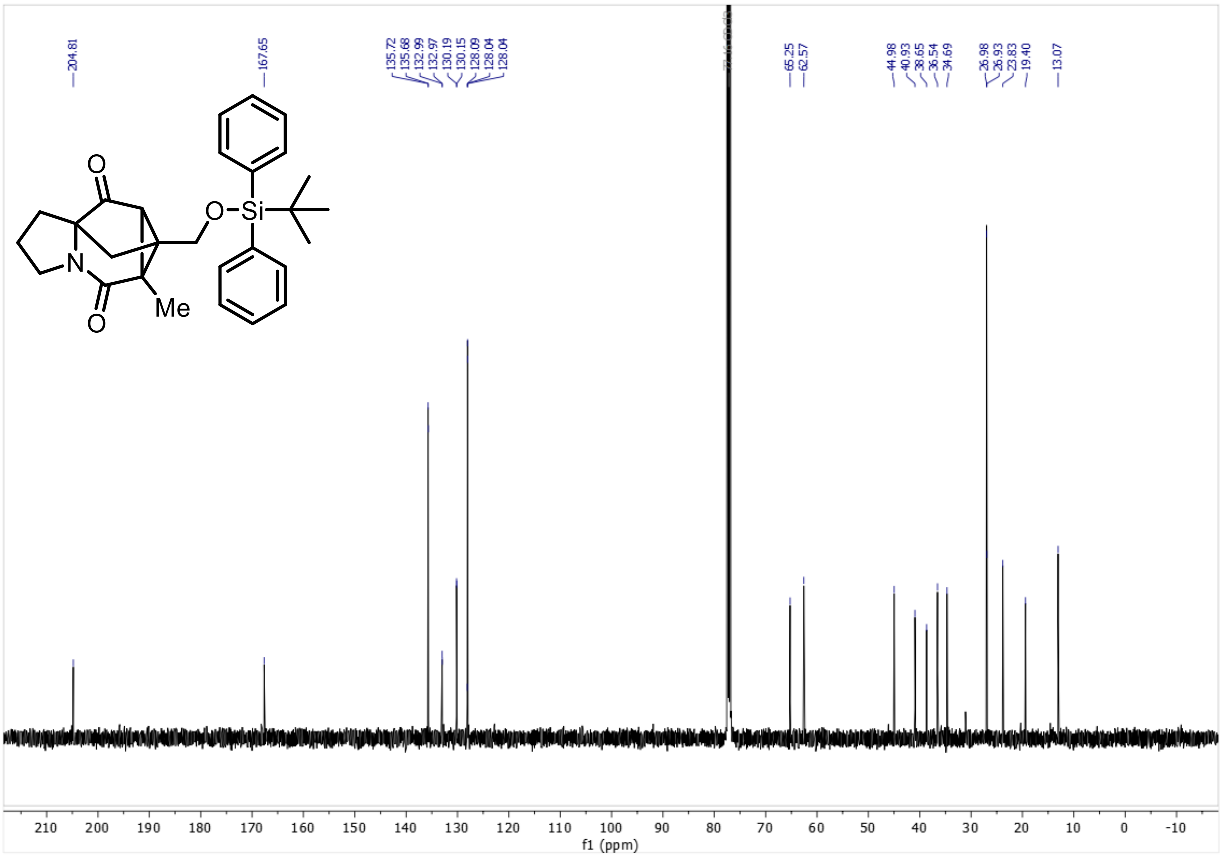

Compound 14 (¹H NMR, 600 MHz, CDCl$_3$)

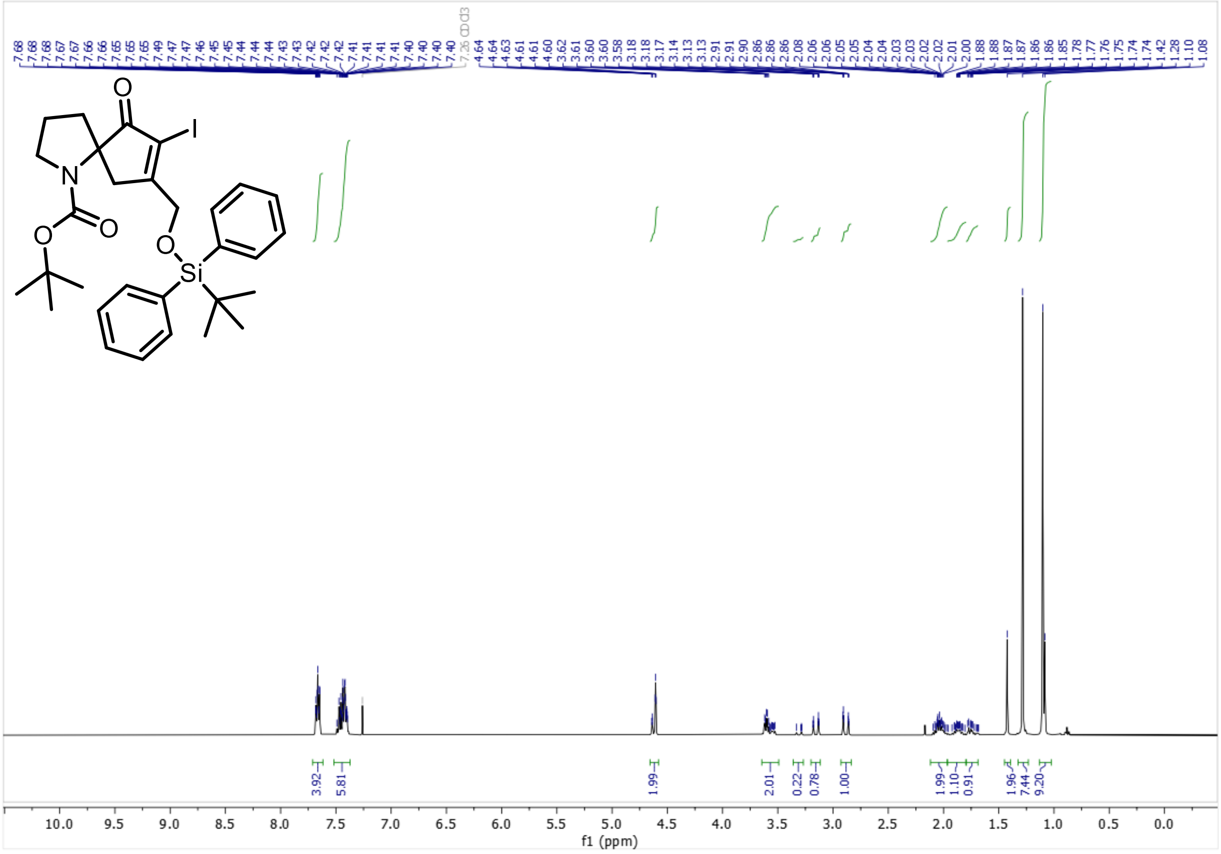

Compound 14 (¹³C NMR, 151 MHz, CDCl$_3$)

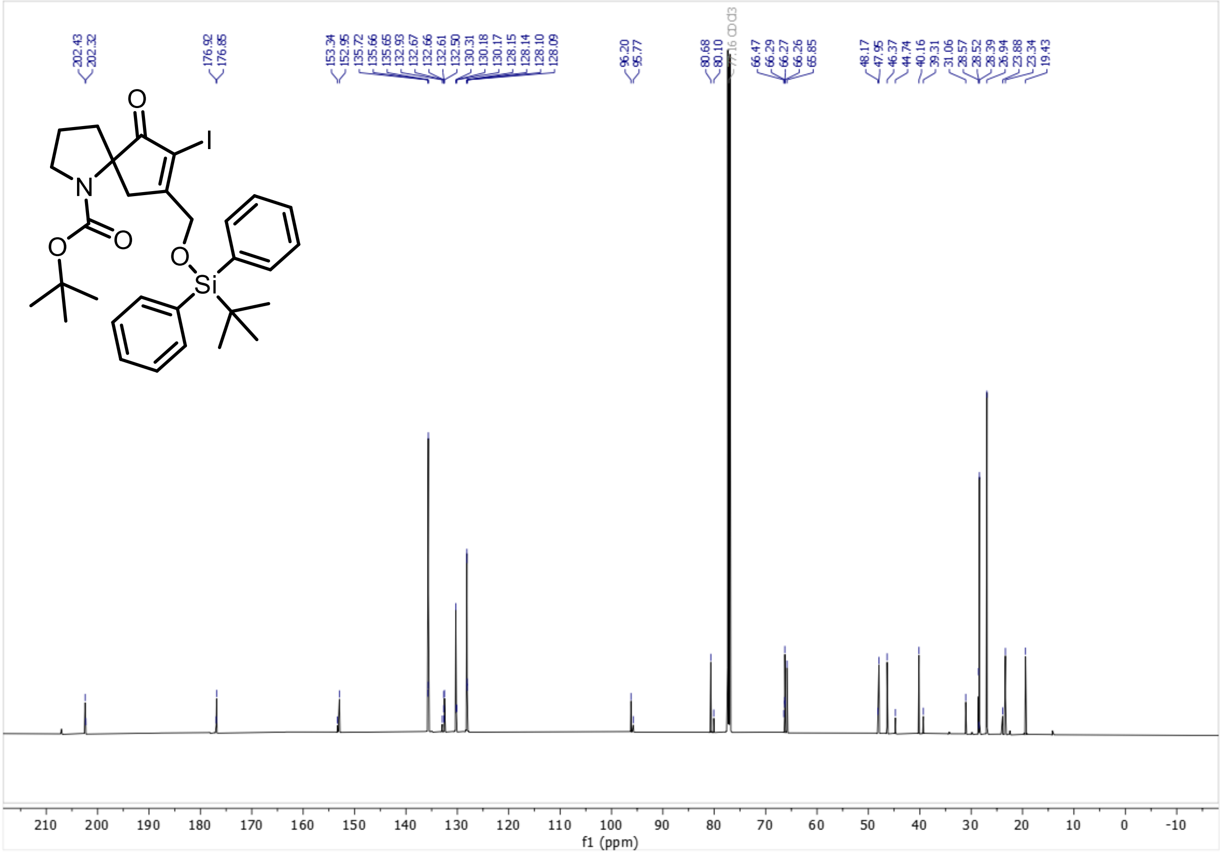

Compound 15 (^{1}H NMR, 600 MHz, CDCl$_3$)

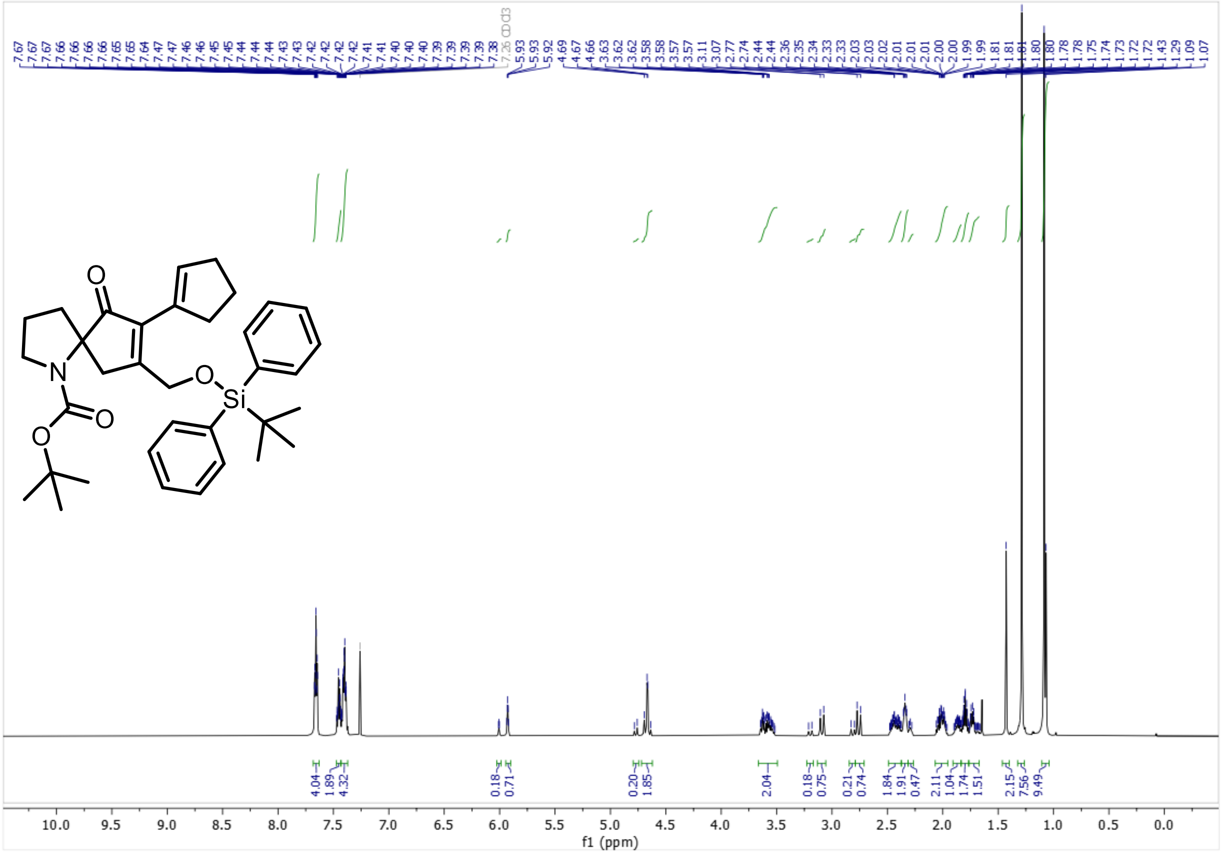

Compound 15 (^{13}C NMR, 151 MHz, CDCl$_3$)

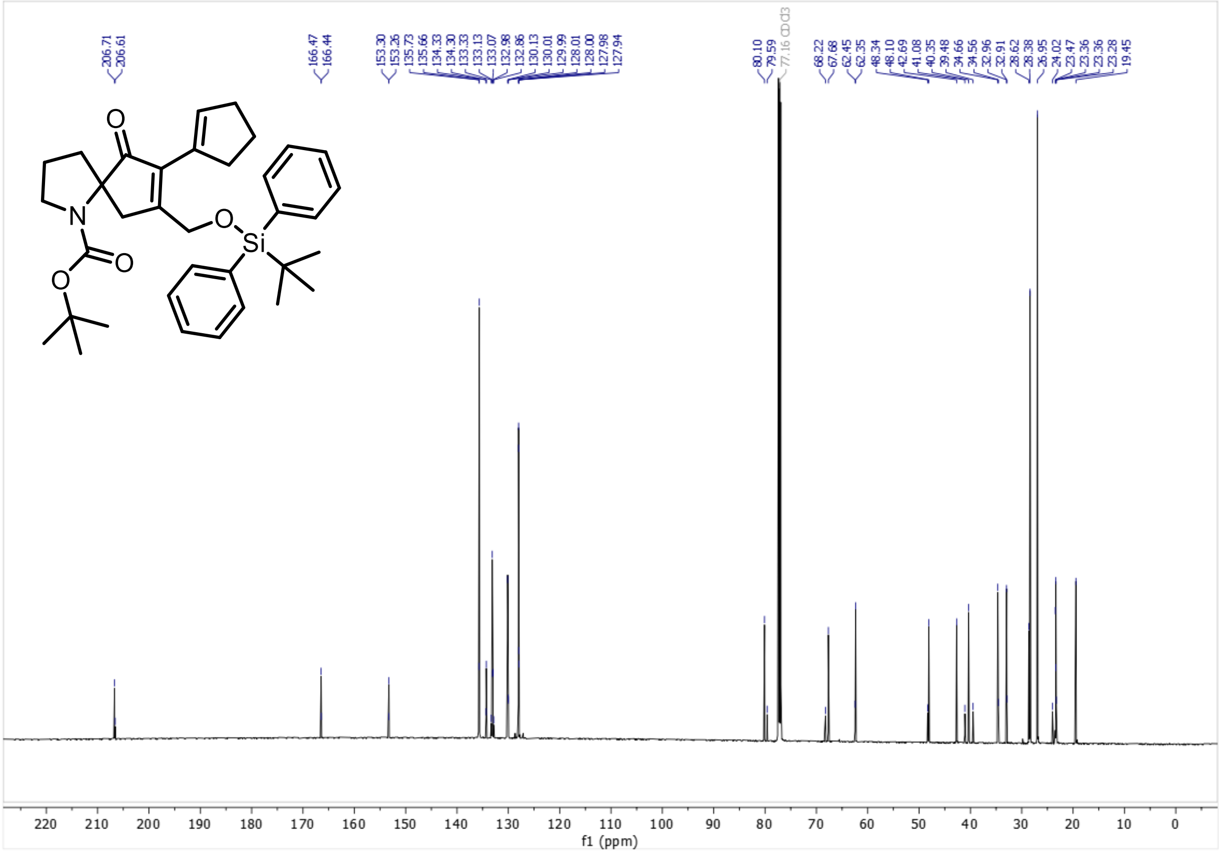

Compound 16 (¹H NMR, 400 MHz, CDCl$_3$)

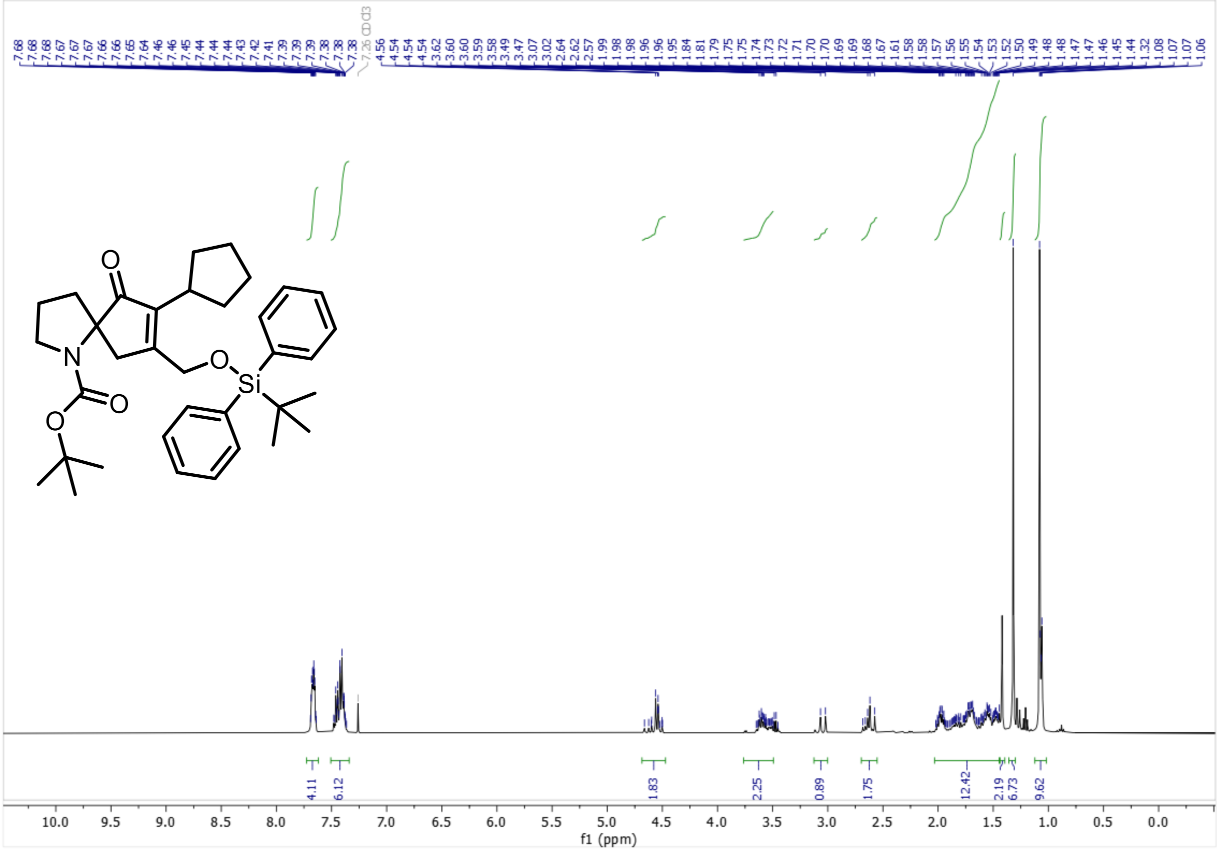

Compound 16 (^{13}C NMR, 101 MHz, CDCl$_3$)

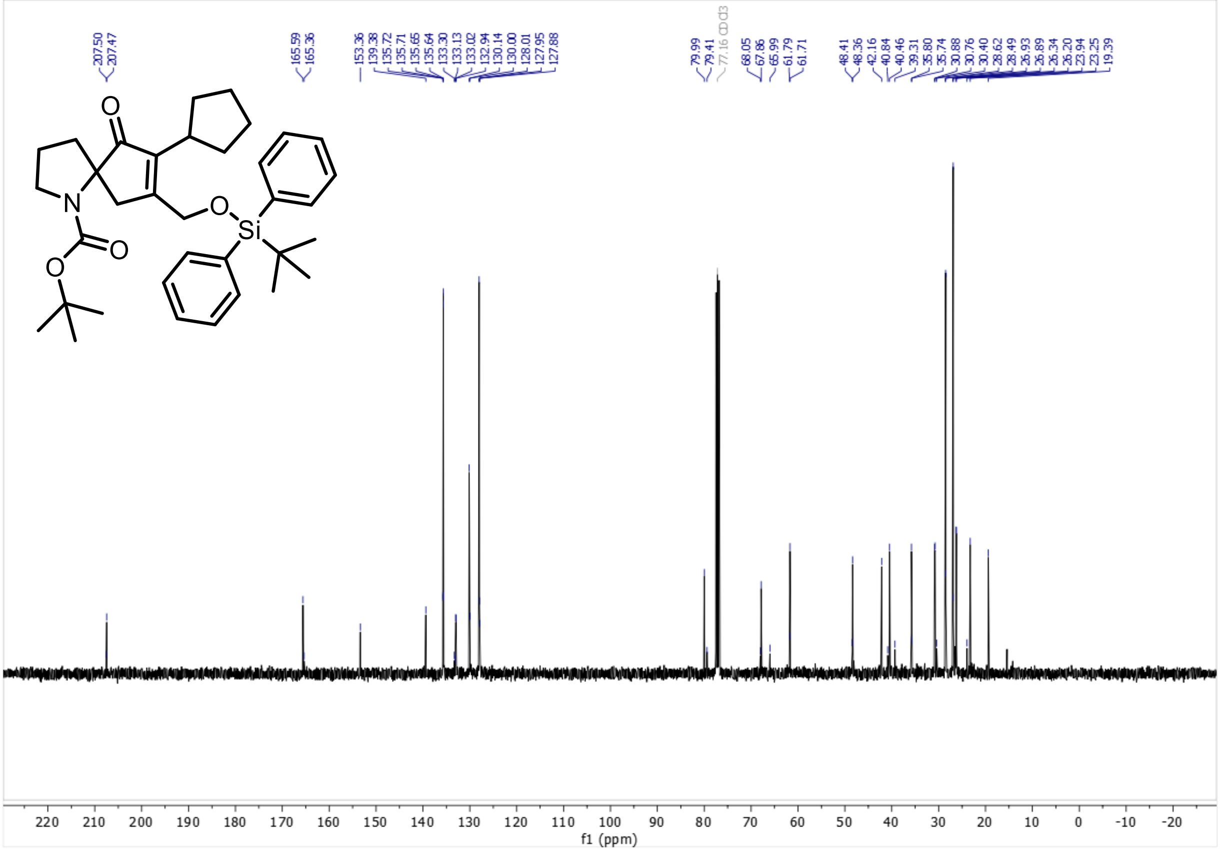

Compound 17 (¹H NMR, 600 MHz, $CDCl_3$)

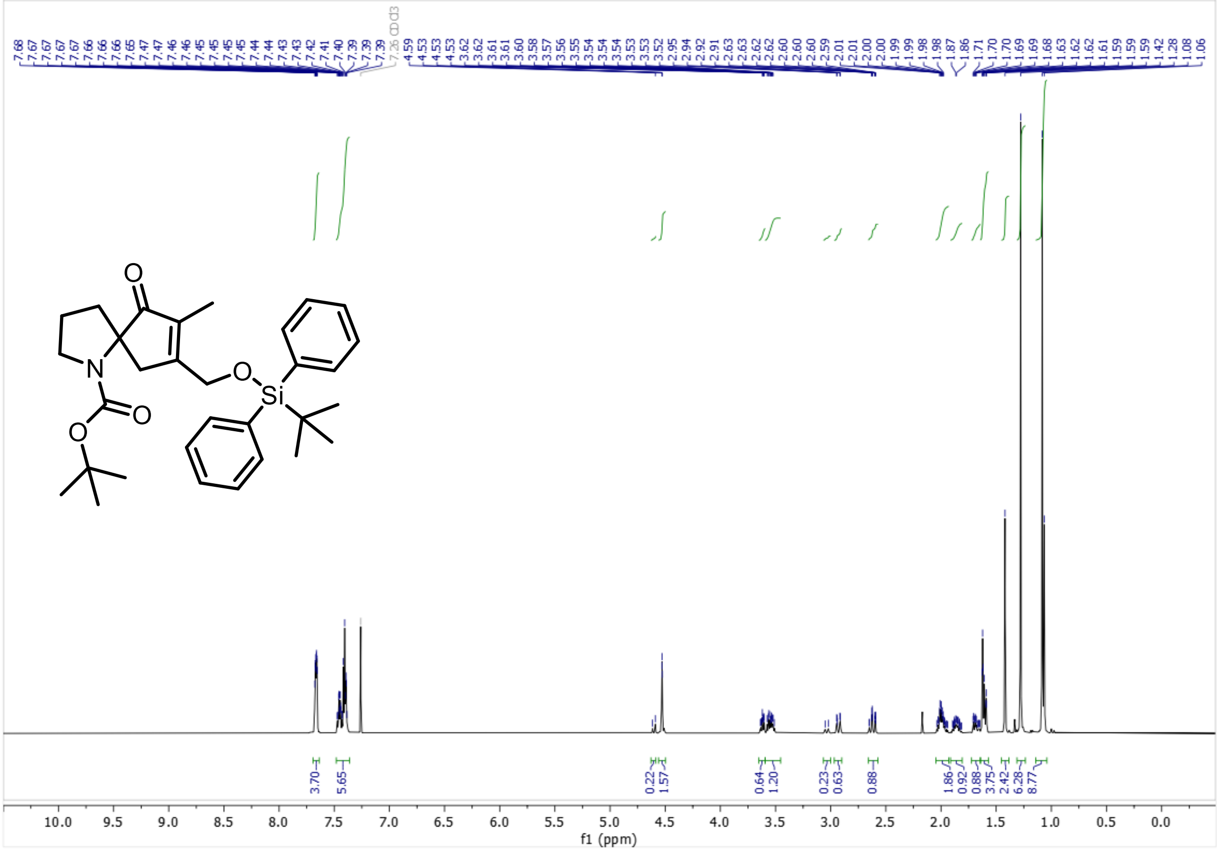

Compound 17 (¹³C NMR, 151 MHz, $CDCl_3$)

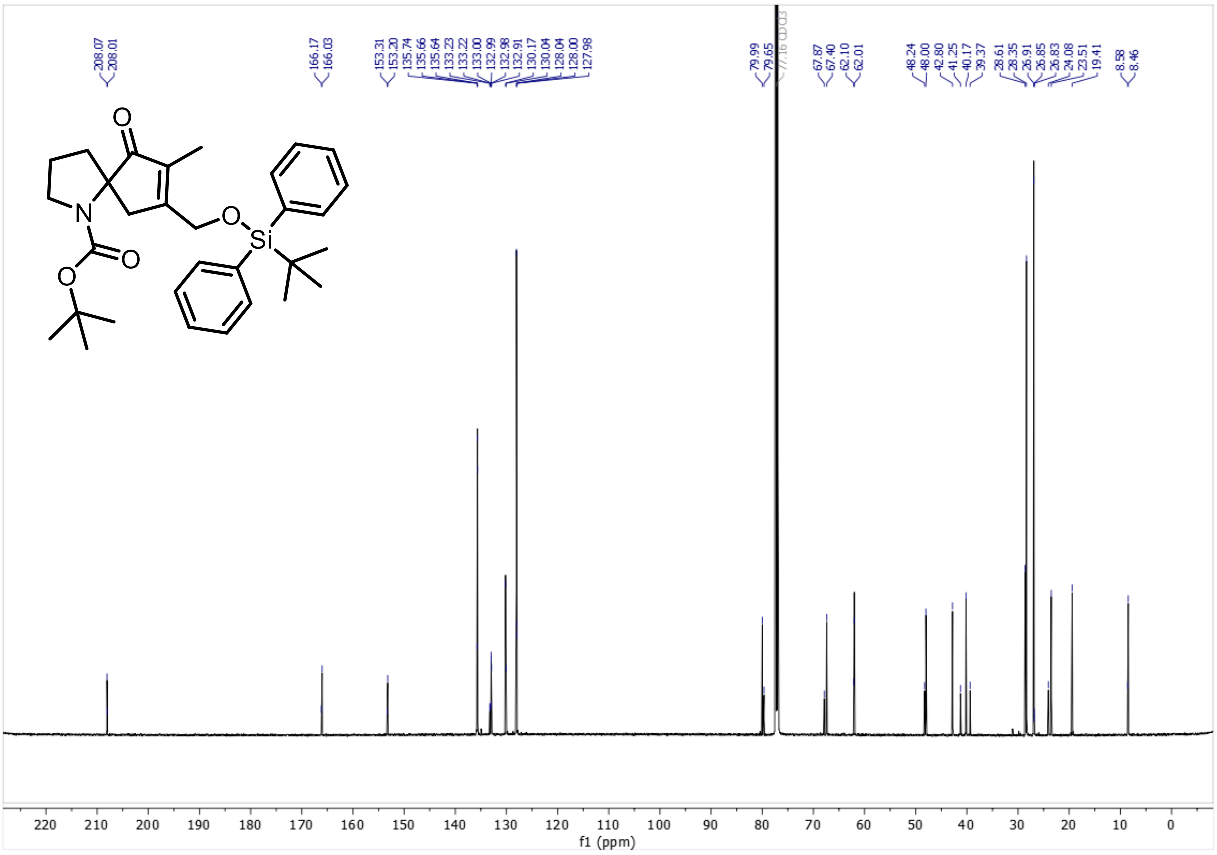

Compound 18 (^{1}H NMR, 400 MHz, $CDCl_3$)

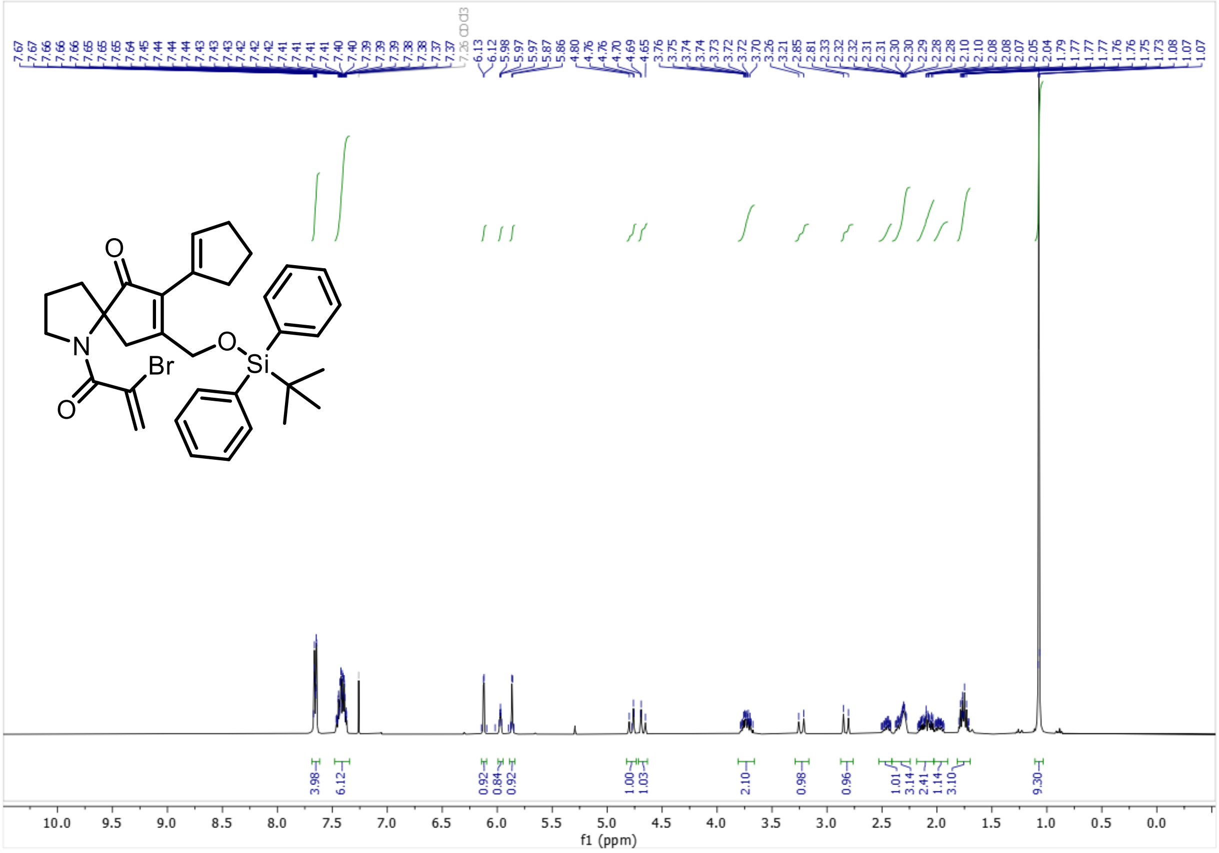

Compound 18 (^{13}C NMR, 101 MHz, $CDCl_3$)

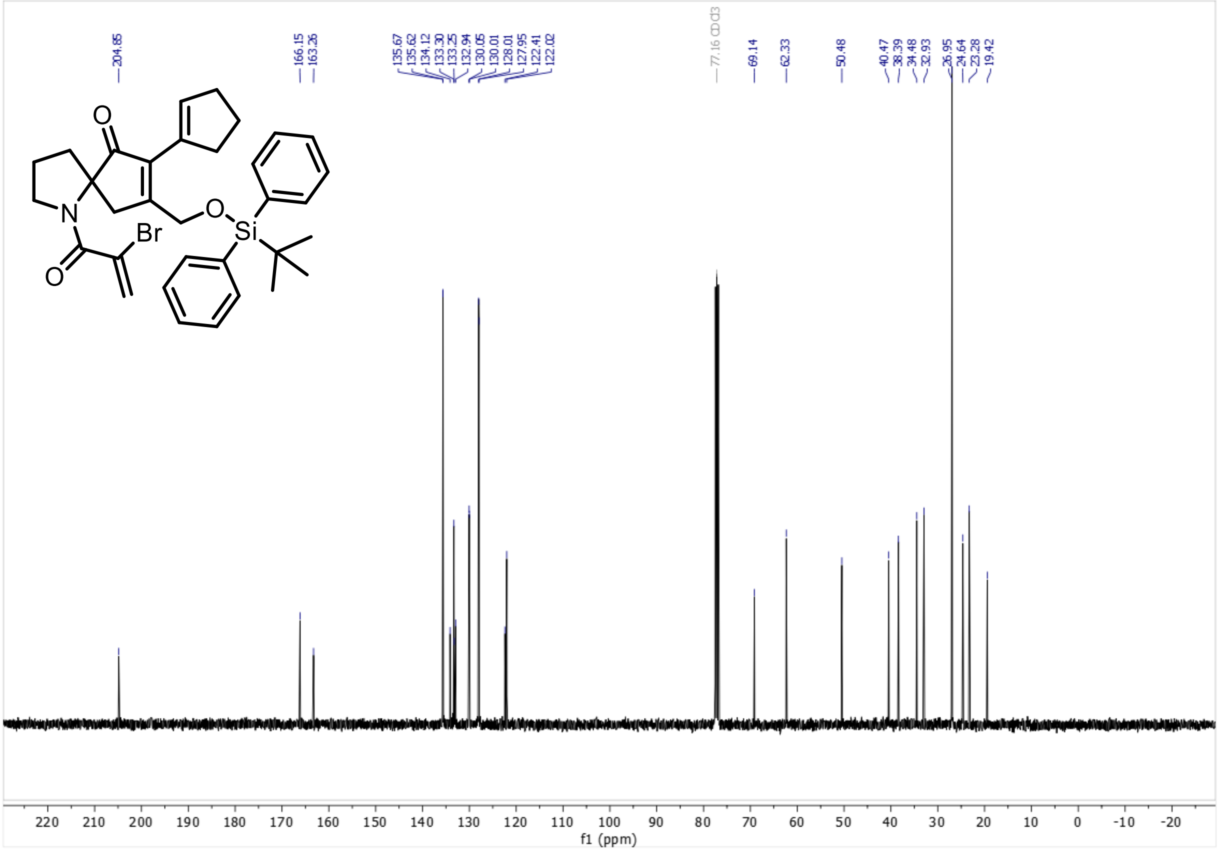

Compound 19 (¹H NMR, 400 MHz, CDCl₃)

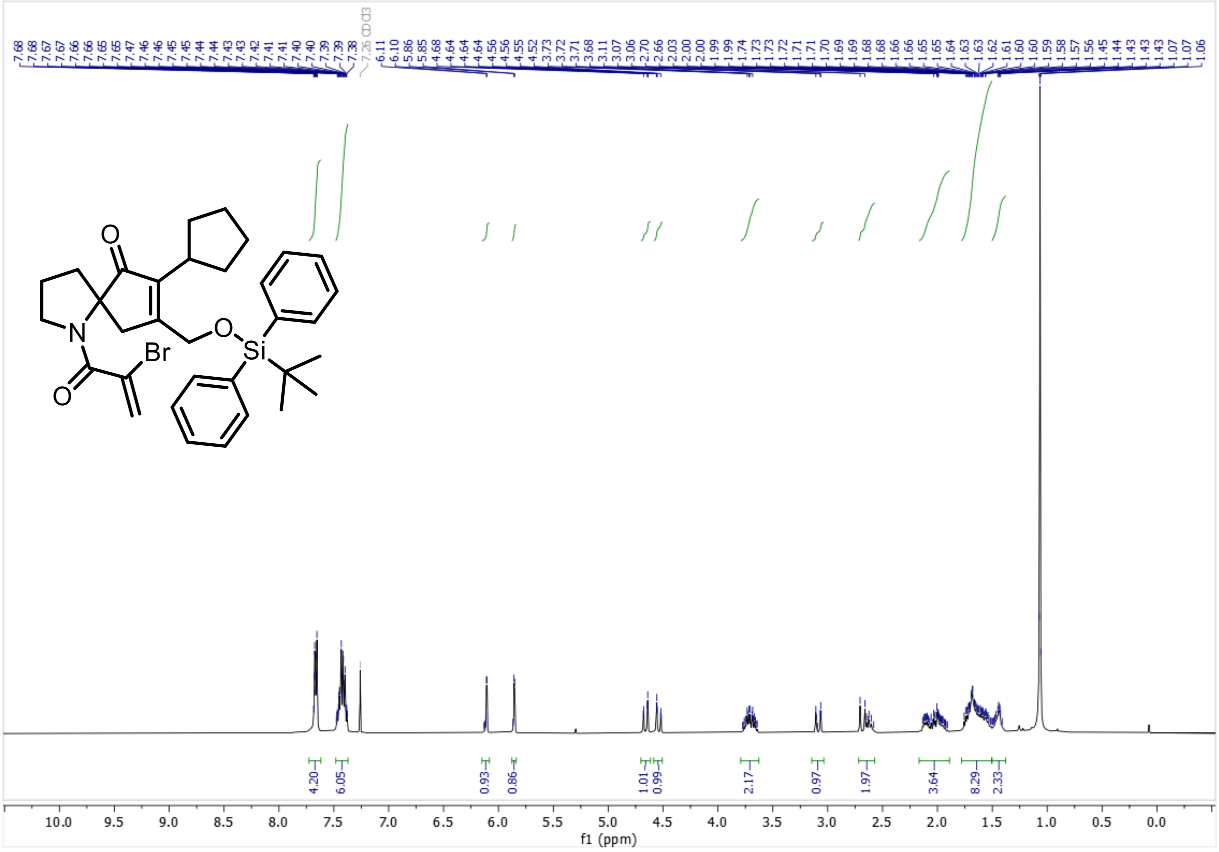

Compound 19 (¹³C NMR, 101 MHz, CDCl₃)

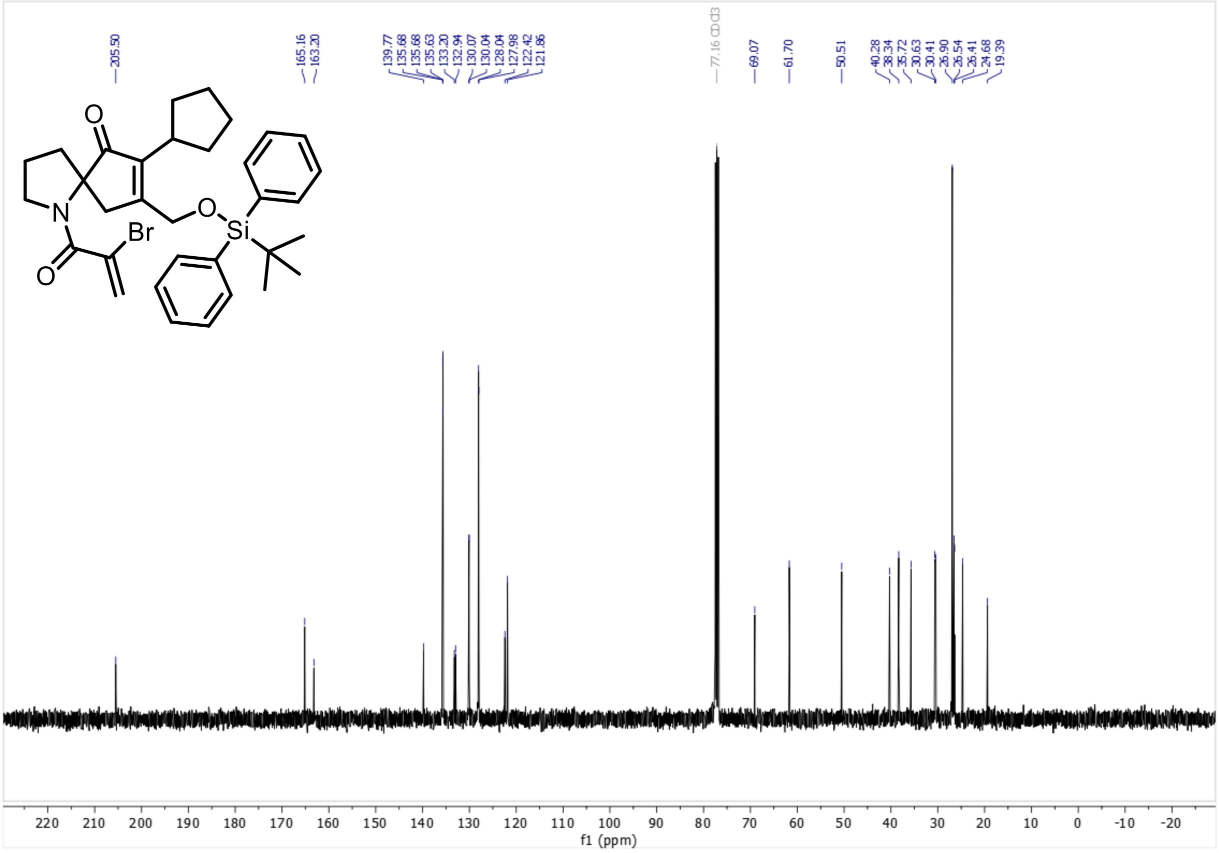

Compound 20 (¹H NMR, 400 MHz, $CDCl_3$)

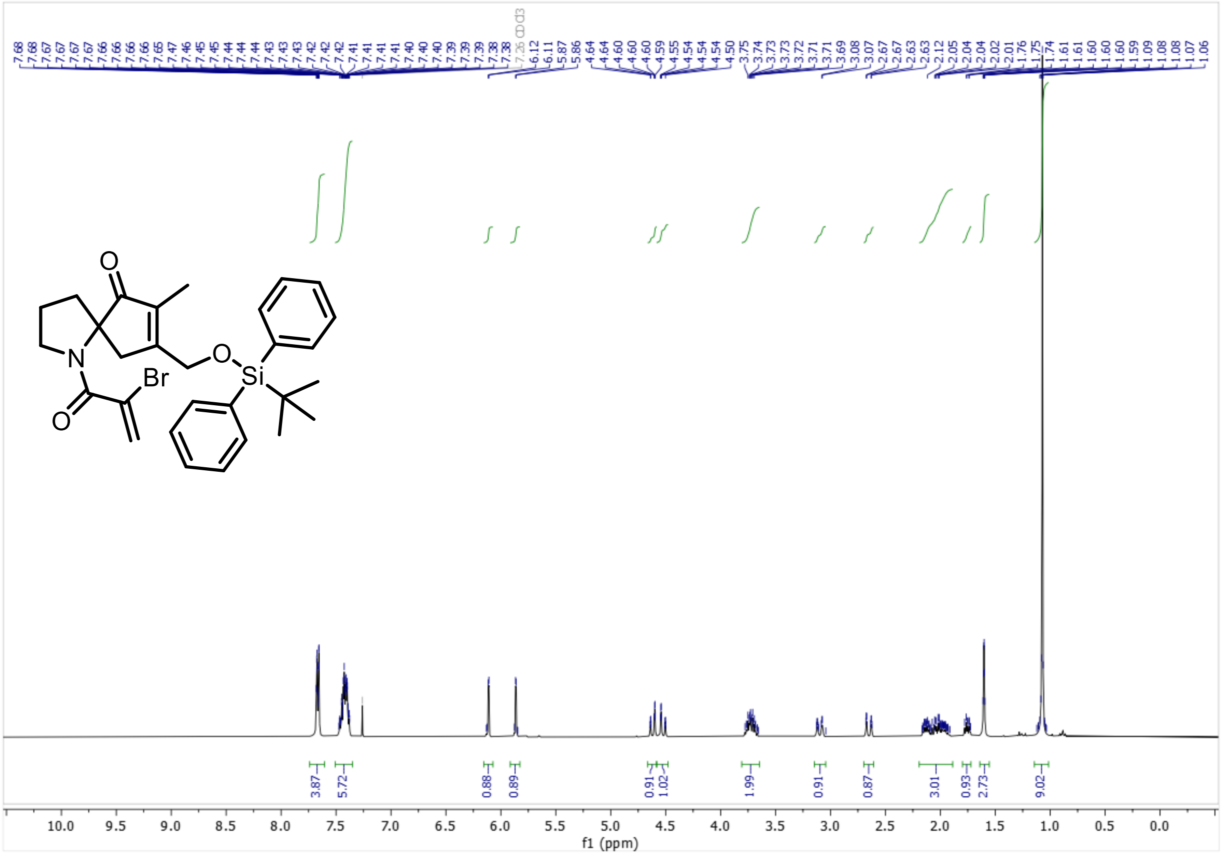

Compound 20 (¹³C NMR, 101 MHz, $CDCl_3$)

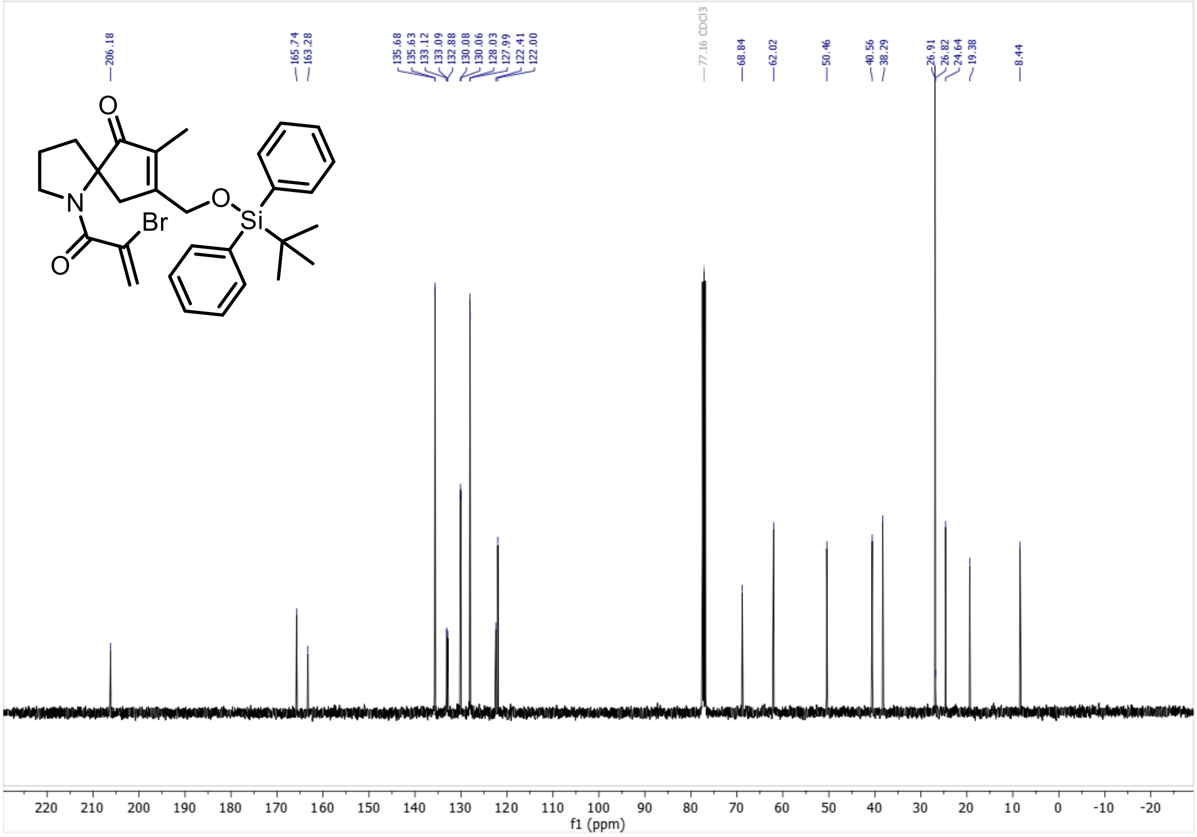

Compound 21 (^{1}H NMR, 400 MHz, CDCl$_3$)

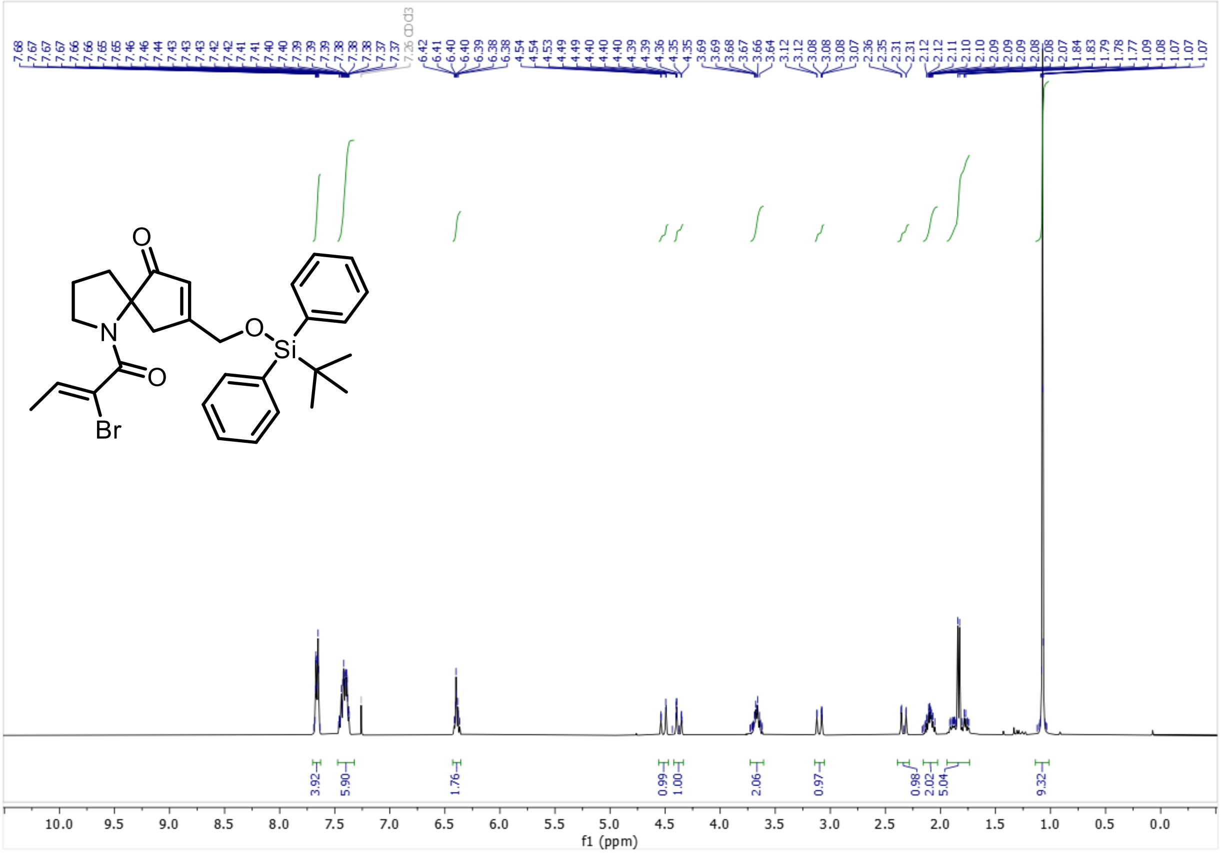

Compound 21 (^{13}C NMR, 101 MHz, CDCl$_3$)

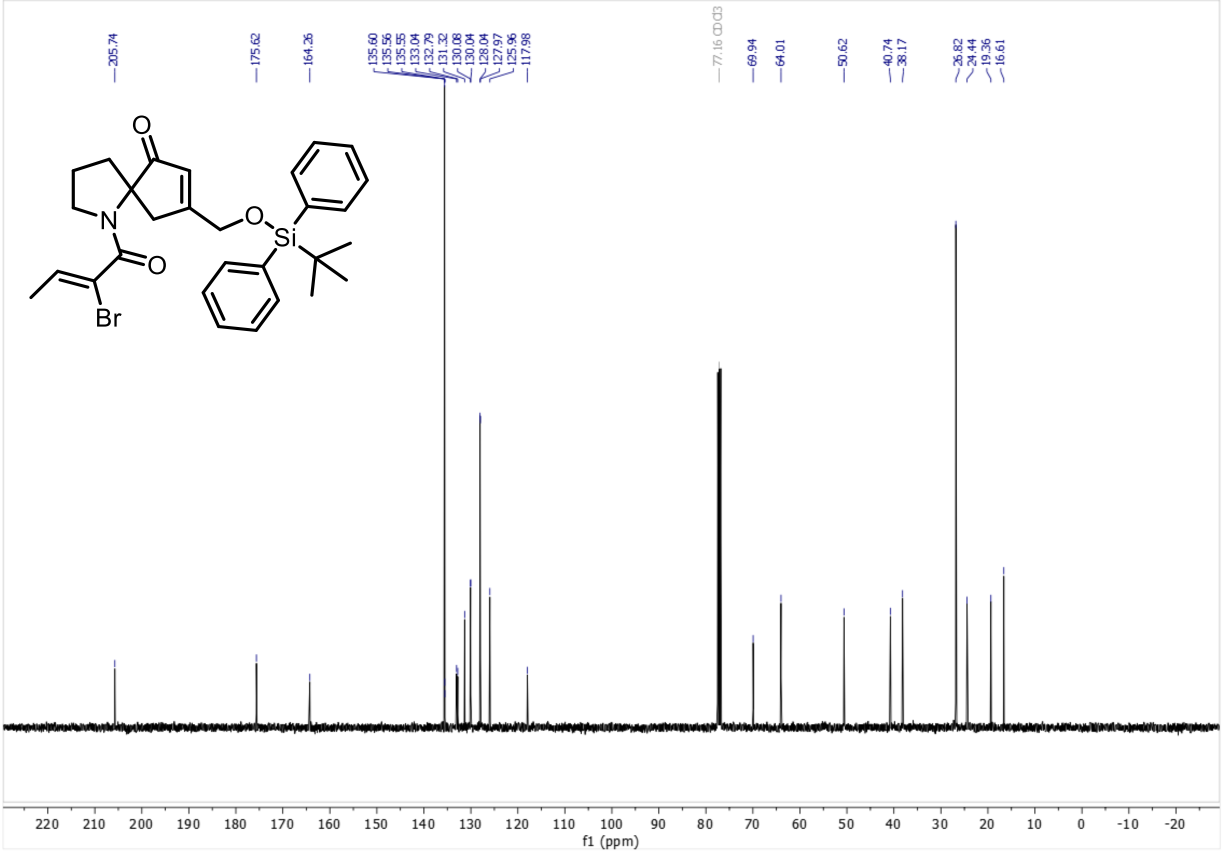

Compound 23 (^{1}H NMR, 600 MHz, $CDCl_3$)

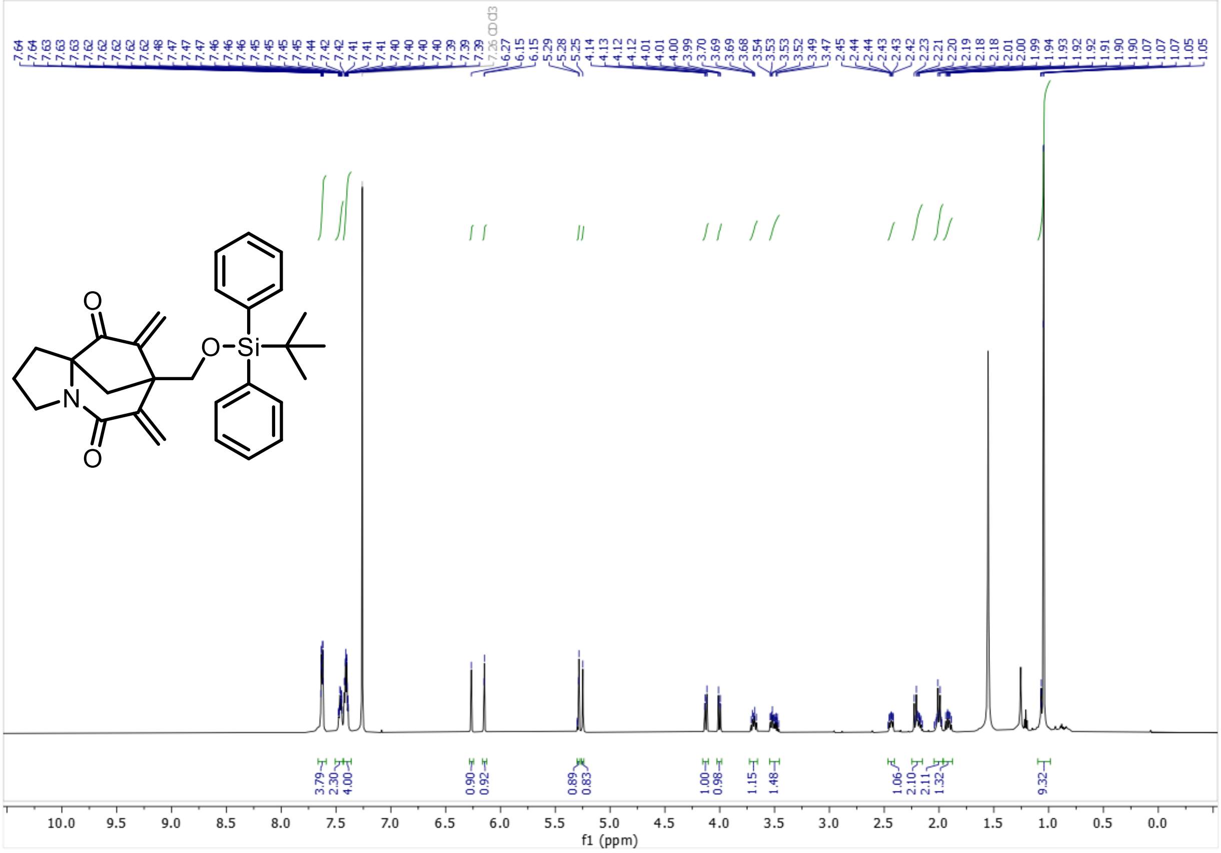

Compound 23 (^{13}C NMR, 151 MHz, $CDCl_3$)

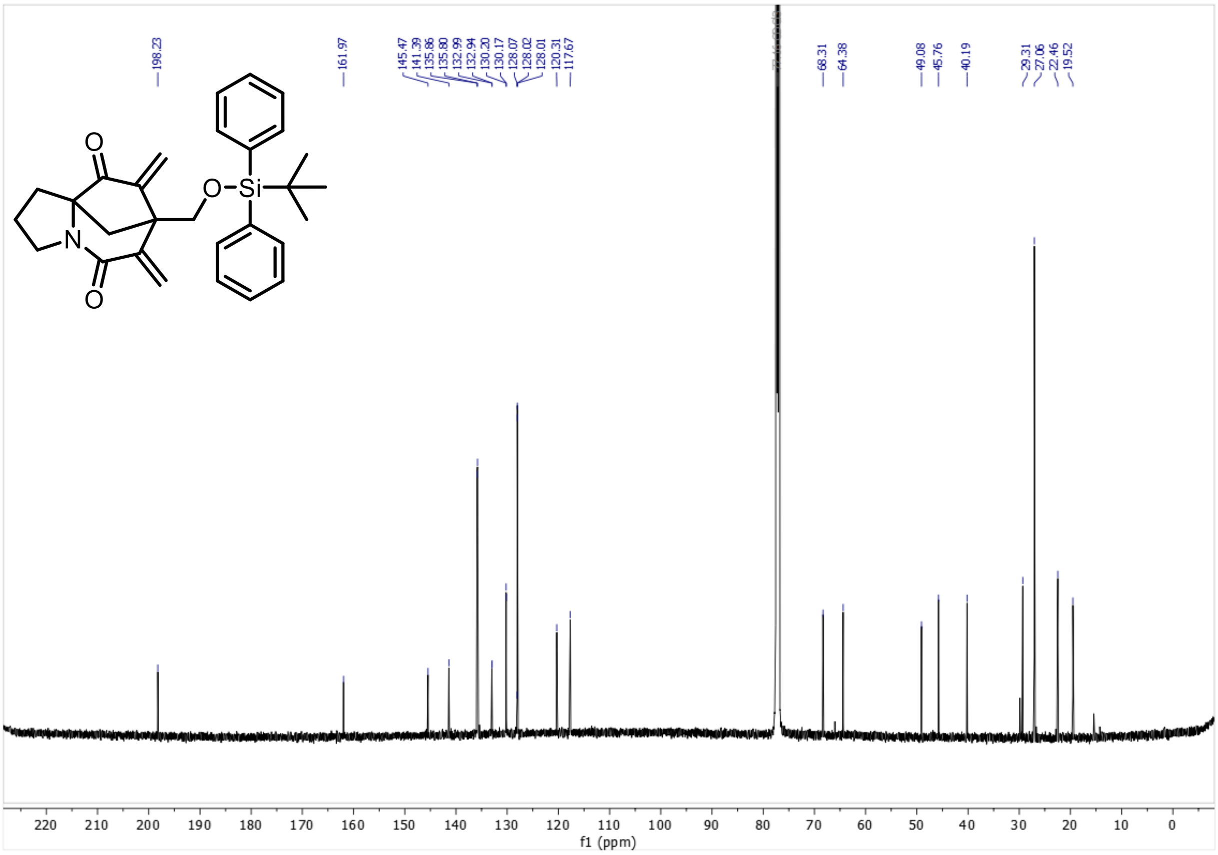

__Compound 24 (^{1}H NMR, 600 MHz, CDCl$_3$)__

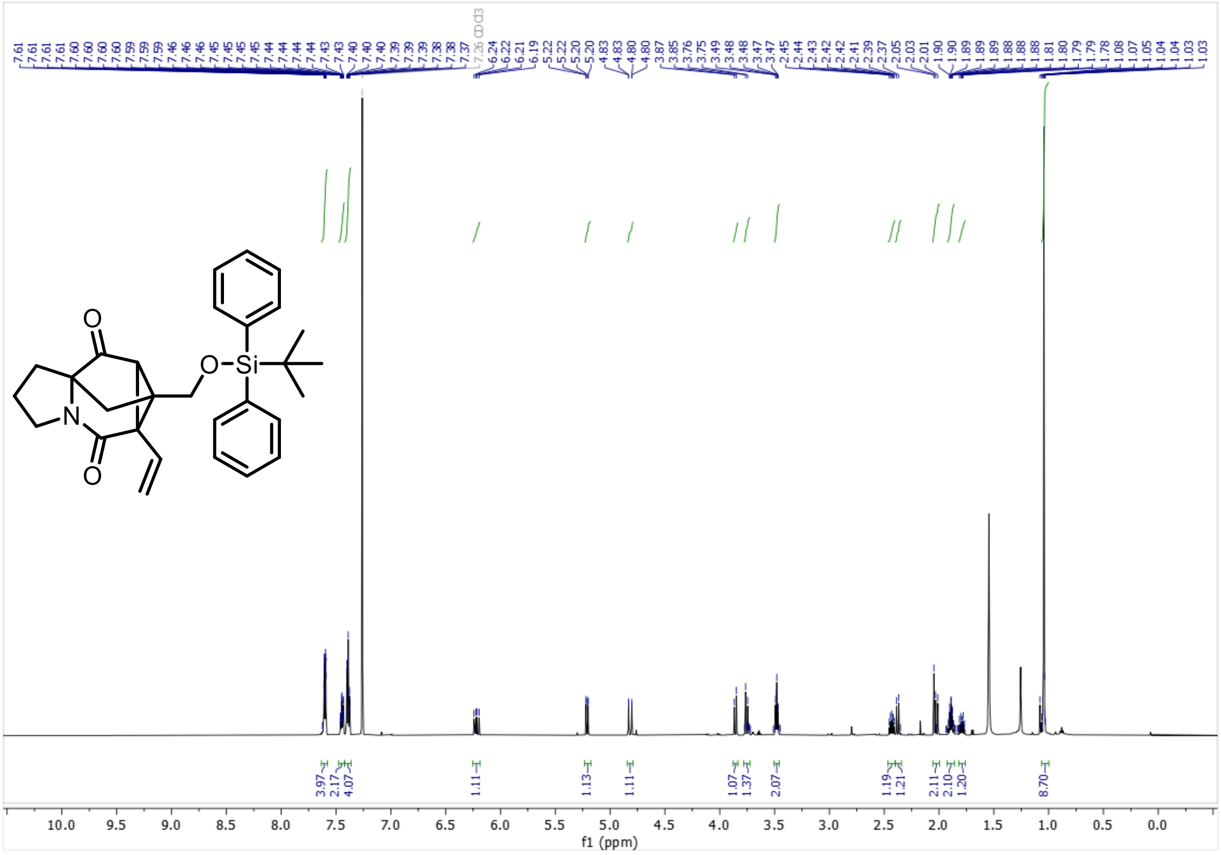

__Compound 24 (^{13}C NMR, 151 MHz, CDCl$_3$)__

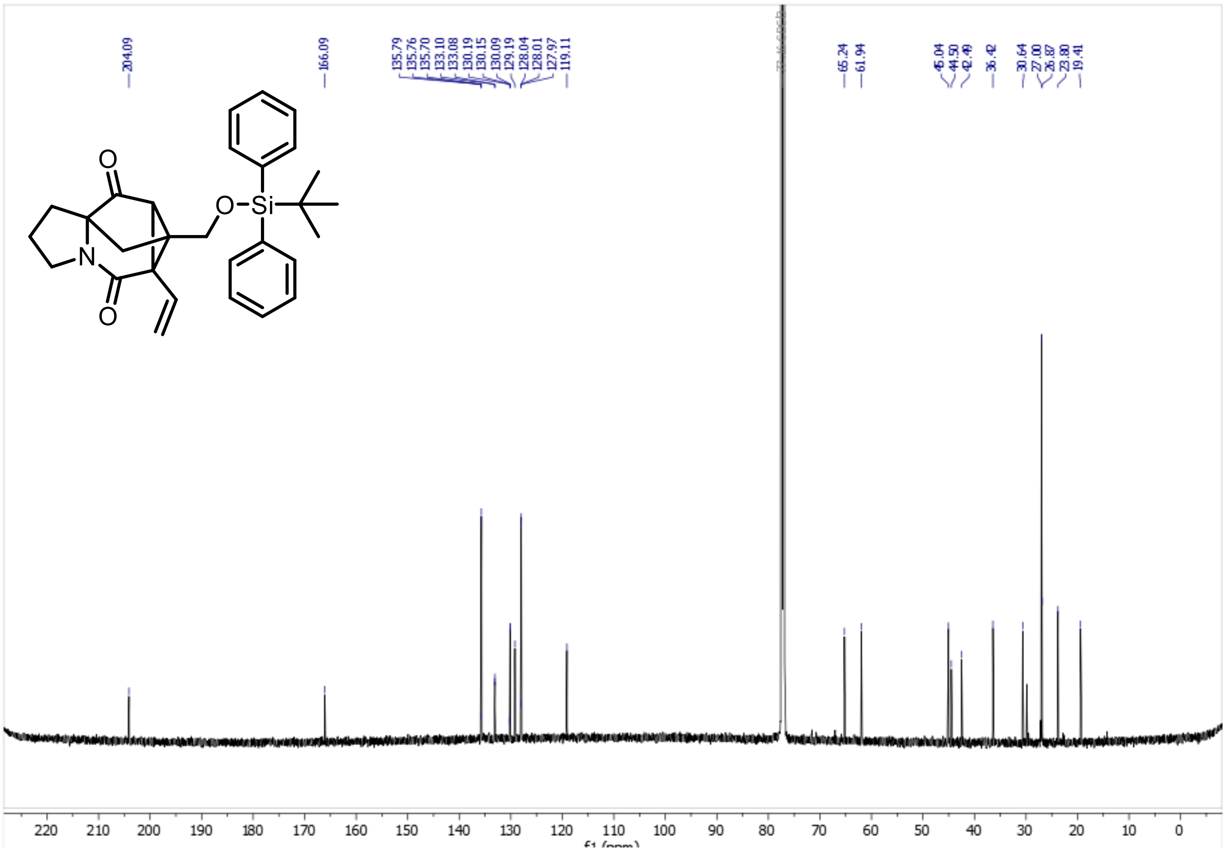

Compound 4 (^1H NMR, 600 MHz, $CDCl_3$)

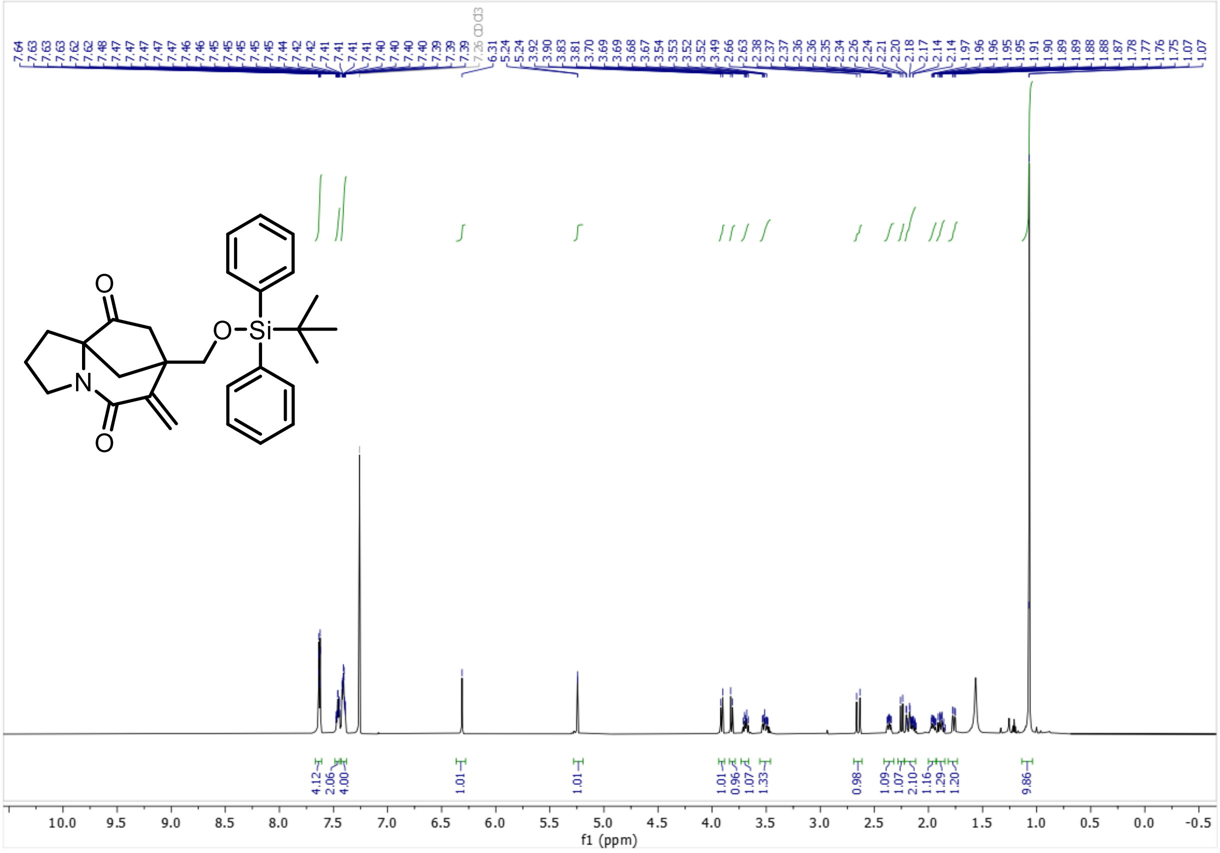

Compound 4 (^{13}C NMR, 151 MHz, $CDCl_3$)

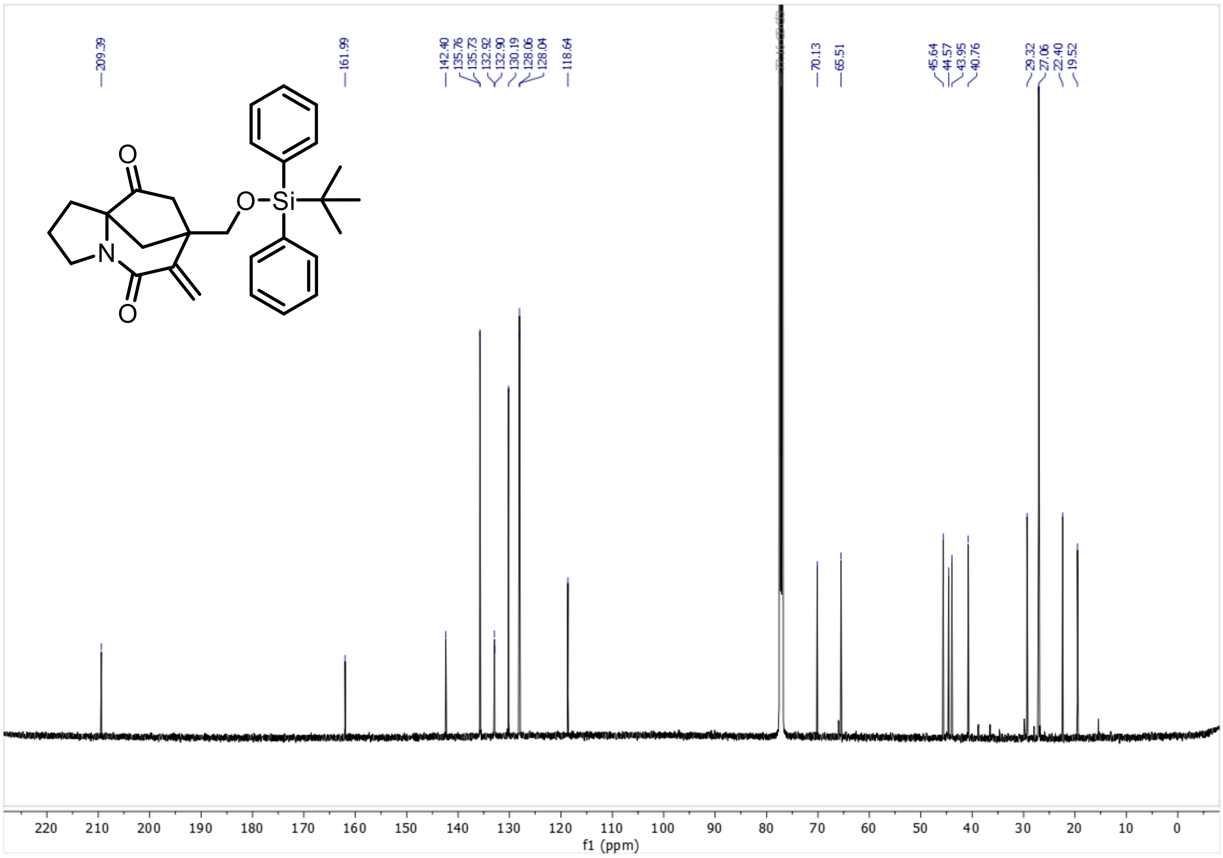

Compound 26 (^{1}H NMR, 600 MHz, CDCl$_3$)

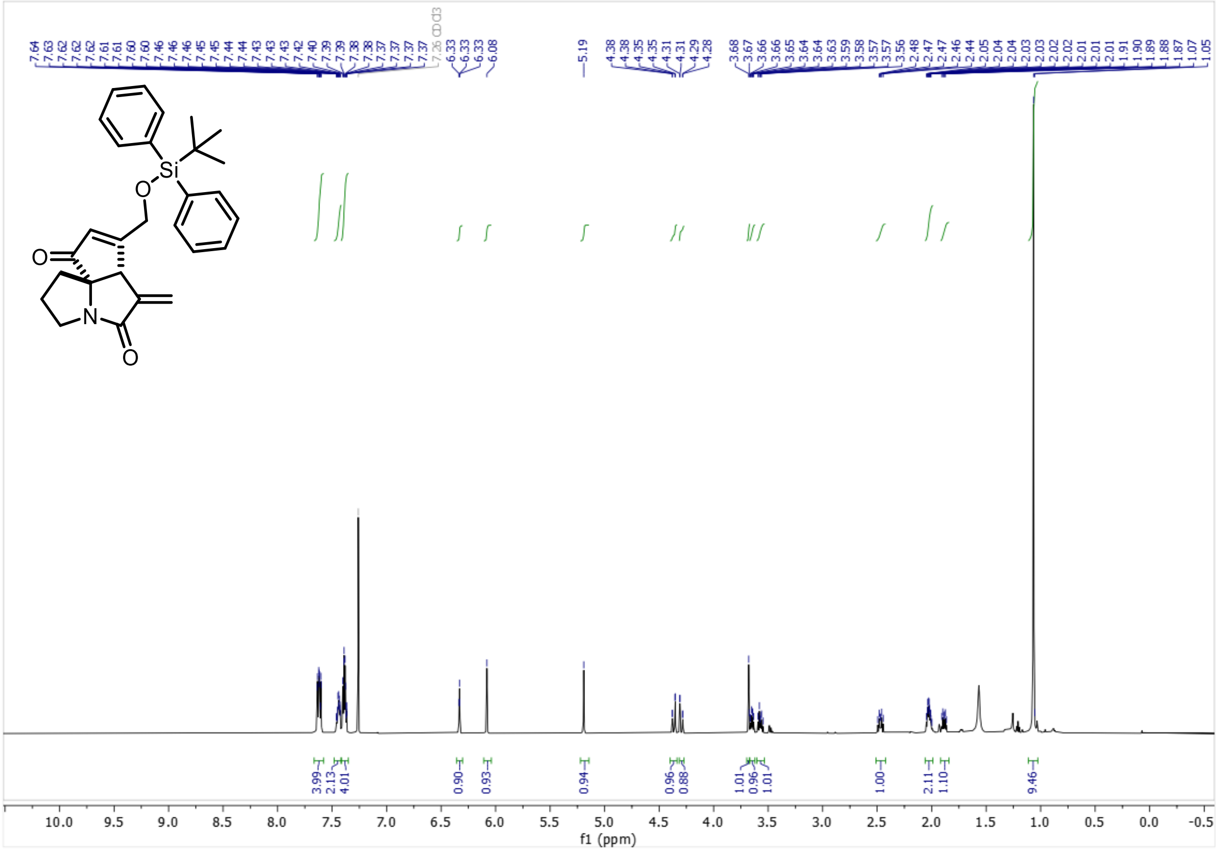

Compound 26 (^{13}C NMR, 151 MHz, CDCl$_3$)

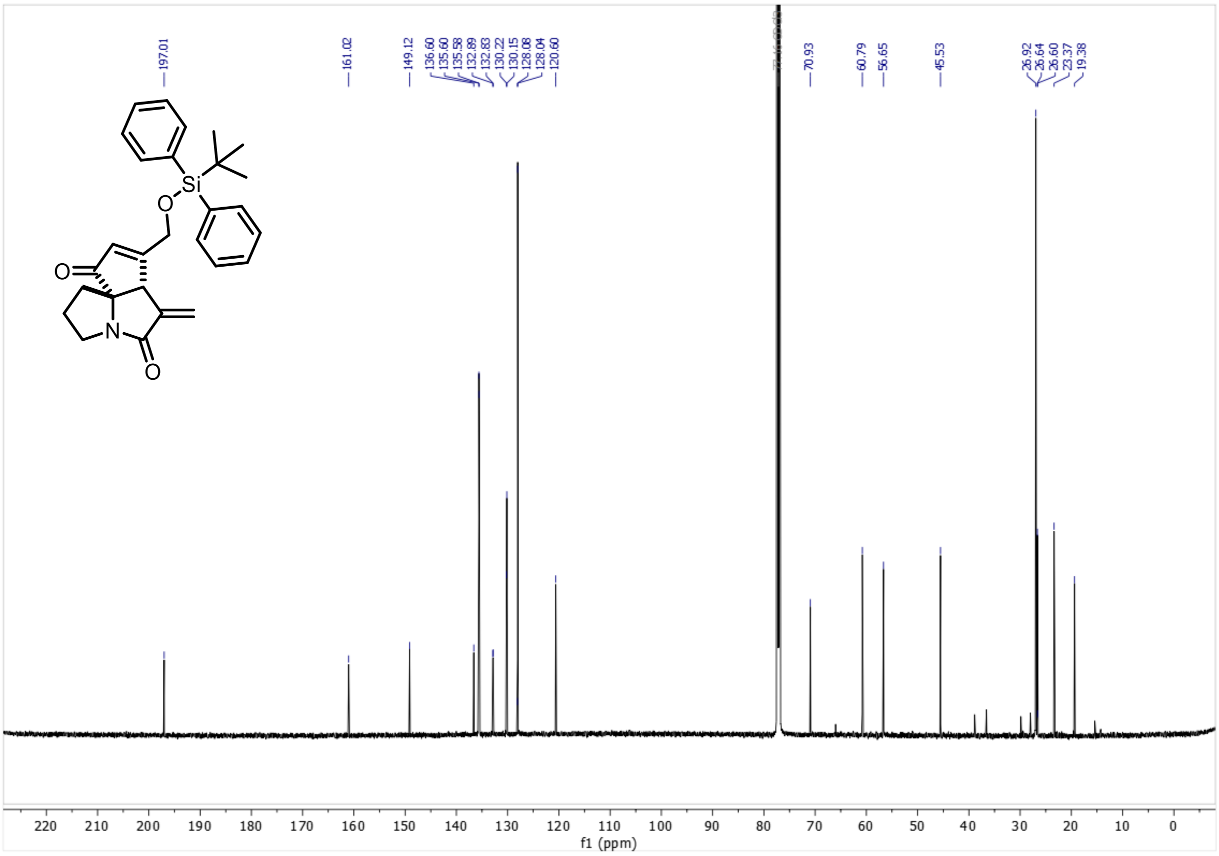

Compound 27 (^{1}H NMR, 600 MHz, $CDCl_3$)

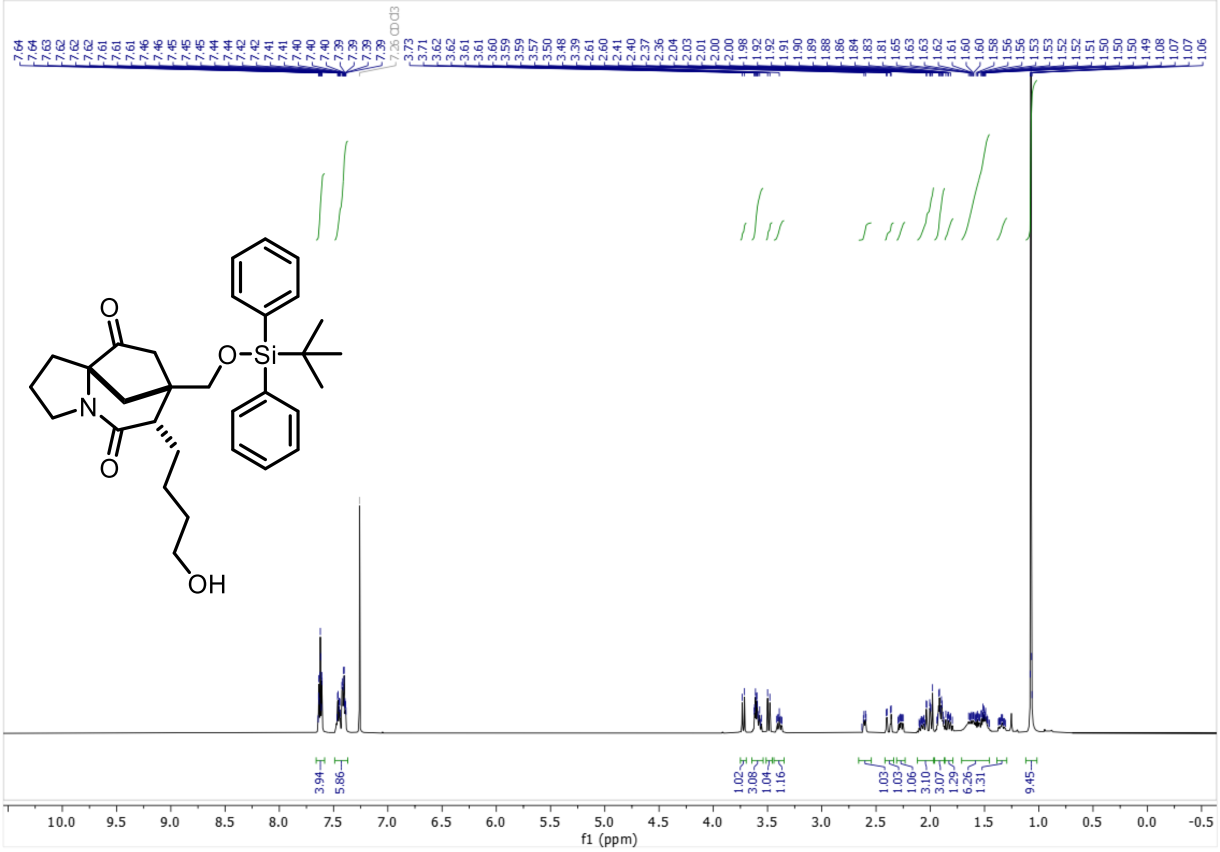

Compound 27 (^{13}C NMR, 126 MHz, $CDCl_3$)

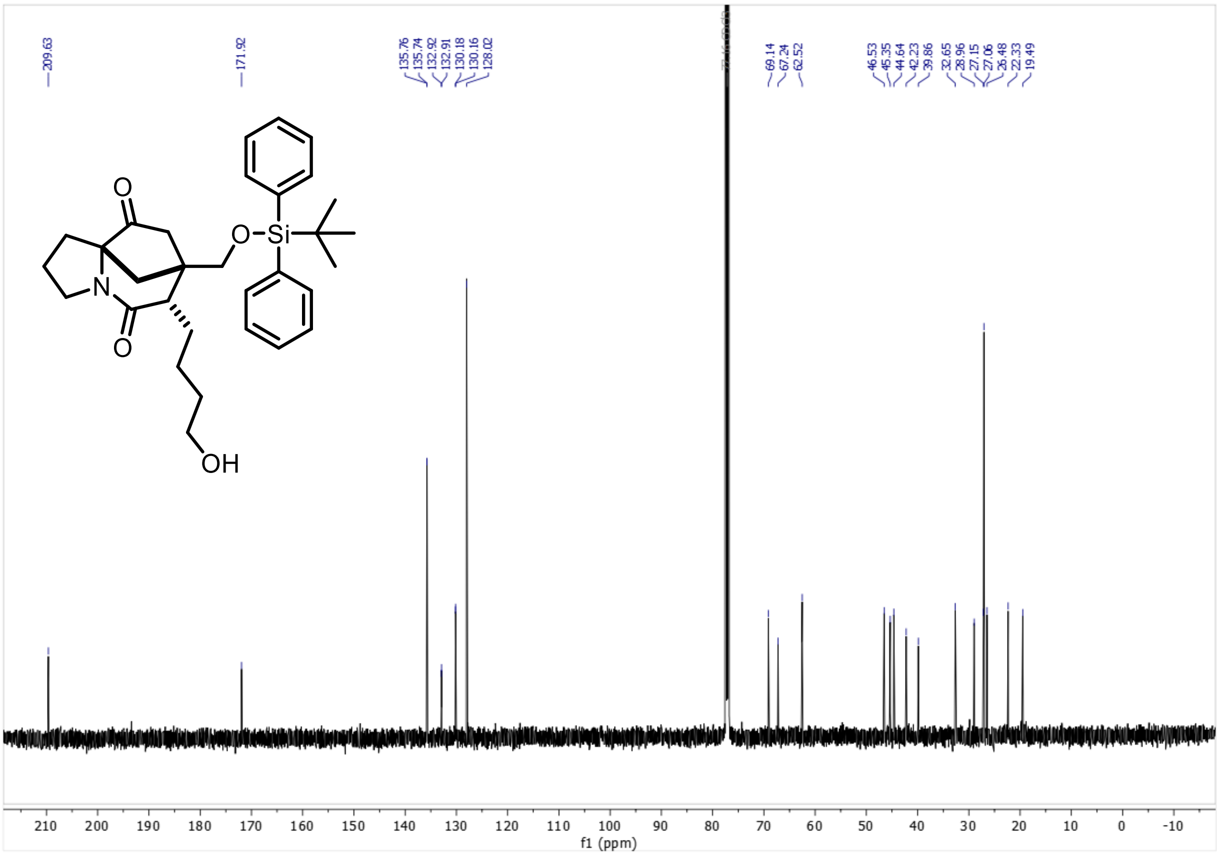

Compound 29 (^1H NMR, 600 MHz, $CDCl_3$)

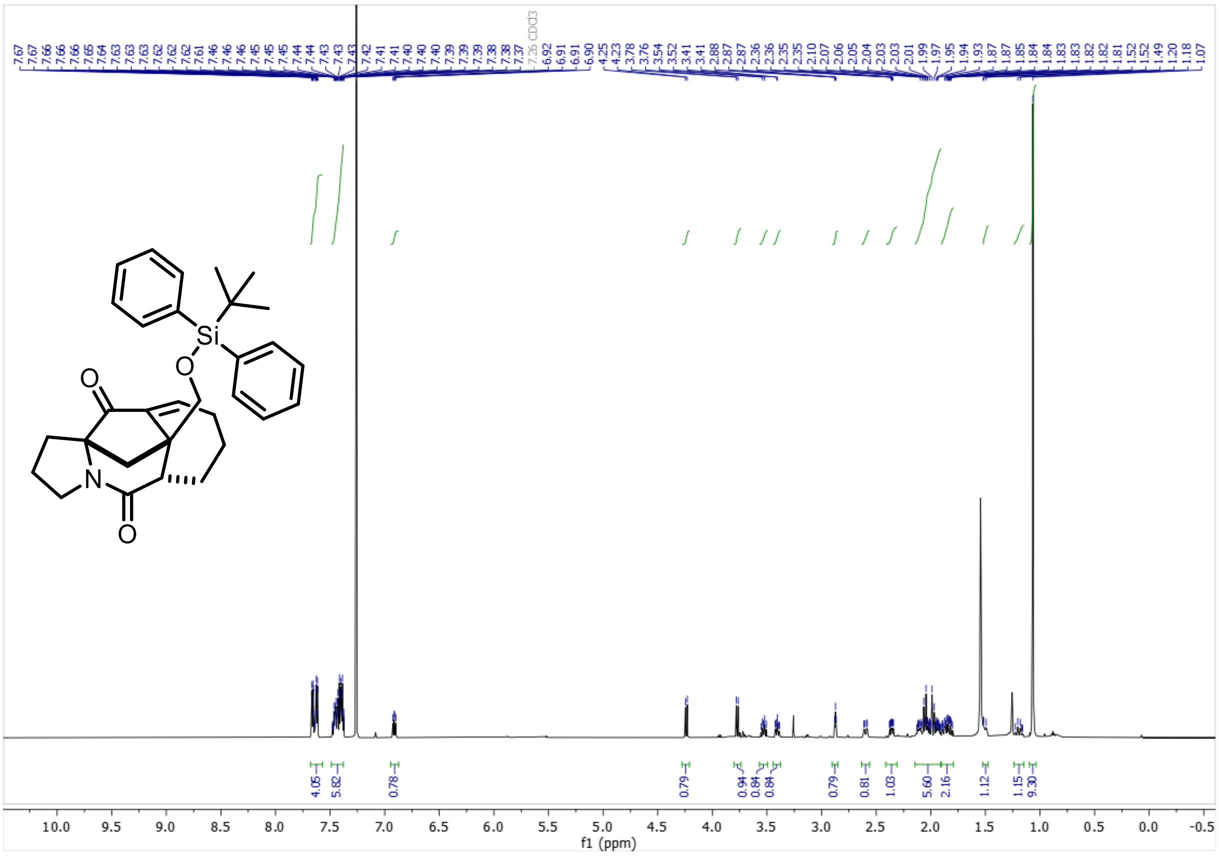

Compound 29 (^{13}C NMR, 151 MHz, $CDCl_3$)

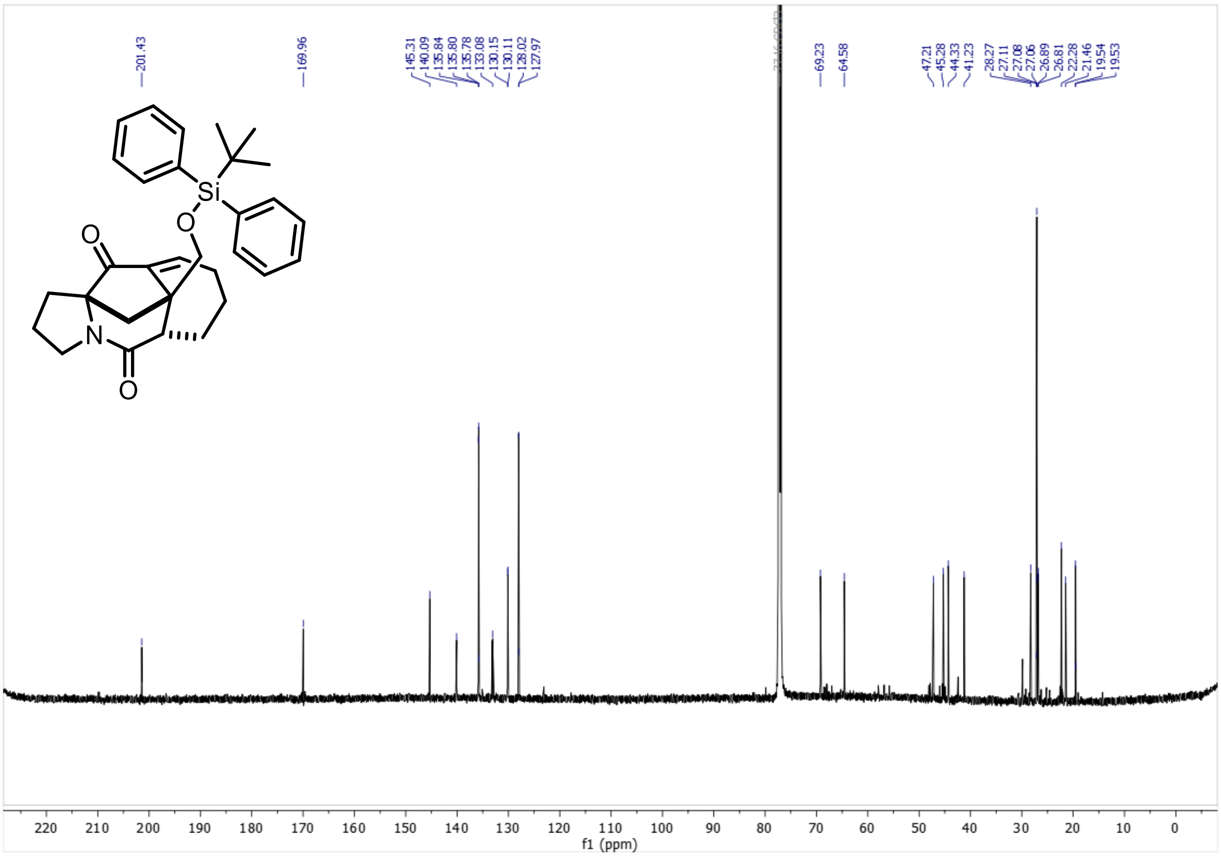

Compound 32 (^{1}H NMR, 400 MHz, $CDCl_3$)

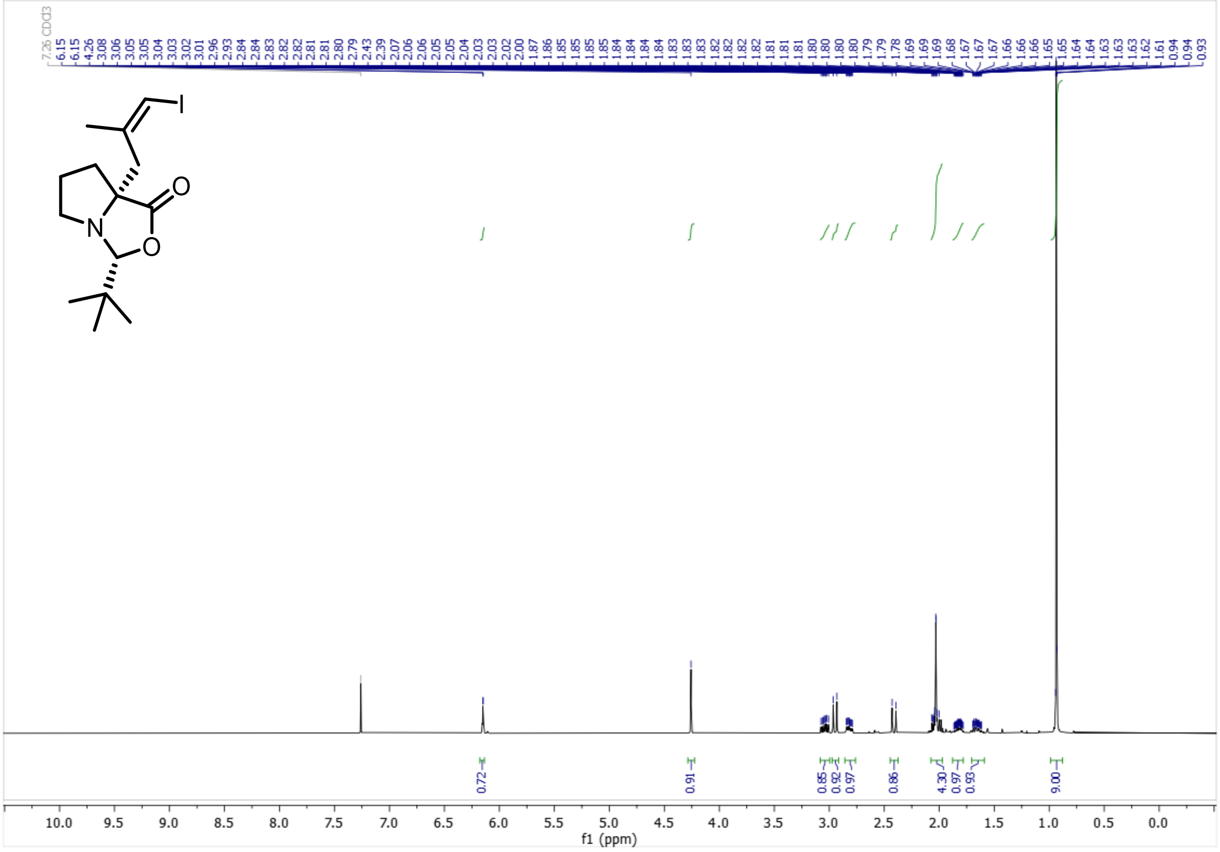

Compound 32 (^{13}C NMR, 101 MHz, $CDCl_3$)

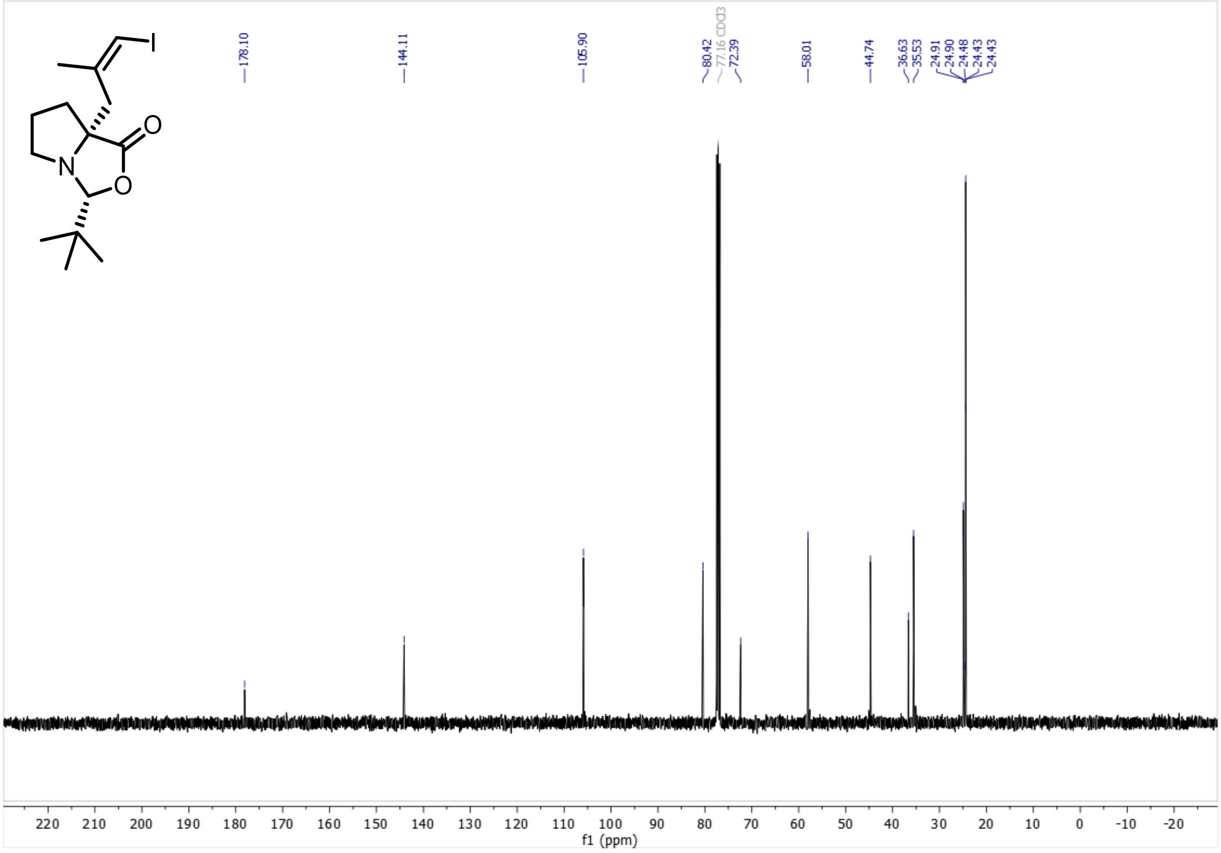

Compound 33 (^{1}H NMR, 400 MHz, $CDCl_3$)

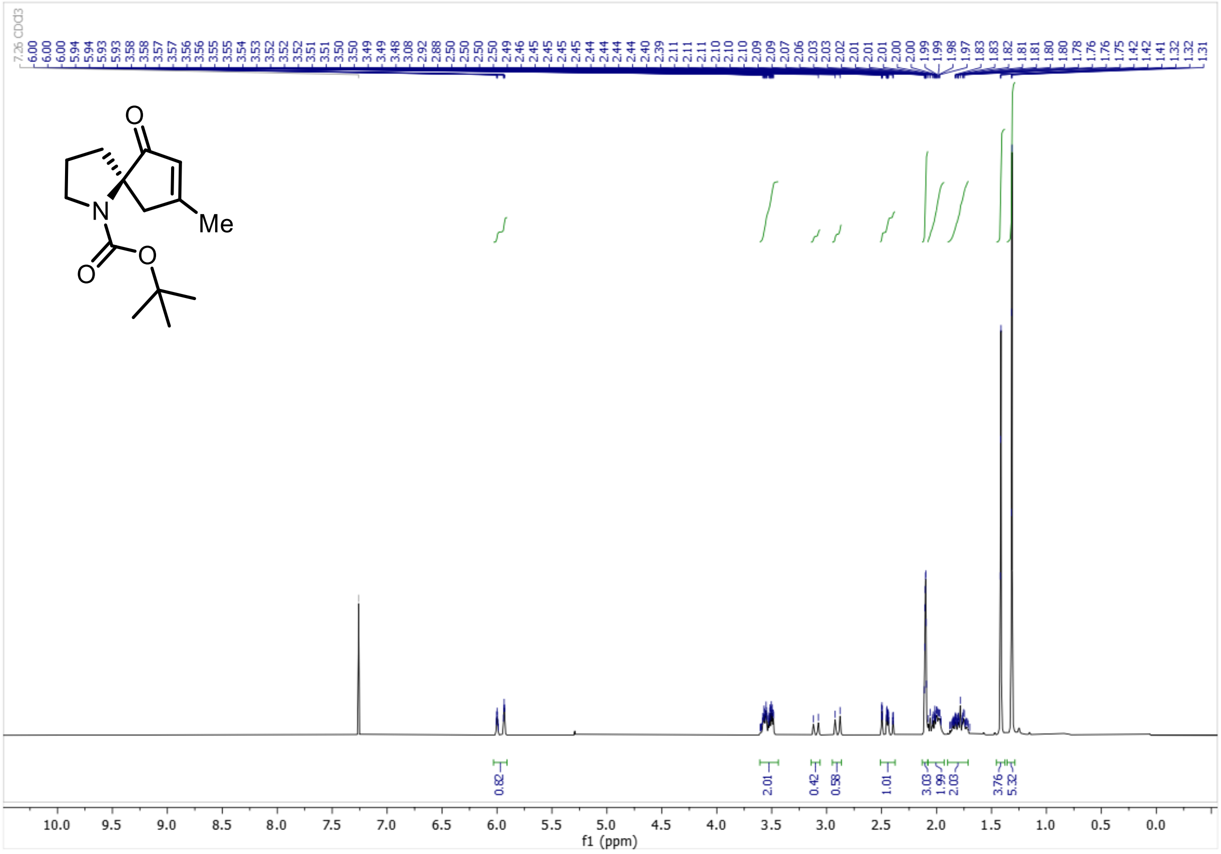

Compound 33 (^{13}C NMR, 101 MHz, $CDCl_3$)

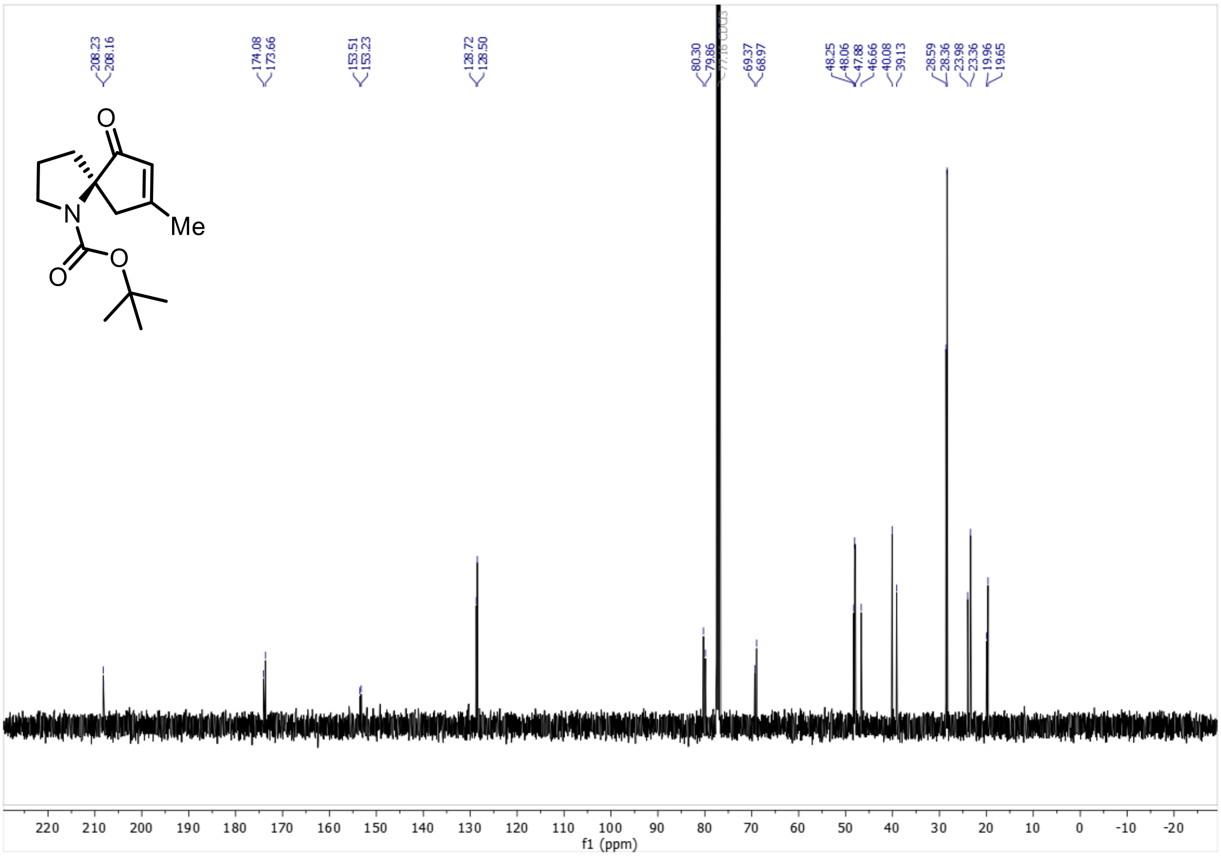

Compound S-15 (^1H NMR, 400 MHz, $CDCl_3$)

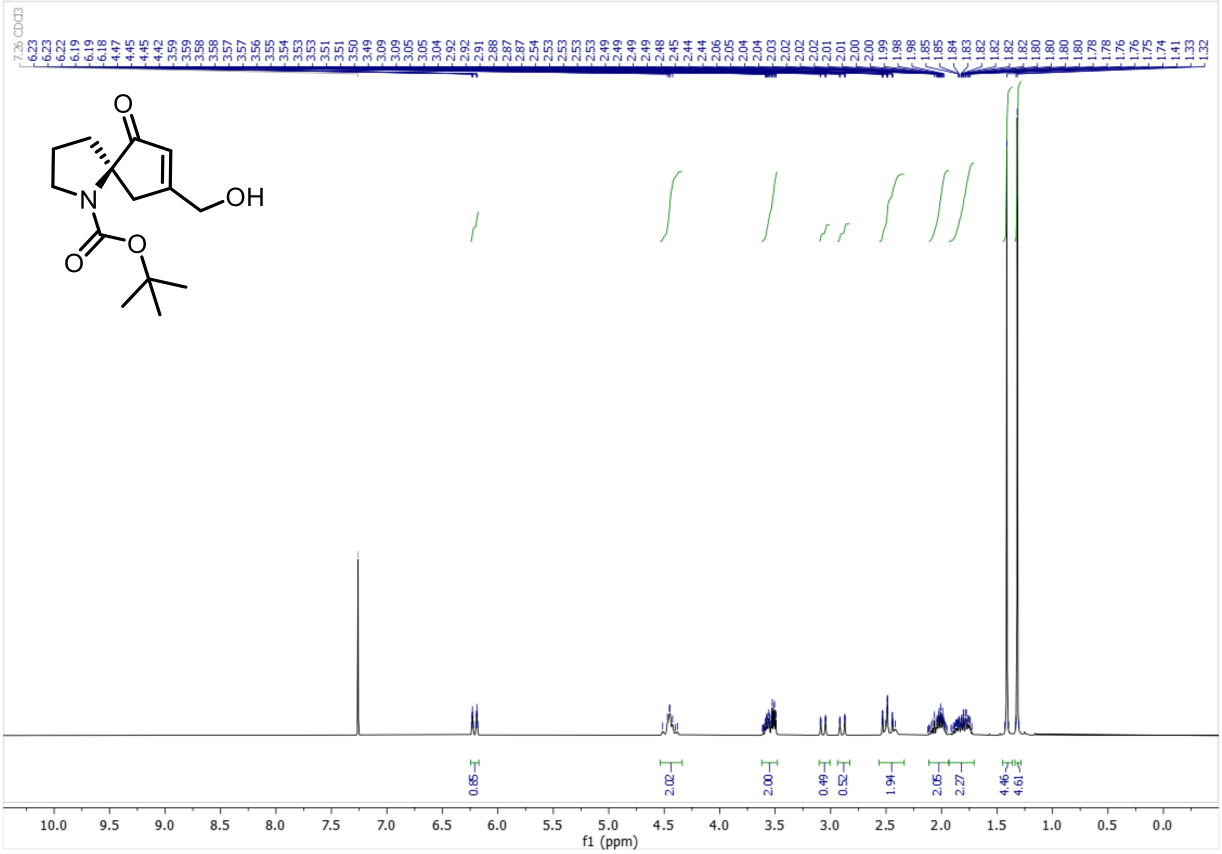

Compound S-15 (^{13}C NMR, 101 MHz, $CDCl_3$)

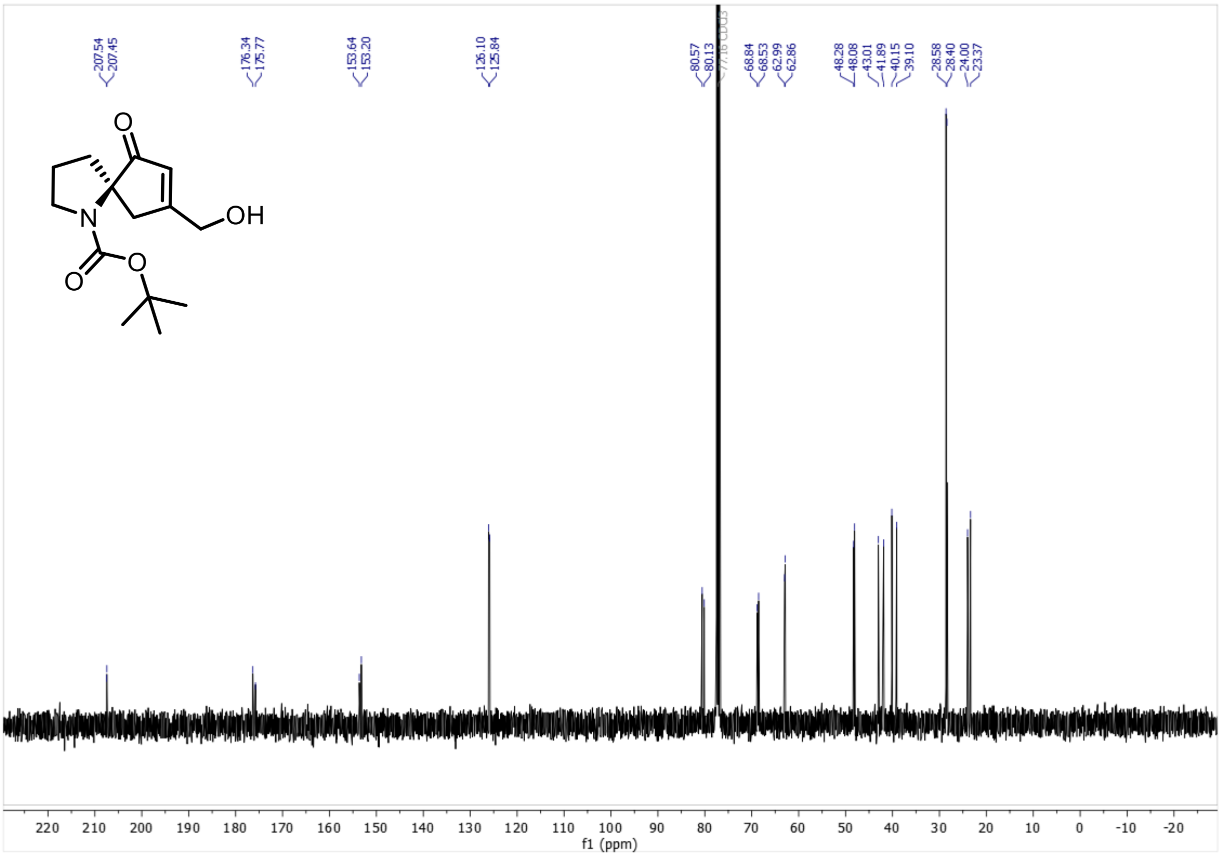

References

(1) Ben-Lulu, M.; Gaster, E.; Libman, A.; Pappo, D. Synthesis of Biaryl-Bridged Cyclic Peptides via Catalytic Oxidative Cross-Coupling Reactions. *Angew. Chem. - Int. Ed.* **2020**, *59*, 4835–4839.

(2) Shennan, B. D. A.; Smith, P. W.; Ogura, Y.; Dixon, D. J. A Modular and Divergent Approach to Spirocyclic Pyrrolidines. *Chem. Sci.* **2020**, *11*, 10354–10360.

(3) Gella, C.; Ferrer, È.; Alibés, R.; Busqué, F.; De March, P.; Figueredo, M.; Font, J. A Metal-Free General Procedure for Oxidation of Secondary Amines to Nitrones. *J. Org. Chem.* **2009**, *74*, 6365–6367.

(4) Larrosa, I.; Da Silva, M. I.; Gómez, P. M.; Hannen, P.; Ko, E.; Lenger, S. R.; Linke, S. R.; White, A. J. P.; Wilton, D.; Barrett, A. G. M. Highly Convergent Three Component Benzyne Coupling: The Total Synthesis of Ent-Clavilactone B. *J. Am. Chem. Soc.* **2006**, *128*, 14042–14043.

(5) Wright, A. C.; Lee, C. W.; Stoltz, B. M. Progress toward the Enantioselective Synthesis of Curcusones A-D via a Divinylcy-clopropane Rearrangement Strategy. *Org. Lett.* **2019**, *21*, 9658–9662.

(6) Taniguchi, T.; Tanabe, G.; Muraoka, O.; Ishibashi, H. Total Synthesis of (±)-Stemonamide and (±)-Isostemonamide Using a Radical Cascade. *Org. Lett.* **2008**, *10*, 197–199.

(7) Dagoneau, D.; Wang, Q.; Zhu, J. Towards the Sarpagine-Ajmaline-Macroline Family of Indole Alkaloids: Enantioselective Synthesis of an N-Demethyl Alstolactone Diastereomer. *Chem. Eur. J.* **2020**, *26*, 4866–4873.

(8) Elmarrouni, A.; Ritts, C. B.; Balsells, J. Silyl-Mediated Photoredox-Catalyzed Giese Reaction: Addition of Non-Activated Alkyl Bromides. *Chem. Sci.* **2018**, *9*, 6639–6646.

(9) Seebach, D.; Boes, M.; Naef, R.; Schweizer, W. B. Alkylation of Amino Acids without Loss of the Optical Activity: Preparation of -Substituted Proline Derivatives. A Case of Self-Reproduction of Chirality. *J. Am. Chem. Soc.* **1983**, *105*, 5390–5398.

(10) Rhoades, D.; Rheingold, A. L.; O'Malley, B. W.; Wang, J. Expedient Total Syntheses of Pladienolide-Derived Spliceosome Modulators. *J. Am. Chem. Soc.* **2021**, *143*, 4915–4920.

(11) Ding, X.-B.; Brimble, M. A.; Furkert, D. P. Reactivity of 2-Nitropyrrole Systems: Development of Improved Synthetic Approaches to Nitropyrrole Natural Products. *J. Org. Chem.* **2018**, 83, 12460−12470

(12) Horn, A.; Kazmaier, U. Purified MCPBA, a Useful Reagent for the Oxidation of Aldehydes. *European J. Org. Chem.* **2018**, *2018*, 2531–2536.
